# Genetic Alteration Profiles and Clinicopathological Associations in Atypical Parathyroid Adenoma

**DOI:** 10.1155/2021/6666257

**Published:** 2021-03-09

**Authors:** Xinxin Mao, Yan Wu, Shuangni Yu, Jie Chen

**Affiliations:** Department of Pathology, Peking Union Medical College Hospital, Chinese Academy of Medical Sciences and Peking Union Medical College, 1 Shuai Fu Yuan Hu Tong, Beijing 100730, China

## Abstract

Genomic aberrations associated with atypical parathyroid adenoma (AA) are poorly understood. Thus, herein, we sought to expand our current understanding of the molecular basis of atypical parathyroid adenomas. We analyzed 134 samples that had been surgically obtained from parathyroid tumors, including parathyroid carcinomas, atypical parathyroid adenomas, and parathyroid adenomas. The tumors were harvested from formalin-fixed, paraffin-embedded tissues. Fifteen tumor-related genes from recently published genome sequencing data were subjected to targeted sequencing analysis, and an average sequencing depth of 500x was achieved. Sixteen (16/50, 32%) AA tumors harbored at least one of the following genomic alterations: *CDC73* (12, 24%), *EZH2* (4, 8%), *HIC1* (1, 2%), and *CDKN2A* (1, 2%). Our study identified, for the first time, a relatively high frequency of genomic alterations in patients with AA in a Chinese population. This suggests that AA arises *de novo*, rather than developing from a parathyroid adenoma. Altogether, these findings will improve our understanding of the malignant potential of parathyroid tumors at the molecular level.

## 1. Introduction

Atypical parathyroid adenoma (AA) exhibits some of the features of parathyroid carcinoma but lacks its uncontrolled invasive growth. AA has uncertain malignant potential and exhibits morphological features that lie between those of benign parathyroid adenoma (PA) and parathyroid carcinoma (PC) [1]. Although the plasma levels of calcium and parathyroid hormone (PTH) are often higher in patients with PC or AA than in patients with PA, they are not sufficiently different to allow for differential diagnosis. Histopathology is the diagnostic gold standard, and uncontrolled invasive growth—as evidenced by capsular penetration and definitive invasion into adjacent tissue, vascular permeation, perineural spread, or metastases—has been specified as a criterion for PC diagnosis in the 4th edition of the World Health Organization (WHO) classification system published in 2017. Tumors with atypical features that do not meet the criteria for carcinoma can be classified as AA. The histopathological features of AA include banding, fibrosis, adherence to a contiguous structure, envelopment of the tumor within a capsule, solid or trabecular growth patterns, nuclear atypia, prominent nucleoli and mitotic activity, and no metastasis or invasion into the surrounding tissues. However, there is little agreement among pathologists regarding the histology-based diagnosis of AA, and none of the histopathological features mentioned above—either individually or in combination—can be used to diagnose the malignancy [2]. PC and PA are also associated with completely different prognoses. With respect to PC, the clinical findings are generally nonspecific; most recur locally and spread to contiguous structures in the neck, and recovery can only be achieved by complete surgical resection. In contrast, the overall rate of recovery from PA is excellent, and recurrence is rare. Because it has no typical pathological characteristics in terms of morphology, some cases of AA have been mistakenly diagnosed as PA, and such patients may require close monitoring. These patients may harbor malignancies that are not identified until there is distant tumor metastasis or recurrence; thus, a suitable strategy for diagnosing AA is needed.

The importance of genetic testing for diagnosis has increased alongside recent advancements in clinical molecular research; accordingly, a considerable amount of information regarding the molecular changes underlying tumors is now available. Advancements in molecular biology—especially the development of targeted gene sequencing, an inexpensive technique with relatively high sequence coverage—have allowed the analysis of whole genomes, exomes, and targeted sequences. Genome sequencing has been widely used over the past decade and has provided abundant insights into the pathogenesis of PA and PC. Molecular alterations associated with parathyroid tumors include genomic aberrations in cell division cycle 7 (*CDC7*) [3–5], cyclin D1 (*CCND1*) [6–10], cyclin*-*dependent kinase inhibitor (*CDKI*) [11], and multiple endocrine neoplasia type 1 (*MEN1*) [12–17]. Nevertheless, the molecular changes in AA are not well understood. It is not known whether the disease arises *de novo* or from PA; therefore, identifying the molecular events occurring at the initial stages of parathyroid cancer is necessary for obtaining a better understanding of the molecular mechanisms underlying parathyroid carcinogenesis. Moreover, understanding the molecular aberrations in AA is crucial for the careful follow-up of patients.

Therefore, in this study, we analyzed the genetic profiles of AA and attempted to identify changes in the expression of AA-related genes at the genome level. We analyzed formalin-fixed paraffin-embedded (FFPE) specimens using next-generation sequencing- (NGS-) based target sequencing with an ion proton-based cancer panel platform.

## 2. Materials and Methods

### 2.1. Patients and Samples

This study was approved by the Ethics Committee of the Peking Union Medical College Hospital, Beijing. Consent was obtained from each patient after fully explaining the purpose and nature of all procedures used. A total of 134 tumor samples from patients with PA (*n* = 50), AA (*n* = 50), and PC (*n* = 34)—which were retrospectively collected from the archives of the Peking Union Medical College Hospital from July 1997 to December 2017—were analyzed. None of the patients had received chemotherapy or radiotherapy prior to surgery. Two pathologists reviewed each hematoxylin and eosin (H&E) slide to determine the diagnoses. Patients with evidence of capsular penetration or vascular invasion, local invasion, and distant metastasis were included in the PC group; patients with tumors with atypical features that did not meet the criteria for carcinoma were included in the AA group; and those with benign tumors were included in the PA group. The median follow-up period was 96 months. Serial sections were cut from paraffin blocks, stained with H&E for routine histological examination, and classified according to the criteria published in the 4th edition of the WHO classification system. The clinicopathological data (sex, age, tumor location, tumor size, plasma calcium levels, and serum PTH levels) of the patients were retrieved from their records after obtaining the relevant ethical approvals, and the samples were subjected to molecular analyses.

### 2.2. DNA Extraction and Targeted Sequencing

DNA was isolated from paraffin-embedded samples using the T Guide FFPE DNA One-Step Kit (Tiangen Biotech, Beijing, China) according to the manufacturer's instructions. The quantity and quality of the extracted DNA were assessed using a NanoDrop 2000 spectrophotometer (Thermo Fisher Scientific, Waltham, MA). Clinically relevant gene alterations in the parathyroid tumor samples were detected by performing NGS using an Illumina HiSeq 2 × 150 bp platform (Illumina, San Diego, CA). Reads marked as polymerase chain reaction (PCR) or optical duplicates were removed from FastX (http://hannonlab.cshl.edu/fastx_toolkit/index.html). PC variants were called from the targeted exons, and off-target reads of up to 25 nucleotides in the introns were used to capture the potential splice site alterations. ANNOVAR (http://annovar.openbioinformatics.org) was used to annotate the functional consequences of variants and to quickly find the most biologically significant variant among the public databases (dbSNP, 1000 Genomes Project, ESP6500, ExAC03, and GENESKYDB_Freq).

### 2.3. Targeted DNA Panel Design and Sequencing Analysis

All FFPE samples were subjected to targeted NGS using a custom designed multigene panel, which included 15 genes selected from recently published genome sequencing data covering the exons of *CDC73*, *CCND1*, *MEN1*, *CDKN1B*, enhancer of zeste homolog 2 (*EZH2*), *CTNNB1*, *RASSF1*, *SFRP1*, *SFRP2*, *SFRP4*, *CDKN1B*, *CDKN2A*, *CDKN2B*, *WT-1*, and hypermethylated in cancer 1 (*HIC1*) [4, 7, 14, 18–23]. The genomic alterations in the exon regions mainly comprised single-nucleotide variations (SNVs) and insertions and deletions (INDELs). Base-calling, alignment to the UCSC hg19 human reference genome, and variant calling were performed using Torrent Suite software v.5.0 (Thermo Fisher Scientific), and called variants were annotated using GATK (https://software.broadinstitute.org/gatk/best-practices/). Variant filtering was performed by correcting the error alignment caused by INDELs and basic group quality correction. This step is the key to more accurate identification of SNVs and INDELs, as it greatly reduces the rates of false-positive and false-negative results produced during sequencing and comparison.

Sanger sequencing was used to confirm all gene alterations in this study. Each exon was sequenced bidirectionally using specific PCR primers and an ABI Prism Big Dye Terminator v3.1 Cycle Sequencing Kit (Applied Biosystems, Foster City, CA). Sequencing primer extension reactions were analyzed using an ABI 3730XL Genetic Analyzer (Applied Biosystems) according to the manufacturer's instructions.

## 3. Results

### 3.1. Clinicopathological Characteristics of the Series

**Figure 1:**
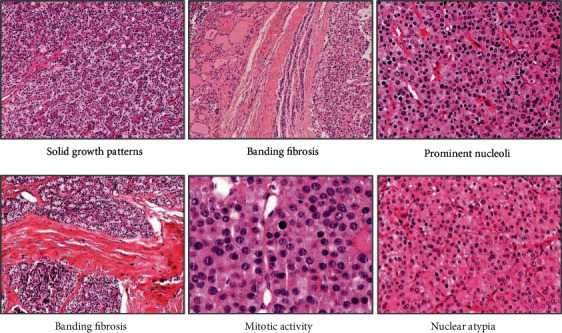
Histopathological characteristics of atypical parathyroid adenomas.

Clinicopathological data from 134 patients are presented in Table 1. One patient with AA and multiple endocrine neoplasia type 1 (MEN1) and a patient with PC and hyperparathyroidism-jaw tumor syndrome (HPT-JT) were identified. Each patient underwent neck surgery. The symptoms and signs included neck mass, fatigue, bone pain, and depression. The main clinical manifestations were renal (nephrocalcinosis, nephrolithiasis, and kidney failure), bone-related (osteitis fibrosa cystica, osteoporosis, and pathological fractures), or both. A total of 50 patients with AA were analyzed, and their histopathological characteristics are presented in Figure 1. The median age, median serum PTH level, and median highest serum calcium level of patients with AA were 49.6 years (range, 14–78 years), 657.34 (81.2–2500 pg/mL), and 2.73 mg/dL (1.06–3.83 mg/dL), respectively. Most of the parathyroid tumors were solid, but a few were cystic (8.16%). At the last follow-up, 132 of the patients with parathyroid tumors still had the disease, and two patients had died of PC.

### 3.2. Quality Assessment of the Targeted Sequences

**Figure 2:**
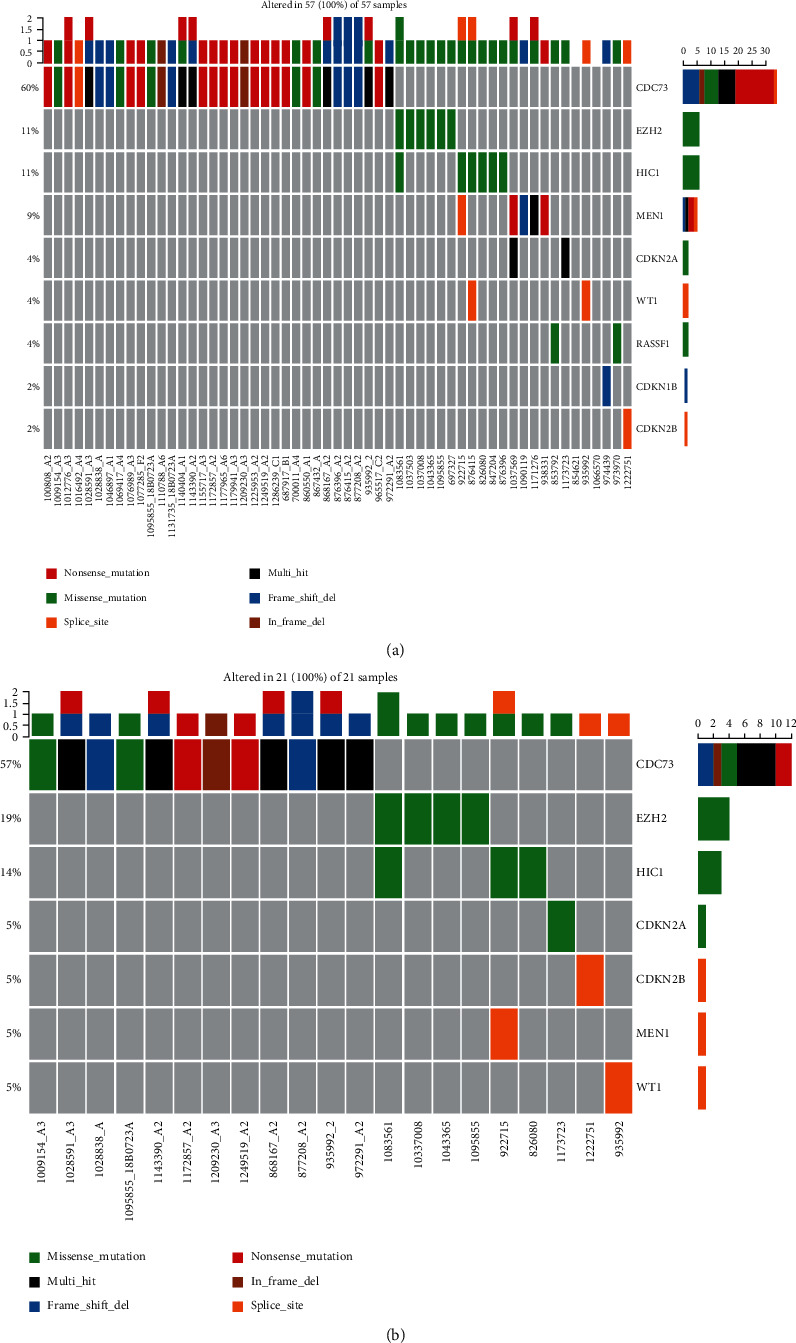
(a) and (b). Genetic landscapes of 50 atypical parathyroid adenomas and all 134 parathyroid tumor samples.

We achieved an average target sequencing depth of 500x in all samples. After multiple-step filtering, 50 genomic alterations were identified in all samples, including 22 missense alterations, 16 nonsense alterations, five splicing alterations, two in-frame INDELs, and seven frameshift alterations (Figure 2).

DNA sequences for the 15 selected genes were successfully amplified from all samples using multiplex PCR, and adequate libraries were obtained for deep sequencing. All samples had a Q30 value higher than 80%, with a 90% average ratio of reads mapped to target regions, and the average percentage of sequencing uniformity (the proportion of sequences that was 0.2-folds higher than the mean coverage) reached 90%. On average, 15 million mapped reads were generated for each subject, and 48.5% of the reads were on target for all patients with parathyroid cancer (Supplemental Table 1).

### 3.3. Recurrent Genomic Alterations among Patients with AA

We investigated the relationship between different parathyroid tumor types and tumor-related somatic gene alterations; details of the gene alterations in the samples are listed in Table 2. The number of genetic variants detected in each sample varied among the different sample types. In the whole cohort, 47 samples (35%) exhibited genomic alterations, 34 (72%) were affected by one alteration, and 13 (28%) were affected by more than one alteration. Molecular alterations were also confirmed by Sanger sequencing (Supplemental Figure 1).

Alterations in AA genes were identified in 32% of the samples (16/50) at different frequencies. Alterations in *CDC73* were the most frequent (24% of the samples; 12/50), followed by alterations in *EZH2* (*n* = 4, 8%), *HIC1* (*n* = 1, 2%), and *CDKN2A* (*n* = 1, 2%) (Table 2). *EZH2* alterations (c.1451C>A: p.Pro484Gln) were observed in one sample harboring the *CDC73* alteration (c.191T>C: p.Leu64Pro), with *HIC1* (c.1571A>G: p.Lys524Arg) and *EZH2* alterations (c.1936T>A: p.Tyr646Asn) identified in the same sample. The patient with MEN1 syndrome (ID 039, Table 2) harbored two alterations in *CDC73*. Three types of *EZH2* alterations (c.647G>A: p.Arg216Gln, c.1451C>A: p.Pro484Gln, and c.1936T>A: p.Tyr646Asn) were detected in four AA samples, and Tyr646Asn was recurrently identified in two patients with AA. A single type of *CDKN2A* alteration (c.343G>T: p.Val115Leu) was detected in an AA sample, and a single type of *HIC1* alteration (c.1571A>G: p.Lys524Arg) was confirmed in an AA tumor.

### 3.4. Genomic Alterations in PC and PA

Of the 34 patients with PC, 16 had sporadic primary PC that was adequately treated with initial surgical resection (all had definitive capsular penetration or vascular permeation), two had a recurrent local disease, and 16 had metastatic PC (four patients with lung metastases, one patient with liver metastasis, and 11 patients with definitive invasion into adjacent tissues, such as soft tissue and the thyroid gland). Furthermore, 19 *CDC73* alterations were observed in 18 (53%) PC samples (Supplemental Table 3)—including four samples exhibiting tumor invasion into the adjacent tissue and three lesions—followed by alterations in *EZH2* (*n* = 2, 6%) and *HIC1* (*n* = 2, 6%). The proportions of *CDC73* alterations in the invasive lesions and in recurrent local disease samples were not significantly different. The patient with HPT-JT syndrome (ID 028, Supplemental Table 3) harbored one alteration in *CDC73*. Eleven different alterations were detected in the PA samples (11/50, 22%); alterations in *MEN1* and *CDC73* were predominant (4 lesions, 8%), followed by *RASSF1* (*n* = 1, 2%), *CDKN2A* (*n* = 1, 2%), *CDKN1B* (*n* = 1, 2%), and *HIC1* (*n* = 1, 2%) (Supplemental Table 4). In one lesion, a *MEN1* alteration was observed together with a *CDKN2A* alteration.

## 4. Discussion

Our understanding of the molecular pathogenesis of parathyroid tumors, especially PC, has increased significantly over the last two decades. However, there has been no major progress in understanding the molecular characteristics and landscape of AA, and their clinical implications have not been fully elucidated. Herein, we attempted to identify potential genomic alterations in a series of AA cases in a Chinese population. NGS employed in this study achieved substantially improved sensitivity and accuracy, with an average sequencing depth that exceeded 500x. Our analysis of 134 parathyroid tumor samples in the ion proton-based cancer panel revealed routine alterations in parathyroid tumor-related genes. NGS alteration analysis revealed 16 (32%) samples with genomic alterations among 50 sporadic surgically resected AA tumors. We also detected PA and PC lesions that were devoid of experimental deviations and found that the gene profile of AA more closely resembles the gene profile of PC than of PA.

Somatic, intragenic, and inactivating alterations of *CDC73* are the most common variant in parathyroid tumors [24]. The inactivation of *CDC73*, and its gene product parafibromin, is a major driver of parathyroid cancer. Herein, numerous *CDC73* alterations were detected in (34/134; 25%) parathyroid tumors; of these, 51% were nonsense mutations, 29% INDELs, and 20% missense mutations, and these results were consistent with those reported previously [25]. Notably, our results revealed that AA is associated with a lower *CDC73* alteration rate (*n* = 12, 24%) than PC (*n* = 18, 53%), but this rate was higher than that of PA (*n* = 4, 8%), suggesting that specific somatic *CDC73* mutations are important in AA, which may account for its relatively aggressive biological behavior. Somatic *CDC73* mutation screening in AA was only performed in a few studies [26–28]. However, an interesting study involving parathyroid-specific Cdc73-knockout mice—in which one or both Cdc73 alleles were deleted, resulting in the development of parathyroid tumors—revealed nuclear pleomorphism, fibrous septa, and overexpression of galectin-3 in 75% of patients, consistent with the histological diagnosis of AA. Moreover, the mice exhibited a significantly increased parathyroid tumor proliferation rate [29]. It should be noted that in this study, one patient with PC exhibited a HPT-JT syndrome. Previous studies have revealed that *CDC73* mutations are present in approximately 90% of patients with HPT-JT and in a third of patients with apparently sporadic PC, suggesting a high risk of developing HPT-JT-related tumors. In this study, only one patient with PC harbored a *CDC73* mutation (c.70G>T: p.Glu24∗) owing to the rarity of the disease.

Herein, one patient with AA presented with familial MEN1 syndrome characterized by AA and pituitary adenoma; the patient harbored two somatic *CDC73* alterations (c.9_18delCGTGCTTAGC: p.Asp3Glufs∗15, c.571delG: p.Ala191Leufs∗11) but lacked *MEN1* mutation. A recent paper reported a similar *MEN1* phenocopy due to a *CDC73* mutation [30], and to date, only three cases of AA have been associated with MEN1 syndrome; two carried pathogenic variants of *MEN1* [31–33]. MEN1 primary hyperparathyroidism is now well established owing to its role in benign parathyroid lesions or PA. Two whole-exome capture and high-throughput sequencing studies of PA revealed *MEN1* alterations as a major driver, with an alteration frequency of 35% [13, 14], and mutations of the gene have also been identified in AA and PC [34, 35]. In this study, the *MEN1* mutation occurred at a rate of 8% in PA, whereas no such alterations were detected in PC or AA. Although the proportion was low, it was still a high mutation rate in PA. This suggests that *MEN1* alterations occur early during parathyroid tumor formation, which is a wider presentation of parathyroid disease.

We found that more than 10% of patients with AA had at least one alteration in addition to the *CDC73* mutation. Among these patients, the most frequently altered gene was *EZH2*, which encodes the histone methylase H3K27—an epigenetic silencer that regulates gene expression in numerous cancers [36]. By modulating gene expression, *EZH2* promotes the survival, proliferation, and invasion of cancer cells, in addition to enhancing the development of drug resistance among these cells [37–39]. *EZH2* is aberrantly overexpressed in various malignant tumors, such as prostate, breast, and ovarian cancers [40]. Activating mutations of *EZH2* oncogenes have been identified in benign PA using whole-exome sequencing [14]. The same mutations have been found in malignant tumors of the blood and ovary [41]. Here, the rate of *EZH2* aberrations was 8% in AA and 6% in PC; no alterations were detected in the PA samples, and one recurrent missense mutation (c.1936T>A: p.Tyr646Asn) was detected in two patients with AA. The Tyr646Asn mutation in *EZH2* is frequently identified in both follicular lymphoma and pediatric-type nodal follicular lymphoma in adult patients [42].

The inactivation of CDKIs—including members of the *CDKN1* and *CDKN2* families—is thought to play a role in tumor suppression. *CDKN1B*, which encodes p27, is the most extensively studied member of the *CDKN1* family with respect to parathyroid tumors [43]. Somatic *CDKN1B* alterations, observed in a few cases of sporadic PA, result in protein instability by essentially eliminating expression [12]. *CDKN2A*, which encodes P16^INK4A^ (p16), is located within the frequently deleted chromosomal region 9 of p21 [44]. Silencing of the *CDKN2A* tumor suppressor gene is causally associated with several cancer types. Several genetic and epigenetic aberrations of *CDKN2A* lead to enhanced tumorigenesis and metastasis with recurrence of cancer and poor prognosis.

We further investigated genes that are rarely studied in parathyroid tumors, such as *HIC1*, which is a tumor suppressor gene (17p13.3) that is frequently deleted or epigenetically silenced by DNA methylation. Herein, the rate of *HIC-1* alteration was 2% in both AA and PA. *HIC1* was generally underexpressed, regardless of the hyperparathyroidism, including multiple parathyroid tumors in the same patient. Overexpression of *HIC1* leads to a decrease in the clonogenic survival of parathyroid tumor cells, strongly supporting a regulatory role for *HIC1* in the growth of parathyroid glands [23].

## 5. Conclusions

AA comprises a group of uncertain potentially malignant tumors, with unclear molecular mechanisms underlying their biological behavior. To the best of our knowledge, this study is the first attempt at exploring the genetic changes in AA using targeted NGS, followed by a comparison with those in PC and PA to provide further insights into the molecular biology of AA. Sixteen AA tumors harbored at least one of the genomic alterations including *CDC73*, *EZH2*, *HIC1*, and *CDKN2A*. *CDC73* alterations occurred frequently in AA samples, indicating the aggressiveness of AA. Furthermore, frequent changes in the oncogene *EZH2* in the AA samples were similar to those in the PC, but not PA samples. Moreover, *MEN1*, *CDKN1B*, and *RASSF1* alterations were present in the PA samples but absent in the PC and AA samples. One patient with AA and MEN1 and a patient with PC and HPT-JT were identified. Our findings suggest that AA has relatively specific molecular features that are similar to those of PC. This explains the relatively aggressive behavior of AA and indicates that it may arise *de novo* during tumor progression.

## Figures and Tables

**Table 1:** Clinical features of the 134 patients with parathyroid tumors included in this study.

Tumor type	Adenoma	Atypical	Carcinoma
Case	50	50	34
Gender			
Male	15	19	24
Female	35	30	10
Age (median)	52.4 (29–79)	49.6 (14–78)	47.6 (24–76)
Tumor location			
Left	28	26	16
Right	22	23	18
Tumor size (median)	1.85	2.83	2.58
Plasma calcium level (median) (2.13–2.70 mmol/L)	2.64	2.73	2.99
PTH level (median) (12.0–65.0 pg/mL)	580.33	657.34	940.64
Distant metastasis			
Present	—	—	16
Disease recurrence			
Yes	—	—	2
No	50	50	32
Survival			
Alive	50	50	32
Dead			2

**Table 2:** Clinical features of patients with AA from the study cohort with called genomic variants, reference sequences, and SNP-ID.

ID	Tumor size	Serum Ca (mmol/L) (2.13–2.70 mmol/L)	PTH (pg/mL) (12.0–65.0 pg/mL)	Gene	cDNA change	Protein change	Reference sequence	SNP-ID
037	2.2	3.29	828	CDC73	c.754delA	p.Ile252Phefs∗5	NM_024529	
					c.85G>T	p.Glu29∗	NM_024529	
039	2.4	3.83	425	CDC73	c.571delG	p.Ala191Leufs∗11	NM_024529	
					c.9_18delCGTGCTTAGC	p.Asp3Glufs∗15	NM_024529	
043	3	3.18	226	CDC73	c.362C>T	p.Ser121Phe	NM_024529	rs121434263
				CDC73	c.128G>A	p.Trp43∗	NM_024529	
048	3.5	2.85	2500	CDC73	c.85delG	p.Glu29Serfs∗8	NM_024529	rs587776560
				CDC73	c.175dupT	p.Ser59Phefs∗7	NM_024529	
051	4	3.44	1769	CDC73	c.232G>A	p.Ala78Thr	NM_024529	
052	5	1.06	186	CDC73	c.10delG	p.Val4Cysfs∗17	NM_024529	
					c.664C>T	p.Arg222∗	NM_024529	
053	1.2	1.36	444	CDC73	c.84_90delGGAGTTC	p.Glu29Profs∗6	NM_024529	
056	2	2.75	219	EZH2	c.647G>A	p.Arg216Gln	NM_004456	rs747028969
057	1.5	3.1	318	EZH2	c.1936T>A	p.Tyr646Asn	NM_004456	rs267601395
062	1	1.37	81.2	EZH2	c.1936T>A	p.Tyr646Asn	NM_004456	rs267601395
				HIC1	c.1571A>G	p.Lys524Arg	NM_006497	
063	2	2.98	192	CDC73	c.191T>C	p.Leu64Pro	NM_024529	rs121434264
				EZH2	c.1451C>A	p.Pro484Gln	NM_004456	
068	2	2.98	612	CDC73	c.549delT	p.Ala184Glnfs∗18	NM_024529	
				CDC73	c.25C>T	p.Arg9∗	NM_024529	rs121434262
071	2.4	3.29	162	CDC73	c.128G>A	p.Trp43∗	NM_024529	rs121434263
072	1.5	1.55	167	CDKN2A	c.343G>T	p.Val115Leu	NM_000077	rs779913365
074	6	4.46	1231	CDC73	c.195_203delATAACGTGC	p.Asn65_His68delinsAsn	NM_024529	
082	3.5	3.29	1224	CDC73	c.1394C>A	p.Ser465∗	NM_024529	

ID 039: MEN1 syndrome case.

## Data Availability

All research data used to support the findings of this study are available from the corresponding author upon request.

## Supplementary Materials

Supplementary 1 Supplemental Figure 1 Sanger sequencing results of all alterations in the parathyroid tumor samples.

Supplementary 2 Supplemental Table 1: read depth and quality assessment of the targeted sequencing data in each lesion. Supplemental Table 2: details all alterations in the parathyroid tumor samples. Supplemental Table 3: clinical features of patients with PC from the study cohort with called genomic variants, reference sequences, and SNP-ID. Supplemental Table 4: clinical features of patients with PA from the study cohort with called genomic variants, reference sequences, and SNP-ID.

## References

[1] DeLellis R. A. (2011). Parathyroid tumors and related disorders. *Modern Pathology*.

[2] Kumari N., Chaudhary N., Pradhan R., Agarwal A., Krishnani N. (2016). Role of histological criteria and immunohistochemical markers in predicting risk of malignancy in parathyroid neoplasms. *Endocrine Pathology*.

[3] Newey P. J., Bowl M. R., Cranston T., Thakker R. V. (2010). Cell division cycle protein 73 homolog (CDC73) mutations in the hyperparathyroidism-jaw tumor syndrome (HPT-JT) and parathyroid tumors. *Human Mutation*.

[4] Costa-Guda J., Arnold A. (2014). Genetic and epigenetic changes in sporadic endocrine tumors: parathyroid tumors. *Molecular and Cellular Endocrinology*.

[5] Yu W., McPherson J. R., Stevenson M. (2015). Whole-exome sequencing studies of parathyroid carcinomas reveal novel PRUNE2 mutations, distinctive mutational spectra related to APOBEC-catalyzed DNA mutagenesis and mutational enrichment in kinases associated with cell migration and invasion. *Journal of Clinical Endocrinology and Metabolism*.

[6] Motokura T., Bloom T., Kim H. G. (1991). A novel cyclin encoded by a _bcl1_ -linked candidate oncogene. *Nature*.

[7] Westin G., Björklund P., Akerström G. (2009). Molecular genetics of parathyroid disease. *World Journal of Surgery*.

[8] Imanishi Y., Hosokawa Y., Yoshimoto K. (2001). Primary hyperparathyroidism caused by parathyroid-targeted overexpression of cyclin D1 in transgenic mice. *The Journal of Clinical Investigations*.

[9] Vasef M. A., Brynes R. K., Sturm M., Bromley C., Robinson R. A. (1999). Expression of cyclin D1 in parathyroid carcinomas, adenomas, and hyperplasias: a paraffin immunohistochemical study. *Modern Pathology*.

[10] Zhao L., Sun L. H., Liu D. M. (2014). Copy number variation in CCND1 gene is implicated in the pathogenesis of sporadic parathyroid carcinoma. *World Journal of Surgery*.

[11] Costa-Guda J., Marinoni I., Molatore S., Pellegata N. S., Arnold A. (2011). Somatic Mutation and germline sequence abnormalities inCDKN1B, encoding p27Kip1, in sporadic parathyroid adenomas. *The Journal of Clinical Endocrinology and Metabolism*.

[12] Libutti S. K., Crabtree J. S., Lorang D. (2003). Parathyroid gland-specific deletion of the mouse Men1 gene results in parathyroid neoplasia and hypercalcemic hyperparathyroidism. *Cancer Result*.

[13] Newey P. J., Nesbit M. A., Rimmer A. J. (2012). Whole-exome sequencing studies of nonhereditary (sporadic) parathyroid adenomas. *The Journal of Clinical Endocrinology and Metabolism*.

[14] Cromer M. K., Starker L. F., Choi M. (2012). Identification of somatic Mutations in parathyroid tumors using whole-exome sequencing. *The Journal of Clinical Endocrinology and Metabolism*.

[15] Costa-Guda J., Imanishi Y., Palanisamy N. (2013). Allelic imbalance in sporadic parathyroid carcinoma and evidence for its de novo origins. *Endocrine*.

[16] Dwight T., Twigg S., Delbridge L. (2000). Loss of heterozygosity in sporadic parathyroid tumours: involvement of chromosome 1 and the MEN1 gene locus in 11q13. *Clinical Endocrinology*.

[17] Haven C. J., van Puijenbroek M., Karperien M., Fleuren G. J., Morreau H. (2004). Differential expression of the calcium sensing receptor and combined loss of chromosomes 1q and 11q in parathyroid carcinoma. *The Journal of Pathology*.

[18] Costa-Guda J., Marinoni I., Molatore S., Pellegata N. S., Arnold A. (2011). Somatic mutation and germline sequence abnormalities in CDKN1B, encoding p27Kip1, in sporadic parathyroid adenomas. *The Journal of Clinical Endocrinology and Metabolism*.

[19] Gill A. J. (2014). Understanding the genetic basis of parathyroid carcinoma. *Endocrine Pathology*.

[20] Starker L. F., Svedlund J., Udelsman R. (2011). The DNA methylome of benign and malignant parathyroid tumors. *Genes, Chromosomes and Cancer*.

[21] Sulaiman L., Juhlin C. C., Nilsson I. L., Fotouhi O., Larsson C., Hashemi J. (2014). Global and gene-specific promoter methylation analysis in primary hyperparathyroidism. *Epigenetics*.

[22] Svedlund J., Koskinen Edblom S., Marquez V. E., Åkerström G., Björklund P., Westin G. (2012). Hypermethylated in cancer 1 (HIC1), a Tumor suppressor gene epigenetically deregulated in hyperparathyroid Tumors by histone H3 lysine modification. *The Journal of Clinical Endocrinology and Metabolism*.

[23] Svedlund J., Barazeghi E., Stålberg P. (2014). The histone methyltransferase EZH2, an oncogene common to benign and malignant parathyroid tumors. *Endocrine-Related Cancer*.

[24] Shattuck T. M., Välimäki S., Obara T. (2003). Somatic and germ-line mutations of theHRPT2Gene in sporadic parathyroid carcinoma. *New England Journal of Medicine*.

[25] Cardoso L., Stevenson M., Thakker R. V. (2017). Molecular genetics of syndromic and non-syndromic forms of parathyroid carcinoma. *Human Mutation*.

[26] Bradley K. J., Cavaco B. M., Bowl M. R., Harding B., Young A., Thakker R. V. (2005). Utilisation of a cryptic non-canonical donor splice site of the gene encoding PARAFIBROMIN is associated with familial isolated primary hyperparathyroidism. *Journal of Medical Genetics*.

[27] Guarnieri V., Battista C., Muscarella L. A. (2012). CDC73 mutations and parafibromin immunohistochemistry in parathyroid tumors: clinical correlations in a single-centre patient cohort. *Cellular Oncology*.

[28] Kelly T. G., Shattuck T. M., Reyes-Mugica M. (2006). Surveillance for early detection of aggressive parathyroid disease: carcinoma and atypical adenoma in familial isolated hyperparathyroidism associated with a germline HRPT2 mutation. *Journal of Bone and Mineral Research*.

[29] Walls G. V., Stevenson M., Lines K. E. (2017). Mice deleted for cell division cycle 73 gene develop parathyroid and uterine tumours: model for the hyperparathyroidism-jaw tumour syndrome. *Oncogene*.

[30] Lines K. E., Nachtigall L. B., Dichtel L. E. (2020). Multiple Endocrine Neoplasia Type 1 (MEN1) Phenocopy Due to a Cell Cycle Division 73 (CDC73) Variant. *Journal of the Endocrine Society*.

[31] Christakis I., Busaidy N. L., Cote G. J. (2016). Parathyroid carcinoma and atypical parathyroid neoplasms in MEN1 patients; A clinico-pathologic challenge. The MD Anderson case series and review of the literature. *International Journal of Surgery*.

[32] Cetani F., Marcocci C., Torregrossa L., Pardi E. (2019). Atypical parathyroid adenomas: challenging lesions in the differential diagnosis of endocrine tumors. *Endocrine-Related Cancer*.

[33] Cinque L., Pugliese F., Clemente C. (2020). Rare Somatic MEN1 Gene Pathogenic Variant in a Patient Affected by Atypical Parathyroid Adenoma. *International Journal of Endocrinology*.

[34] Cinque L., Sparaneo A., Salcuni A. S. (2017). MEN1 gene mutation with parathyroid carcinoma: first report of a familial case. *Endocrine Connections*.

[35] Brewer K., Costa-Guda J., Arnold A. (2019). Molecular genetic insights into sporadic primary hyperparathyroidism. *Endocrine-Related Cancer*.

[36] Simon J. A., Lange C. A. (2008). Roles of the EZH2 histone methyltransferase in cancer epigenetics. *Mutation Research*.

[37] Kim K. H., Roberts C. W. M. (2016). Targeting EZH2 in cancer. *Nature Medicine*.

[38] Yamagishi M., Uchimaru K. (2017). Targeting EZH2 in cancer therapy. *Current Opinion in Oncology*.

[39] Pasini D., Di Croce L. (2016). Emerging roles for Polycomb proteins in cancer. *Current Opinion in Genetics & Development*.

[40] Völkel P., Dupret B., Le Bourhis X., Angrand P. O. (2015). Diverse involvement of EZH2 in cancer epigenetics. *American Journal of Translational Research*.

[41] Morin R. D., Johnson N. A., Severson T. M. (2010). Somatic mutations altering EZH2 (Tyr641) in follicular and diffuse large B-cell lymphomas of germinal-center origin. *Nature Genetics*.

[42] Lovisa F., Binatti A., Coppe A. (2019). A high definition picture of key genes and pathways mutated in pediatric follicular lymphoma. *Haematologica*.

[43] Costa-Guda J., Soong C.-P., Parekh V. I., Agarwal S. K., Arnold A. (2013). Germline and somatic alterations in cyclin-dependent kinase inhibitor genes CDKN1A, CDKN2B, and CDKN2C in sporadic parathyroid adenomas. *Hormones & Cancer*.

[44] Gil J., Peters G. (2006). Regulation of the *INK4b-ARF-INK4a* tumour suppressor locus: all for one or one for all. *Nature reviews Molecular cell biology*.

