# Supplementary material for: Genetic Alteration Profiles and Clinicopathological Associations in Atypical Parathyroid Adenoma
Source: Int J Genomics. 2021 Mar 9;2021:6666257. doi: 10.1155/2021/6666257 (PMC7969847; doi:10.1155/2021/6666257)
Supplement: Supplementary 1 — Supplemental Figure 1 Sanger sequencing results of all alterations in the parathyroid tumor samples. [file 6666257.f1.zip › Supplemental Table 1 (1).pdf]

Supplemental Table 1. Read depth and quality assessment of the targeted sequencing data in each lesion

Sample	Clean Reads	Raw Reads	Clean Reads Ratio	Q20	Q30
1	482961	514131	0.94	99.94%	93.31%
2	349353	384125	0.91	99.94%	93.06%
3	426184	464916	0.92	99.93%	93.05%
4	185602	213441	0.87	99.92%	93.52%
5	451763	493119	0.92	99.94%	93.58%
6	371074	406961	0.91	99.93%	92.85%
7	282541	316453	0.89	99.95%	93.12%
8	470111	507406	0.93	99.93%	93.41%
9	350609	387205	0.91	99.93%	93.06%
10	391911	425891	0.92	99.93%	93.19%
11	452093	494333	0.91	99.94%	93.19%
12	478422	510026	0.94	99.94%	92.82%
13	412340	450184	0.92	99.94%	93.13%
14	196480	247546	0.79	99.94%	92.53%
15	407986	444105	0.92	99.94%	92.67%
16	268428	292755	0.92	99.94%	92.94%
17	394959	438617	0.9	99.94%	93.14%
18	318633	351149	0.91	99.92%	93.18%
19	492944	528802	0.93	99.94%	93.24%
20	446003	486930	0.92	99.94%	92.69%
21	271317	306118	0.89	99.94%	92.82%
22	500088	534596	0.94	99.95%	93.23%
23	358311	387369	0.92	99.94%	92.50%
24	418367	454049	0.92	99.94%	92.80%
25	432436	482818	0.9	99.95%	93.72%
26	200452	226971	0.88	99.91%	93.62%
27	175379	214420	0.82	99.94%	92.64%
28	466168	505210	0.92	99.93%	93.47%
29	113525	139922	0.81	99.91%	93.30%
30	229671	260156	0.88	99.94%	93.12%
31	211324	249275	0.85	99.94%	93.14%
32	412930	470029	0.88	99.94%	93.72%
33	385355	425269	0.91	99.94%	93.62%
34	418725	448088	0.93	99.97%	86.31%
35	205903	233624	0.88	99.98%	85.56%
36	433459	469456	0.92	99.97%	86.26%
37	422736	462595	0.91	99.97%	85.94%
38	414952	455717	0.91	99.97%	85.76%
39	381192	412789	0.92	99.97%	86.03%
40	453203	484366	0.94	99.97%	86.74%
41	471910	508322	0.93	99.97%	86.03%
42	427337	466173	0.92	99.97%	86.19%
43	174420	208250	0.84	99.96%	86.03%
44	459408	498610	0.92	99.97%	86.72%

45	458623	500458	0.92	99.97%	85.47%
46	482873	520288	0.93	99.98%	86.04%
47	459053	497943	0.92	99.97%	86.21%
48	361547	401786	0.9	99.95%	86.23%
49	389120	421385	0.92	99.97%	85.23%
50	338840	379492	0.89	99.98%	85.73%
51	235619	260530	0.9	99.97%	85.94%
52	266839	303198	0.88	99.97%	85.44%
53	497214	535001	0.93	99.98%	85.14%
54	318902	343598	0.93	99.97%	86.18%
55	499321	536949	0.93	99.97%	86.37%
56	506109	549191	0.92	99.98%	85.40%
57	219228	262141	0.84	99.97%	85.95%
58	384304	426694	0.9	99.98%	85.94%
59	470168	509342	0.92	99.97%	86.37%
60	391176	430073	0.91	99.97%	84.88%
61	444686	478849	0.93	99.97%	85.08%
62	436087	477005	0.91	99.97%	86.19%
63	457518	495702	0.92	99.97%	86.71%
64	470337	512968	0.92	99.98%	85.58%
65	318412	359810	0.88	99.98%	86.17%
66	382481	422598	0.91	99.98%	86.08%
67	439921	480187	0.92	99.97%	86.70%
68	378156	414616	0.91	99.97%	86.01%
69	219083	248137	0.88	99.97%	85.89%
70	419207	461139	0.91	99.97%	85.82%
71	446879	489513	0.91	99.97%	85.44%
72	224122	256887	0.87	99.97%	85.14%
73	430934	473840	0.91	99.97%	86.42%
74	481843	518751	0.93	99.97%	86.75%
75	518988	559439	0.93	99.98%	86.04%
76	472234	515836	0.92	99.97%	86.36%
77	467866	505436	0.93	99.97%	86.82%
78	487844	525489	0.93	99.97%	86.92%
79	458089	499773	0.92	99.95%	85.88%
80	506918	549068	0.92	99.97%	86.55%
81	414754	452372	0.92	99.97%	85.95%
82	417675	458807	0.91	99.97%	86.11%
83	509305	549506	0.93	99.97%	86.26%
84	390270	431249	0.9	99.97%	85.60%
85	413140	447206	0.92	99.97%	86.44%
86	526563	565088	0.93	99.97%	86.63%
87	477974	520425	0.92	99.97%	86.25%
88	355559	383107	0.93	99.98%	80.47%
89	497147	531491	0.94	99.97%	86.66%
90	540462	580906	0.93	99.97%	86.07%
91	409080	453643	0.9	99.98%	86.12%
92	442268	481908	0.92	99.97%	86.67%

93	480670	519005	0.93	99.97%	86.38%
94	395234	436214	0.91	99.97%	87.25%
95	468420	509815	0.92	99.97%	85.98%
96	206696	223014	0.93	99.97%	86.29%
97	247496	267648	0.92	99.96%	86.61%
98	219415	237004	0.93	99.97%	86.09%
99	371027	409414	0.91	99.97%	86.33%
100	170477	200811	0.85	99.97%	85.42%
101	315645	348371	0.91	99.98%	86.12%
102	221514	255435	0.87	99.97%	86.26%
103	153148	176586	0.87	99.97%	86.42%
104	234564	267962	0.88	99.98%	86.63%
105	399006	440504	0.91	99.97%	86.09%
106	348553	388061	0.9	99.97%	86.23%
107	375579	416326	0.9	99.97%	85.22%
108	270100	305122	0.89	99.97%	85.90%
109	278046	311873	0.89	99.98%	85.51%
110	355430	393135	0.9	99.98%	85.58%
111	303796	342472	0.89	99.97%	86.62%
112	82921	105082	0.79	99.98%	86.31%
113	389560	431049	0.9	99.97%	85.80%
114	144562	178496	0.81	99.98%	85.54%
115	318586	351768	0.91	99.97%	86.37%
116	453390	490606	0.92	99.98%	85.26%
117	340412	380353	0.89	99.97%	86.38%
118	406471	448791	0.91	99.98%	86.06%
119	476392	517404	0.92	99.97%	86.48%
120	113921	144675	0.79	99.97%	85.79%
121	531183	569092	0.93	99.97%	86.40%
122	103117	123488	0.84	99.98%	85.88%
123	409825	449480	0.91	99.97%	85.91%
124	435369	473348	0.92	99.98%	86.31%
125	432625	471043	0.92	99.97%	86.51%
126	318156	351467	0.91	99.97%	85.82%
127	409606	451174	0.91	99.97%	85.92%
128	404551	447606	0.9	99.97%	85.49%
129	378651	409840	0.92	99.98%	86.36%
130	398116	431010	0.92	99.97%	85.34%
131	375097	400614	0.94	99.97%	86.19%
