# Supplementary material for: Genetic Alteration Profiles and Clinicopathological Associations in Atypical Parathyroid Adenoma
Source: Int J Genomics. 2021 Mar 9;2021:6666257. doi: 10.1155/2021/6666257 (PMC7969847; doi:10.1155/2021/6666257)
Supplement: Supplementary 1 — Supplemental Figure 1 Sanger sequencing results of all alterations in the parathyroid tumor samples. [file 6666257.f1.zip › Supplemental Table 2 (1).pdf]

Supplemental Table 2: Details of all alterations in pararthyroid tumors

SNV NO.	Gene	Predicted Protein Variants	irst Priorit
SNV00048	CDKN2A	L,CDKN2A:NM_001195132:exon2:c.G343T:p.V115L,C	Second
SNV00053	EZH2	9Q,EZH2:NM_001203248:exon12:c.C1409A:p.P470Q,EZ	First1
SNV00054	EZH2	6Q,EZH2:NM_001203248:exon7:c.G620A:p.R207Q,EZ	First1
SNV00003	CDC73	CDC73:NM_024529:exon1:c.3delG:p.M1fs	First1
SNV00004	CDC73	CDC73:NM_024529:exon1:c.9_18del:p.D3fs	First1
SNV00005	CDC73	CDC73:NM_024529:exon1:c.10delG:p.V4fs	First1
SNV00006	CDC73	CDC73:NM_024529:exon1:c.18delC:p.S6fs	First1
SNV00007	CDC73	CDC73:NM_024529:exon1:c.C25T:p.R9X	First1
SNV00008	CDC73	CDC73:NM_024529:exon1:c.45delG:p.K15fs	First1
SNV00009	CDC73	CDC73:NM_024529:exon1:c.G64T:p.G22X	First1
SNV00010	CDC73	CDC73:NM_024529:exon1:c.G70T:p.E24X	First1
SNV00011	CDC73	CDC73:NM_024529:exon1:c.82delG:p.G28fs	First1
SNV00012	CDC73	CDC73:NM_024529:exon1:c.84_90del:p.G28fs	First1
SNV00013	CDC73	CDC73:NM_024529:exon1:c.G85T:p.E29X	First1
SNV00014	CDC73	CDC73:NM_024529:exon1:c.104_118del:p.35_40del	Second
SNV00015	CDC73	CDC73:NM_024529:exon1:c.G128A:p.W43X	First1
SNV00016	CDC73	NM_024529:exon1:c.131+1G>C	First1
SNV00017	CDC73	CDC73:NM_024529:exon2:c.G157T:p.E53X	First1
SNV00018	CDC73	CDC73:NM_024529:exon2:c.C162G:p.Y54X	First1
SNV00019	CDC73	CDC73:NM_024529:exon2:c.T163A:p.Y55N	First1
SNV00020	CDC73	CDC73:NM_024529:exon2:c.174dupT:p.D58fs	First1
SNV00021	CDC73	CDC73:NM_024529:exon2:c.T191C:p.L64P	First1
SNV00022	CDC73	CDC73:NM_024529:exon2:c.194_202del:p.65_68del	First1
SNV00023	CDC73	CDC73:NM_024529:exon2:c.C226T:p.R76X	First1
SNV00024	CDC73	CDC73:NM_024529:exon2:c.G232C:p.A78P	First1
SNV00025	CDC73	CDC73:NM_024529:exon3:c.259_260del:p.R87fs	First1
SNV00026	CDC73	CDC73:NM_024529:exon3:c.T293C:p.L98P	First1
SNV00027	CDC73	CDC73:NM_024529:exon4:c.C362T:p.S121F	First1
SNV00028	CDC73	CDC73:NM_024529:exon7:c.549delT:p.A183fs	First1
SNV00029	CDC73	CDC73:NM_024529:exon7:c.570delG:p.M190fs	First1
SNV00030	CDC73	CDC73:NM_024529:exon7:c.C664T:p.R222X	First1
SNV00031	CDC73	CDC73:NM_024529:exon8:c.753delA:p.A251fs	First1
SNV00032	CDC73	CDC73:NM_024529:exon8:c.G817A:p.A273T	First1
SNV00033	CDC73	CDC73:NM_024529:exon14:c.G1305A:p.M435I	First1
SNV00034	CDC73	CDC73:NM_024529:exon15:c.C1394A:p.S465X	First1
SNV00035	MEN1	98L,MEN1:NM_130801:exon10:c.C1493T:p.P498L,ME	First1
SNV00036	MEN1	65+6G>A;NM_130803:exon9:c.1365+6G>A;NM_13080	First2
SNV00037	MEN1	54X,MEN1:NM_130801:exon9:c.G1360T:p.G454X,MEN	First1
SNV00038	MEN1	54X,MEN1:NM_130801:exon7:c.C1060T:p.Q354X,ME	First1
SNV00039	MEN1	3fs,MEN1:NM_130801:exon3:c.503_510del:p.G168fs,M	First1
SNV00040	MEN1	45X,MEN1:NM_130804:exon3:c.133G>T:p.E45X,MEN	First1
SNV00042	CDKN1B	CDKN1B:NM_004064:exon1:c.280delC:p.P94fs	First1
SNV00043	HIC1	3202:exon2:c.G818A:p.S273N,HIC1:NM_006497:exon2:	First1

SNV00044	HIC1	xon2:c.1217C>T:p.A406V,HIC1:NM_001098202:exon2:	Third
SNV00045	HIC1	02:exon2:c.A1628G:p.K543R,HIC1:NM_006497:exon2:	First1
SNV00046	HIC1	xon2:c.2002G>A:p.A668T,HIC1:NM_001098202:exon2:	First1
SNV00052	EZH2	N,EZH2:NM_001203247:exon16:c.T1921A:p.Y641N,E	First1

Calling Q	otyping Qu	VCF Filter	Case_het	Case_hom	Chrs	Position	Ref Allele
M	1		1037569,1173723,		9	2.2E+07	C
H	1		1095855,		7	1.5E+08	G
H	0.9516		1037008,		7	1.5E+08	C
M	1		1131735_18B0723A,		1	1333-1930	G
H	1		877208_A2,		1	1339-1930	TGCTTA
M	1		1028591_A3,		1	1340-1930	G
M	1		1046897_A1,		1	1348-1930	C
M	1		1143390_A2,		1	1.9E+08	C
H	1		876396_A2,876415_A2,		1	1375-1930	G
H	0.9846		860550_A1,		1	1.9E+08	G
M	0.9923		1177965_A6,		1	1.9E+08	G
M	1		972291_A2,		1	1412-1930	G
H	0.9923		1028838_A,		1	1414-1930	GGAGTTC
M	1		7_A2,1155717_A3,128623		1	1.9E+08	G
H	0.9923		1110788_A6,		1	1434-1930	GAAGACC
H	0.9923		935992_2,1172857_A2,		1	1.9E+08	G
H	0.9923		1016492_A4,		1	1.9E+08	G
H	1		1179941_A3,		1	1.9E+08	G
H	1		89_A3,1077285_F2,11404		1	1.9E+08	C
M	1		1069417_A4,		1	1.9E+08	T
H	1		972291_A2,		1	4283-1930	-
H	1		1095855_18B0723A,		1	1.9E+08	T
H	1		1209230_A3,		1	4304-1930	TAACGTC
H	1		1012776_A3,		1	1.9E+08	C
M	0.9545		1009154_A3,		1	1.9E+08	G
H	1		876396_A2,876415_A2,		1	9325-1930	AG
H	1		867432_A,		1	1.9E+08	T
M	0.9922		935992_2,		1	1.9E+08	C
H	1		1143390_A2,		1	1016-1931	T
M	1		877208_A2,		1	1037-1931	G
H	1		1028591_A3,687917_B1,		1	1.9E+08	C
H	1		868167_A2,		1	7020-1931	A
M	1		1140404_A1,		1	1.9E+08	G
H	0.9923		700011_A4,		1	1.9E+08	G
H	1		1249519_A2,		1	1.9E+08	C
H	1		1171276,		11	6.5E+07	G
H	1		922715,		11	6.5E+07	C
M	0.9924			938331,	11	6.5E+07	C
H	0.9924		1037569,		11	6.5E+07	G
H	1		1090119,		11	5522-6457	CAGGCC
H	0		1171276,		11	6.5E+07	C
H	0.9769		974439,		12	1053-1287	C
H	1		826080,		17	1960745	G

M	0		922715,		17	1961201	C
M	0.8397		847204,1083561,876396,		17	1961555	A
M	1		876415,		17	1961986	G
H	0.9924		3365,1083561,1037503,697		7	1.5E+08	A

Alt Allele	ene Regio	Function	trand Orie	tein Varia	SNP ID	q_Alt (100	000g_chb
A	exonic	nonsynonymous SNV	-		s779913365		
T	exonic	nonsynonymous SNV	-				
T	exonic	nonsynonymous SNV	-		s747028969		
-	exonic	frameshift deletion	+		1060500020		
-	exonic	frameshift deletion	+				
-	exonic	frameshift deletion	+				
-	exonic	frameshift deletion	+				
T	exonic	stopgain	+		4262(STR_around)		
-	exonic	frameshift deletion	+				
T	exonic	stopgain	+				
T	exonic	stopgain	+				
-	exonic	frameshift deletion	+		s587776560		
-	exonic	frameshift deletion	+				
T	exonic	stopgain	+		DEL_around)		
-	exonic	nonframeshift deletion	+				
A	exonic	stopgain	+		s121434263		
C	splicing		+				
T	exonic	stopgain	+				
G	exonic	stopgain	+		s121434265		
A	exonic	nonsynonymous SNV	+				
T	exonic	frameshift insertion	+				
C	exonic	nonsynonymous SNV	+		s121434264		
-	exonic	nonframeshift deletion	+				
T	exonic	stopgain	+		s886041158		
C	exonic	nonsynonymous SNV	+				
-	exonic	frameshift deletion	+				
C	exonic	nonsynonymous SNV	+				
T	exonic	nonsynonymous SNV	+				
-	exonic	frameshift deletion	+				
-	exonic	frameshift deletion	+				
T	exonic	stopgain	+				
-	exonic	frameshift deletion	+				
A	exonic	nonsynonymous SNV	+				
A	exonic	nonsynonymous SNV	+				
A	exonic	stopgain	+				
A	exonic	nonsynonymous SNV	-		s766604600		
T	splicing		-		s746077651		
A	exonic	stopgain	-				
A	exonic	stopgain	-				
-	exonic	frameshift deletion	-				
A	exonic	stopgain	-				
-	exonic	frameshift deletion	+				
A	exonic	nonsynonymous SNV	+				

T	exonic	nonsynonymous SNV	+				
G	exonic	nonsynonymous SNV	+				
A	exonic	nonsynonymous SNV	+		s99114111		
T	exonic	nonsynonymous SNV	-		s267601395		

ExAC03	xAC03_EA	esp6500	gnomAD	Hrcr1	Kaviar	NCI60	kyExonDB	yGenomeD
			2.1E-05					
8.3E-06	0		4.1E-06		6.5E-06			
					6.5E-06			
					6.5E-06			
					6.5E-06			
					6.5E-06			
2.3E-05	0.0002		1.9E-05		1.3E-05			
3.3E-05	0		2E-05		2.6E-05			

			2.9E-05					

onsSites	SIFT Score	T Score P	Phen V2	POLYPhen V2 Score Pred	utationTas
9;Name=V	0.15	T	0.826	P	0.575
	0.366	T	0.035	B	1
	0.035	D	0.943	P	0.998
					1
;Name=V$					1
;Name=V$					1
5;Name=V					
5;Name=V					
5;Name=V					1
5;Name=V					
					1
					1
					1
					1
	0	D	1	D	1
	0.001	D	0.999	D	1
Name=V$I					1
9;Name=V	0.005	D	1	D	1
	0	D	1	D	1
	0.044	D	0.351	B	1
4;Name=V					
1;Name=V					1
;Name=V$					
;Name=V$	0.582	T	0	B	0.97
	0.336	T	0.001	B	0.992
					1
	0.305	T	0.745	P	0.998
					1
					1
Name=V$					
Name=V$$					1
;Name=V$					
	0.542	T	0	B	1

8;Name=V	0.457	T	0.827	P	0.959
;Name=VS	0.312	T	0.989	D	1
);Name=V	0.003	D	0.197	B	1
;Name=VS	0	D	0.998	D	1

tionTaster	Cadd_Raw	Cadd_Phre	Dann	Eigen	V_ADA_	NV_RF_S	OMIM
D	3.34673	22.9	0.982	-0.4083			er, multiple, (3); Pancreati
D	2.0944	16.82	0.952	0.0481			1573|Weaver syndrome, 2
D	4.58931	24.4	0.999	0.159			1573|Weaver syndrome, 2
							tumor syndrome, 145001
							tumor syndrome, 145001
							tumor syndrome, 145001
							tumor syndrome, 145001
A	11.0718	37	0.997	0.72			tumor syndrome, 145001
							tumor syndrome, 145001
A	12.0687	38	0.995	1.1824			tumor syndrome, 145001
A	11.3774	37	0.996	0.8455			tumor syndrome, 145001
							tumor syndrome, 145001
							tumor syndrome, 145001
A	12.3716	39	0.996	1.1731			tumor syndrome, 145001
							tumor syndrome, 145001
A	10.4893	36	0.993	0.943			tumor syndrome, 145001
D	5.10071	25.3	0.995	1.0962	1	0.94	tumor syndrome, 145001
A	12.9906	40	0.998	1.2839			tumor syndrome, 145001
A	10.4471	36	0.997	0.7081			tumor syndrome, 145001
D	6.55299	31	0.992	0.9074			tumor syndrome, 145001
							tumor syndrome, 145001
A	6.09412	28.2	0.999	0.8127			tumor syndrome, 145001
							tumor syndrome, 145001
A	12.7298	40	0.998	0.7997			tumor syndrome, 145001
D	6.61626	32	0.998	1.0038			tumor syndrome, 145001
							tumor syndrome, 145001
D	6.20825	28.7	0.999	0.8781			tumor syndrome, 145001
D	3.83905	23.4	0.993	0.2209			tumor syndrome, 145001
							tumor syndrome, 145001
							tumor syndrome, 145001
A	12.2567	39	0.998	0.7367			tumor syndrome, 145001
							tumor syndrome, 145001
D	2.76974	21.2	0.967	-0.1764			tumor syndrome, 145001
D	2.65394	20.5	0.956	-0.2838			tumor syndrome, 145001
A	13.2175	41	0.994	1.0127			tumor syndrome, 145001
D	4.26484	23.9	0.995	-0.2837			nor of lung (3); Lipoma, so
	1.72815	14.57	0.865	1.4956	0.0009	0.012	nor of lung (3); Lipoma, so
D	12.0968	38	0.991	0.8357			nor of lung (3); Lipoma, so
A	11.32	37	0.998	1.027			nor of lung (3); Lipoma, so
							nor of lung (3); Lipoma, so
A	10.5589	36	0.997	0.9838			Parathyroid adenoma, son
							ltiple endocrine neoplasia,
N	1.18692	11.68	0.979	-0.5027			17p13.3|603825

N	2.01984	16.34	0.997	-0.1876			17p13.3|603825
D	3.64685	23.2	0.988	0.0058			17p13.3|603825
D	4.39505	24.1	0.998	-0.0435			17p13.3|603825
D	6.76401	32	0.985	1.07			1573|Weaver syndrome, 2

HPO	IGMD_sit	_si	HGMD_gene
ritance;Astrocytoma;Meningio			r deficiency;Melanoma, increase
equinovarus;Short ribs;Promin			ver syndrome;Weaver syndrome
equinovarus;Short ribs;Promin			ver syndrome;Weaver syndrome
Iypercalcemia;Parathyroid ade			ism-jaw tumour syndrome;Parat
Iypercalcemia;Parathyroid ade			ism-jaw tumour syndrome;Parat
Iypercalcemia;Parathyroid ade			ism-jaw tumour syndrome;Parat
Iypercalcemia;Parathyroid ade			ism-jaw tumour syndrome;Parat
Iypercalcemia;Parathyroid ade	lism-jaw t	DM	ism-jaw tumour syndrome;Parat
Iypercalcemia;Parathyroid ade			ism-jaw tumour syndrome;Parat
Iypercalcemia;Parathyroid ade			ism-jaw tumour syndrome;Parat
Iypercalcemia;Parathyroid ade	yroid carc	DM	ism-jaw tumour syndrome;Parat
Iypercalcemia;Parathyroid ade	lism-jaw t	DM	ism-jaw tumour syndrome;Parat
Iypercalcemia;Parathyroid ade			ism-jaw tumour syndrome;Parat
Iypercalcemia;Parathyroid ade	lism-jaw t	DM	ism-jaw tumour syndrome;Parat
Iypercalcemia;Parathyroid ade			ism-jaw tumour syndrome;Parat
Iypercalcemia;Parathyroid ade			ism-jaw tumour syndrome;Parat
Iypercalcemia;Parathyroid ade			ism-jaw tumour syndrome;Parat
Iypercalcemia;Parathyroid ade	thyroidism	DM	ism-jaw tumour syndrome;Parat
Iypercalcemia;Parathyroid ade			ism-jaw tumour syndrome;Parat
Iypercalcemia;Parathyroid ade	thyroidism	DM	ism-jaw tumour syndrome;Parat
Iypercalcemia;Parathyroid ade			ism-jaw tumour syndrome;Parat
Iypercalcemia;Parathyroid ade	lism-jaw t	DM	ism-jaw tumour syndrome;Parat
Iypercalcemia;Parathyroid ade			ism-jaw tumour syndrome;Parat
Iypercalcemia;Parathyroid ade	lism-jaw t	DM	ism-jaw tumour syndrome;Parat
Iypercalcemia;Parathyroid ade			ism-jaw tumour syndrome;Parat
Iypercalcemia;Parathyroid ade	yroid carc	DM	ism-jaw tumour syndrome;Parat
Iypercalcemia;Parathyroid ade	thyroidism	DM	ism-jaw tumour syndrome;Parat
Iypercalcemia;Parathyroid ade			ism-jaw tumour syndrome;Parat
Iypercalcemia;Parathyroid ade			ism-jaw tumour syndrome;Parat
Iypercalcemia;Parathyroid ade	yroid carc	DM	ism-jaw tumour syndrome;Parat
Iypercalcemia;Parathyroid ade	thyroid ca	DM	ism-jaw tumour syndrome;Parat
Iypercalcemia;Parathyroid ade			ism-jaw tumour syndrome;Parat
Iypercalcemia;Parathyroid ade			ism-jaw tumour syndrome;Parat
Iypercalcemia;Parathyroid ade			ism-jaw tumour syndrome;Parat
Iypercalcemia;Parathyroid ade			ism-jaw tumour syndrome;Parat
ypercortisolism;Exocrine panc			sulinomas;Multiple endocrine ne
ypercortisolism;Exocrine panc			sulinomas;Multiple endocrine ne
ypercortisolism;Exocrine panc			sulinomas;Multiple endocrine ne
ypercortisolism;Exocrine panc	ndocrine n	DM	sulinomas;Multiple endocrine ne
ypercortisolism;Exocrine panc			sulinomas;Multiple endocrine ne
ypercortisolism;Exocrine panc	ndocrine n	DM	sulinomas;Multiple endocrine ne
umor;Parathyroid adenoma;At			vergrowth and neurodevelopmen

equinovarus;Short ribs;Promir			ver syndrome;Weaver syndrome

InterVar	InterVar_evidence	erpro_dom
Uncertain significance	, 0, 0, 0, 0, 0] PP=[0, 0, 0, 0, 0, 0] BA1=0 BS=[0,	
Uncertain significance	, 0, 0, 0, 0, 0] PP=[0, 1, 0, 0, 0, 0] BA1=0 BS=[0,	
Likely pathogenic	, 0, 0, 0, 0, 0] PP=[0, 1, 1, 0, 0, 0] BA1=0 BS=[0,	
Uncertain significance	, 0, 0, 0, 0, 0] PP=[0, 0, 0, 0, 1, 0] BA1=0 BS=[0,	
Uncertain significance	, 0, 0, 0, 0, 0] PP=[0, 0, 0, 0, 0, 0] BA1=0 BS=[0,	
Uncertain significance	, 0, 0, 0, 0, 0] PP=[0, 0, 0, 0, 0, 0] BA1=0 BS=[0,	
Uncertain significance	, 0, 0, 0, 0, 0] PP=[0, 0, 0, 0, 0, 0] BA1=0 BS=[0,	
Uncertain significance	, 0, 0, 0, 0, 0] PP=[0, 0, 1, 0, 1, 0] BA1=0 BS=[0,	
Uncertain significance	, 0, 0, 0, 0, 0] PP=[0, 0, 0, 0, 0, 0] BA1=0 BS=[0,	
Uncertain significance	, 0, 0, 0, 0, 0] PP=[0, 0, 1, 0, 0, 0] BA1=0 BS=[0,	
Uncertain significance	, 0, 0, 0, 0, 0] PP=[0, 0, 1, 0, 0, 0] BA1=0 BS=[0,	
Uncertain significance	, 0, 0, 0, 0, 0] PP=[0, 0, 0, 0, 1, 0] BA1=0 BS=[0,	
Uncertain significance	, 0, 0, 0, 0, 0] PP=[0, 0, 0, 0, 0, 0] BA1=0 BS=[0,	
Uncertain significance	, 0, 0, 0, 0, 0] PP=[0, 0, 1, 0, 1, 0] BA1=0 BS=[0,	
Uncertain significance	, 0, 1, 0, 0, 0] PP=[0, 0, 0, 0, 0, 0] BA1=0 BS=[0,	
Uncertain significance	, 0, 0, 0, 0, 0] PP=[0, 0, 1, 0, 1, 0] BA1=0 BS=[0,	
Pathogenic	, 0, 0, 0, 0, 0] PP=[0, 0, 1, 0, 0, 0] BA1=0 BS=[0,	
Pathogenic	, 0, 0, 0, 0, 0] PP=[0, 0, 1, 0, 0, 0] BA1=0 BS=[0,	
Pathogenic	, 0, 0, 0, 0, 0] PP=[0, 0, 1, 0, 1, 0] BA1=0 BS=[0,	
Uncertain significance	, 0, 0, 0, 0, 0] PP=[0, 0, 1, 0, 0, 0] BA1=0 BS=[0,	
Likely pathogenic	, 0, 0, 0, 0, 0] PP=[0, 0, 0, 0, 0, 0] BA1=0 BS=[0,	
Likely pathogenic	, 0, 0, 0, 0, 0] PP=[0, 0, 1, 0, 1, 0] BA1=0 BS=[0,	
Uncertain significance	, 0, 1, 0, 0, 0] PP=[0, 0, 0, 0, 0, 0] BA1=0 BS=[0,	
Pathogenic	, 0, 0, 0, 0, 0] PP=[0, 0, 1, 0, 1, 0] BA1=0 BS=[0,	
Uncertain significance	, 0, 0, 0, 0, 0] PP=[0, 0, 1, 0, 0, 0] BA1=0 BS=[0,	
Likely pathogenic	, 0, 0, 0, 0, 0] PP=[0, 0, 0, 0, 0, 0] BA1=0 BS=[0,	
Uncertain significance	, 0, 0, 0, 0, 0] PP=[0, 0, 1, 0, 0, 0] BA1=0 BS=[0,	
Uncertain significance	, 0, 0, 0, 0, 0] PP=[0, 0, 0, 0, 0, 0] BA1=0 BS=[0,	
Likely pathogenic	, 0, 0, 0, 0, 0] PP=[0, 0, 0, 0, 0, 0] BA1=0 BS=[0,	
Likely pathogenic	, 0, 0, 0, 0, 0] PP=[0, 0, 0, 0, 0, 0] BA1=0 BS=[0,	
Pathogenic	, 0, 0, 0, 0, 0] PP=[0, 0, 1, 0, 0, 0] BA1=0 BS=[0,	
Likely pathogenic	, 0, 0, 0, 0, 0] PP=[0, 0, 0, 0, 0, 0] BA1=0 BS=[0,	
Uncertain significance	, 0, 0, 0, 0, 0] PP=[0, 0, 0, 0, 0, 0] BA1=0 BS=[0,	
Uncertain significance	, 0, 0, 0, 0, 0] PP=[0, 0, 0, 0, 0, 0] BA1=0 BS=[0,	
Pathogenic	, 0, 0, 0, 0, 0] PP=[0, 0, 1, 0, 0, 0] BA1=0 BS=[0,	
Uncertain significance	, 0, 0, 0, 0, 0] PP=[0, 0, 1, 0, 0, 0] BA1=0 BS=[0,	
Uncertain significance	, 0, 0, 0, 0, 0] PP=[0, 0, 0, 0, 0, 0] BA1=0 BS=[0,	
Pathogenic	, 0, 0, 0, 0, 0] PP=[0, 0, 1, 0, 0, 0] BA1=0 BS=[0,	
Pathogenic	, 0, 0, 0, 0, 0] PP=[0, 0, 1, 0, 0, 0] BA1=0 BS=[0,	
Likely pathogenic	, 0, 0, 0, 0, 0] PP=[0, 0, 0, 0, 0, 0] BA1=0 BS=[0,	
Uncertain significance	, 0, 0, 0, 0, 0] PP=[0, 0, 0, 0, 0, 0] BA1=0 BS=[0,	
Uncertain significance	, 0, 0, 0, 0, 0] PP=[0, 0, 0, 0, 0, 0] BA1=0 BS=[0,	

Uncertain significance	, 0, 0, 0, 0, 0] PP=[0, 0, 0, 0, 0, 0] BA1=0 BS=[0,	
Likely pathogenic	, 0, 0, 0, 0, 0] PP=[0, 1, 1, 0, 1, 0] BA1=0 BS=[0,	

MGI	ClinVar	MalaCards	COSMIC	ICGC	GO_BP
e;MP:0005380|embryogenesis phenotype;MP:0		iple, (3); P	OCCUREN		scle cell ap
07|hematopoietic system phenotype;MP:00538		eaver synd			71,histone
07|hematopoietic system phenotype;MP:00538		eaver synd			71,histone
henotype;MP:0005387|immune system phenoty		yndrome,			019827,st
henotype;MP:0005387|immune system phenoty		yndrome,			019827,st
henotype;MP:0005387|immune system phenoty		yndrome,			019827,st
henotype;MP:0005387|immune system phenoty		yndrome,			019827,st
henotype;MP:0005387|immune system phenoty	2,MedGe	yndrome,	OCCUREN		019827,st
henotype;MP:0005387|immune system phenoty		yndrome,			019827,st
henotype;MP:0005387|immune system phenoty		yndrome,	OCCURE		019827,st
henotype;MP:0005387|immune system phenoty		yndrome,	OCCUREN		019827,st
henotype;MP:0005387|immune system phenoty	MIM:Orp	yndrome,			019827,st
henotype;MP:0005387|immune system phenoty		yndrome,			019827,st
henotype;MP:0005387|immune system phenoty		yndrome,			019827,st
henotype;MP:0005387|immune system phenoty		yndrome,			019827,st
henotype;MP:0005387|immune system phenoty	MIM:Orp	yndrome,	OCCUREN		019827,st
henotype;MP:0005387|immune system phenoty		yndrome,			019827,st
henotype;MP:0005387|immune system phenoty		yndrome,			019827,st
henotype;MP:0005387|immune system phenoty	MIM:Orp	yndrome,	OCCUREN		019827,st
henotype;MP:0005387|immune system phenoty		yndrome,			019827,st
henotype;MP:0005387|immune system phenoty		yndrome,			019827,st
henotype;MP:0005387|immune system phenoty	V0000034	yndrome,			019827,st
henotype;MP:0005387|immune system phenoty		yndrome,			019827,st
henotype;MP:0005387|immune system phenoty	CV00039	yndrome,	NCE=1(la		019827,st
henotype;MP:0005387|immune system phenoty		yndrome,			019827,st
henotype;MP:0005387|immune system phenoty		yndrome,			019827,st
henotype;MP:0005387|immune system phenoty		yndrome,			019827,st
henotype;MP:0005387|immune system phenoty		yndrome,			019827,st
henotype;MP:0005387|immune system phenoty		yndrome,			019827,st
henotype;MP:0005387|immune system phenoty		yndrome,			019827,st
henotype;MP:0005387|immune system phenoty		yndrome,			019827,st
henotype;MP:0005387|immune system phenoty		yndrome,			019827,st
henotype;MP:0005387|immune system phenoty		yndrome,			019827,st
henotype;MP:0005387|immune system phenoty		yndrome,			019827,st
henotype;MP:0005387|immune system phenoty		yndrome,			019827,st
0005389|reproductive system phenotype;MP:00		ng (3); Li			GO:00103
0005389|reproductive system phenotype;MP:00		ng (3); Li			GO:00103
0005389|reproductive system phenotype;MP:00		ng (3); Li			GO:00103
0005389|reproductive system phenotype;MP:00		ng (3); Li	9;OCCUF		GO:00103
0005389|reproductive system phenotype;MP:00		ng (3); Li			GO:00103
0005389|reproductive system phenotype;MP:00		ger-Elliso			GO:00103
0003631|nervous system phenotype;MP:00053		docrine ne	CCUREN	DAD-US|1	nsory perc
ing/vestibular/ear phenotype;MP:0005371|limb		ome 17p I			lar organis

ing/vestibular/ear phenotype;MP:0005371|limb		ome 17p I			lar organis
ing/vestibular/ear phenotype;MP:0005371|limb		ome 17p I			lar organis
ing/vestibular/ear phenotype;MP:0005371|limb		ome 17p I			lar organis
7|hematopoietic system phenotype;MP:00053		eaver sync	skin),2(pa	MELA-AU	71,histone

GO_MF	GO_CC	GG_Path	KING SE	KING SE	OLOGY	Case(0|1|2	t Allele F	3086_ger
r activity|(	9,cytosol|(	(human)|	CGATGG	CGCACC	2	129|2|0	0.00763	C/C
1490,chror	asm|GO:0	an)|hsa05	GGAGTA	GACTCTA	1	130|1|0	0.00382	G/G
1490,chror	asm|GO:0	an)|hsa05	TTCAAA	AAACAC	1	123|1|0	0.00403	C/C
or activity	s|GO:0005		ggcggAG	GACAGTA	1	129|1|0	0.00385	
or activity	s|GO:0005		GGCGAG	AGAAGA	1	129|1|0	0.00385	
or activity	s|GO:0005		GCGAGG	CAACAT	1	129|1|0	0.00385	
or activity	s|GO:0005		GGGGGA	AGAAGA	1	129|1|0	0.00385	
or activity	s|GO:0005		AAGATG	GGAGAT	1	129|1|0	0.00385	
or activity	s|GO:0005		GCGTCG	ACGAA	1	128|2|0	0.00769	
or activity	s|GO:0005		CATCCA	GTTCTC	1	129|1|0	0.00385	
or activity	s|GO:0005		GAAGAA	CTGGCC	1	129|1|0	0.00385	
or activity	s|GO:0005		TGTGGT	TGTGAA	1	129|1|0	0.00385	
or activity	s|GO:0005		TGGTGA	CCAACT	1	129|1|0	0.00385	
or activity	s|GO:0005		GGTGAA	GAAGAC	1	127|3|0	0.01154	
or activity	s|GO:0005		ATCTTC	GCATGG	0	129|1|0	0.00385	
or activity	s|GO:0005		AAGAAT	GGCCCA	1	128|2|0	0.00769	
or activity	s|GO:0005		ATGTGA	CAGGGG	1	129|1|0	0.00385	
or activity	s|GO:0005		AGGACT	TTTATT	1	130|1|0	0.00382	
or activity	s|GO:0005		TGGAAA	TTCTAC	1	124|7|0	0.02672	
or activity	s|GO:0005		GGAAAC	TCTACT	1	130|1|0	0.00382	
or activity	s|GO:0005		GCCAAC	AACGTG	1	131|1|0	0.00379	
or activity	s|GO:0005		CTACAC	CCTGTT	1	131|1|0	0.00379	
or activity	s|GO:0005		CACATT	CCGACG	1	131|1|0	0.00379	
or activity	s|GO:0005		CGTGCA	TTCATT	1	131|1|0	0.00379	
or activity	s|GO:0005		CCTTTC	TTTACT	1	131|1|0	0.00379	
or activity	s|GO:0005		GTTTTA	CTTGGA	1	129|2|0	0.00763	
or activity	s|GO:0005		CCTGAT	CTTTTTA	1	130|1|0	0.00382	
or activity	s|GO:0005		GCTCCC	ATATTT	1	128|1|0	0.00388	
or activity	s|GO:0005		TGAAG	CTAAGA	1	131|1|0	0.00379	
or activity	s|GO:0005		AAATTG	AGACTG	1	131|1|0	0.00379	
or activity	s|GO:0005		TTTTGT	AGTATG	1	130|2|0	0.00758	
or activity	s|GO:0005		TTTAGA	GAGAAG	1	131|1|0	0.00379	
or activity	s|GO:0005		TGCACC	TTACTG	1	131|1|0	0.00379	
or activity	s|GO:0005		GAGTAG	TCTATA	1	129|1|0	0.00385	
or activity	s|GO:0005		GGTTGG	AGTAAC	1	131|1|0	0.00379	
istone-lys	reticulum	gulation in	CTTGTC	CCGCCG	1	130|1|0	0.00382	G/G
istone-lys	reticulum	gulation in	GTAGTG	CTAGGG	1	130|1|0	0.00382	C/C
istone-lys	reticulum	gulation in	ACCTCT	ACAAGA	1	130|0|1	0.00763	C/C
istone-lys	reticulum	gulation in	CAGGCC	CAGGCC	1	130|1|0	0.00382	G/G
istone-lys	reticulum	gulation in	GGTGGA	GGAGCT	1	130|1|0	0.00382	CCC/GCA
istone-lys	reticulum	gulation in	ATGACG	AGGAG	1	123|1|0	0.00403	C/C
ming grow	31464,Cul	easles - H	GCCCGA	GCCGGC	1	129|1|0	0.00385	C/C
:0003700,	plasm|GO		CTGCCC	GAGCCC	1	130|1|0	0.00382	G/G

:0003700,‹	)plasm|GO		CTCGAG	GGTGAC	1	130|1|0	0.00382	C/C
:0003700,‹	)plasm|GO		CCCTAC	ACGCGC	1	128|3|0	0.01145	A/A
:0003700,‹	)plasm|GO		CACGCA	GGCCAA	1	124|1|0	0.004	G/G
490,chror	asm|GO:0	an)|hsa05	GGTTAT	TGCACA	1	127|4|0	0.01527	A/A

33086_rea	03571_ge	03571_rea	09670_ge	09670_rea	12276_ge	12276_rea	16492_ge	16492_rea
3170:0	C/C	1550:0	C/C	1814:0	C/C	1790:0	C/C	1698:0
1231:0	G/G	1520:0	G/G	1694:0	G/G	1354:0	G/G	1378:0
28:0	C/C	59:0	C/C	40:0	C/C	81:0	C/C	62:0
168:0	G/G	113:0	G/G	114:0	G/G	130:0	G/G	296:0
434:0	C/C	1162:0	C/C	1159:0	C/C	892:0	C/C	1363:0
434:0	C/C	1162:0	C/C	1159:0	C/C	892:0	C/C	1363:0
462:0	G/G	1051:0	G/G	1228:0	G/G	1017:0	G/G	882:0
55:0	CCC/GCA	223:0	CCC/GCA	238:0	CCC/GCA	120:0	CCC/GCA	66:0
29:0	C/C	150:0	C/C	17:0	C/C	23:0	C/C	288:0
195:0	C/C	566:0	C/C	518:0	C/C	564:0	C/C	222:0
432:0	G/G	379:0	G/G	187:0	G/G	196:0	G/G	370:0

798:0	C/C	441:0	C/C	457:0	C/C	613:0	C/C	531:0
793:0	A/A	433:0	A/A	185:0	A/A	196:0	A/A	527:0
121:0	G/G	1611:0	G/G	1573:0	G/G	836:0	G/G	825:0
55:0	A/A	143:0	A/A	154:0	A/A	280:0	A/A	191:0

17292_ge	17292_rea	17629_ge	17629_rea	37569_ge	37569_rea	51229_ge	51229_rea	61407_ge
C/C	955:0	C/C	1523:0	C/A	5640:5237	C/C	2372:0	C/C
G/G	1796:0	G/G	2995:0	G/G	2568:0	G/G	729:0	G/G
C/C	44:0	C/C	188:0	C/C	141:0	C/C	29:0	C/C
G/G	78:0	G/G	129:0	G/G	128:0	G/G	264:0	G/G
C/C	1444:0	C/C	1078:0	C/C	1011:0	C/C	1449:0	C/C
C/C	1444:0	C/C	1078:0	C/C	1011:0	C/C	1449:0	C/C
G/G	1102:0	G/G	900:0	G/A	287:504	G/G	889:0	G/G
CCC/GCA	228:0	CCC/GCA	138:0	CCC/GCA	149:0	CCC/GCA	115:0	CCC/GCA
C/C	196:0	C/C	319:0	C/C	51:0	C/C	409:0	C/C
C/C	510:0	C/C	852:0	C/C	668:0	C/C	350:0	C/C
G/G	87:0	G/G	155:0	G/G	314:0	G/G	159:0	G/G

C/C	170:0	C/C	321:0	C/C	568:0	C/C	504:0	C/C
A/A	87:0	A/A	155:0	A/A	314:0	A/A	159:0	A/A
G/G	712:0	G/G	431:0	G/G	1374:0	G/G	1124:0	G/G
A/A	159:0	A/A	266:0	A/A	280:0	A/A	98:0	A/A

61407_rea	66570_ge	66570_rea	68355_ge	68355_rea	69417_ge	69417_rea	75980_ge	75980_rea
2159:0	C/C	1605:0	C/C	2139:0	C/C	1871:0	C/C	1816:0
534:0	G/G	462:0	G/G	2424:0	G/G	1099:0	G/G	2308:0
17:0	C/C	13:0	C/C	128:0	C/C	55:0	C/C	84:0
388:0	G/G	133:0	G/G	242:0	G/G	120:0	G/G	132:0
890:0	C/C	88:0	C/C	1168:0	C/C	610:0	C/C	1210:0
890:0	C/C	88:0	C/C	1168:0	C/C	610:0	C/C	1210:0
358:0	G/G	128:0	G/G	1344:0	G/G	620:0	G/G	1327:0
56:0	CCC/GCA	66:0	CCC/GCA	235:0	CCC/GCA	140:0	CCC/GCA	242:0
31:0	C/C	27:0	C/C	209:0	C/C	155:0	C/C	19:0
123:0	C/C	51:0	C/C	645:0	C/C	328:0	C/C	528:0
183:0	G/G	190:0	G/G	417:0	G/G	316:0	G/G	418:0

509:0	C/C	292:0	C/C	527:0	C/C	463:0	C/C	635:0
184:0	A/A	283:0	A/A	417:0	A/A	316:0	A/A	417:0
860:0	G/G	989:0	G/G	798:0	G/G	682:0	G/G	1679:0
59:0	A/A	34:0	A/A	190:0	A/A	167:0	A/A	195:0

88323_ge	88323_rea	90119_ge	90119_rea	93334_ge	93334_rea	95863_ge	95863_rea	22608_ge
C/C	1926:0	C/C	1726:0	C/C	1441:0	C/C	2345:0	C/C
G/G	1354:0	G/G	2465:0	G/G	1350:0	G/G	1465:0	G/G
C/C	66:0	C/C	161:0	C/C	46:0	C/C	56:0	C/C
G/G	471:0	G/G	113:0	G/G	190:0	G/G	217:0	G/G
C/C	1140:0	C/C	838:0	C/C	1194:0	C/C	1232:0	C/C
C/C	1140:0	C/C	838:0	C/C	1194:0	C/C	1232:0	C/C
G/G	636:0	G/G	1130:0	G/G	1033:0	G/G	1075:0	G/G
CCC/GCA	65:0	CAGGCC	104:218	CCC/GCA	141:0	CCC/GCA	160:0	CCC/GCA
C/C	87:0			C/C	172:0	C/C	525:0	C/C
C/C	270:0	C/C	687:0	C/C	412:0	C/C	365:0	C/C
G/G	819:0	G/G	481:0	G/G	244:0	G/G	470:0	G/G

C/C	365:0	C/C	416:0	C/C	309:0	C/C	511:0	C/C
A/A	357:0	A/A	410:0	A/A	302:0	A/A	4495:0	A/A
G/G	799:0	G/G	731:0	G/G	902:0	G/G	866:0	G/G
A/A	221:0	A/A	356:0	A/A	114:0	A/A	125:0	A/A

22608_rea	30936_ge	30936_rea	32054_ge	32054_rea	37816_ge	37816_rea	48291_ge	48291_rea
1133:0	C/C	2127:0	C/C	1964:0	C/C	2236:0	C/C	1591:0
1947:0	G/G	1382:0	G/G	285:0	G/G	1528:0	G/G	264:0
80:0	C/C	30:0	C/C	12:0	C/C	38:0	C/C	11:0
140:0	G/G	59:0	G/G	309:0	G/G	111:0	G/G	269:0
1399:0	C/C	409:0	C/C	370:0	C/C	1246:0	C/C	276:0
1399:0	C/C	409:0	C/C	370:0	C/C	1246:0	C/C	276:0
1269:0	G/G	435:0	G/G	141:0	G/G	810:0	G/G	103:0
220:0	CCC/GCA	192:0	CCC/GCA	252:0	CCC/GCA	230:0	CCC/GCA	227:0
214:0	C/C	29:0	C/C	46:0	C/C	16:0	C/C	261:0
419:0	C/C	324:0	C/C	87:0	C/C	303:0	C/C	48:0
240:0	G/G	221:0	G/G	125:0	G/G	112:0	G/G	73:0

493:0	C/C	332:0	C/C	345:0	C/C	253:0	C/C	253:0
237:0	A/A	328:0	A/A	2106:0	A/A	111:0	A/A	73:0
1150:0	G/G	1412:0	G/G	512:0	G/G	785:0	G/G	541:0
177:0	A/A	51:0	A/A	43:0	A/A	78:0	A/A	20:0

50163_ge	50163_rea	15340_gen	15340_rea	56970_ge	56970_rea	65949_ge	65949_rea	68735_ge
C/C	1082:0	C/C	2210:0	C/C	1648:0	C/C	2239:0	C/C
G/G	2618:0	G/G	3105:0	G/G	3209:0	G/G	2329:0	G/G
C/C	88:0	C/C	202:0	C/C	228:0	C/C	173:0	C/C
G/G	179:0	G/G	192:0	G/G	162:0	G/G	345:0	G/G
C/C	1093:0	C/C	944:0	C/C	959:0	C/C	1224:0	C/C
C/C	1093:0	C/C	944:0	C/C	959:0	C/C	1224:0	C/C
G/G	854:0	G/G	1221:0	G/G	1075:0	G/G	1801:0	G/G
CCC/GCA	116:0	CCC/GCA	257:0	CCC/GCA	115:0	CCC/GCA	227:0	CCC/GCA
C/C	20:0	C/C	21:0	C/C	460:0	C/C	397:0	C/C
C/C	221:0	C/C	156:0	C/C	962:0	C/C	798:0	C/C
G/G	295:0	G/G	206:0	G/G	321:0	G/G	341:0	G/G

C/C	480:0	C/C	354:0	C/C	432:0	C/C	576:0	C/C
A/A	293:0	A/A	205:0	A/A	320:0	A/A	7931:0	A/A
G/G	1199:0	G/G	305:0	G/G	326:0	G/G	76:0	G/G
A/A	181:0	A/A	293:0	A/A	281:0	A/A	329:0	A/A

68735_rea	71276_ge	71276_rea	73704_ge	73704_rea	26048_ger	26048_rea	47204_ger	47204_rea
2041:0	C/C	1899:0	C/C	1476:0	C/C	894:0	C/C	448:0
1737:0	G/G	3280:0	G/G	1488:0	G/G	520:0	G/G	152:0
31:0	C/C	275:0	C/C	81:0	C/C	30:0	C/C	13:0
184:0	G/A	2593:433	G/G	112:0	G/G	93:0	G/G	167:0
1904:0	C/C	895:0	C/C	1180:0	C/C	487:0	C/C	17:0
1904:0	C/C	895:0	C/C	1180:0	C/C	487:0	C/C	17:0
1522:0	G/G	1118:0	G/G	1134:0	G/G	361:0	G/G	34:0
364:0	CCC/GCA	169:0	CCC/GCA	91:0	CCC/GCA	51:0	CCC/GCA	19:0
14:0	C/A	3:12	C/C	31:0	C/C	12:0	C/C	297:0
537:0	C/C	760:0	C/C	350:0	C/C	187:0	C/C	54:0
151:0	G/G	246:0	G/G	302:0	G/G	45:0	G/G	293:0

316:0	C/C	655:0	C/C	530:0	C/C	283:0	C/C	227:0
151:0	A/A	246:0	A/A	302:0	A/A	280:0	A/G	464:78
544:0	G/G	733:0	G/G	668:0	G/G	462:0	G/G	141:0
73:0	A/A	294:0	A/A	165:0	A/A	92:0	A/A	70:0

52938_gen	52938_rea	53792_gen	53792_rea	54621_gen	54621_rea	55526_gen	55526_rea	60550_gen
C/C	1061:0	C/C	1667:0	C/C	945:0	C/C	1369:0	C/C
G/G	754:0	G/G	269:0	G/G	127:0	G/G	164:0	G/G
C/C	37:0	C/C	10:0	C/C	9:0	C/C	8:0	C/C
G/G	130:0	G/G	389:0	G/G	106:0	G/G	299:0	G/G
C/C	262:0	C/C	176:0	C/C	32:0	C/C	96:0	C/C
C/C	262:0	C/C	176:0	C/C	32:0	C/C	96:0	C/C
G/G	361:0	G/G	124:0	G/G	46:0	G/G	55:0	G/G
CCC/GCA	62:0	CCC/GCA	186:0	CCC/GCA	61:0	CCC/GCA	61:0	CCC/GCA
C/C	142:0	C/C	316:0	C/C	66:0	C/C	75:0	C/C
C/C	197:0	C/C	50:0	C/C	42:0	C/C	53:0	C/C
G/G	228:0	G/G	179:0	G/G	63:0	G/G	531:0	G/G

C/C	267:0	C/C	616:0	C/C	330:0	C/C	760:0	C/C
A/A	267:0	A/A	3752:0	A/A	65:0	A/A	4186:0	A/A
G/G	744:0	G/G	737:0	G/G	450:0	G/G	884:0	G/G
A/A	120:0	A/A	33:0	A/A	34:0	A/A	46:0	A/A

50550_rea	12368_gen	12368_rea	16080_gen	16080_rea	28109_gen	28109_rea	37309_gen	37309_rea
1942:0	C/C	1572:0	C/C	993:0	C/C	1227:0	C/C	1262:0
1313:0	G/G	572:0	G/G	510:0	G/G	1468:0	G/G	631:0
25:0			C/C	10:0	C/C	24:0	C/C	15:0
113:0	G/G	139:0	G/G	101:0	G/G	75:0	G/G	70:0
617:0	C/C	419:0	C/C	442:0	C/C	251:0	C/C	381:0
617:0	C/C	419:0	C/C	442:0	C/C	251:0	C/C	381:0
575:0	G/G	451:0	G/G	317:0	G/G	269:0	G/G	434:0
59:0	CCC/GCA	377:0	CCC/GCA	94:0	CCC/GCA	252:0	CCC/GCA	165:0
29:0	C/C	149:0	C/C	121:0	C/C	10:0	C/C	154:0
204:0	C/C	323:0	C/C	105:0	C/C	258:0	C/C	166:0
482:0	G/G	134:0	G/G	94:0	G/G	135:0	G/G	116:0

622:0	C/C	341:0	C/C	209:0	C/C	350:0	C/C	423:0
481:0	A/A	134:0	A/A	94:0	A/A	135:0	A/A	116:0
1252:0	G/G	1564:0	G/G	843:0	G/G	1412:0	G/G	1594:0
123:0	A/A	49:0	A/A	60:0	A/A	67:0	A/A	63:0

38331_gen	38331_rea	42336_gen	42336_rea	47616_gen	47616_rea	60031_gen	60031_rea	69115_gen
C/C	1374:0	C/C	1405:0	C/C	1767:0	C/C	2570:0	C/C
G/G	1669:0	G/G	457:0	G/G	2329:0	G/G	2794:0	G/G
C/C	9:0	C/C	19:0	C/C	99:0	C/C	88:0	C/C
G/G	1419:0	G/G	258:0	G/G	183:0	G/G	110:0	G/G
C/C	409:0	C/C	586:0	C/C	1157:0	C/C	1565:0	C/C
A/A	15:295	C/C	586:0	C/C	1157:0	C/C	1565:0	C/C
G/G	546:0	G/G	475:0	G/G	1402:0	G/G	1344:0	G/G
CCC/GCA	165:0	CCC/GCA	58:0	CCC/GCA	236:0	CCC/GCA	414:0	CCC/GCA
		C/C	285:0	C/C	18:0	C/C	170:0	
C/C	780:0	C/C	178:0	C/C	854:0	C/C	622:0	C/C
G/G	71:0	G/G	777:0	G/G	300:0	G/G	251:0	G/G

C/C	361:0	C/C	442:0	C/C	507:0	C/C	454:0	C/C
A/A	358:0	A/A	441:0	A/A	300:0	A/A	251:0	A/A
G/G	2136:0	G/G	1773:0	G/G	887:0	G/G	1019:0	G/G
A/A	77:0	A/A	129:0	A/A	181:0	A/A	200:0	A/A

69115_rea	73970_ger	73970_rea	74439_ger	74439_rea	37747_ger	37747_rea	94221_ger	94221_rea
2086:0	C/C	1410:0	C/C	1327:0	C/C	1352:0	C/C	1679:0
1744:0	G/G	465:0	G/G	1303:0	G/G	1524:0	G/G	1385:0
50:0	C/C	25:0	C/C	19:0	C/C	41:0	C/C	56:0
93:0	G/G	282:0	G/G	114:0	G/G	99:0	G/G	100:0
764:0	C/C	672:0	C/C	1110:0	C/C	882:0	C/C	987:0
764:0	C/C	672:0	C/C	1110:0	C/C	882:0	C/C	987:0
627:0	G/G	289:0	G/G	887:0	G/G	779:0	G/G	870:0
104:0	CCC/GCA	88:0	CCC/GCA	120:0	CCC/GCA	183:0	CCC/GCA	231:0
	C/C	52:0	C/C	30:0	C/C	11:0	C/C	144:0
515:0	C/C	145:0	C/-	28:180	C/C	437:0	C/C	439:0
436:0	G/G	159:0	G/G	302:0	G/G	171:0	G/G	465:0

644:0	C/C	479:0	C/C	568:0	C/C	311:0	C/C	422:0
624:0	A/A	159:0	A/A	564:0	A/A	171:0	A/A	417:0
2481:0	G/G	1571:0	G/G	3090:0	G/G	2592:0	G/G	1054:0
172:0	A/A	112:0	A/A	58:0	A/A	104:0	A/A	143:0

939_A3_g	939_A3_r	2776_A3_	2776_A3_	571_A4_1	571_A4_1	9670_A1_	9670_A1_	6492_A4_
G/G	1209:0	G/G	2678:0	G/G	1520:0	G/G	2746:0	G/G
AGC/CGT	1209:0	AGC/CGT	2678:0	AGC/CGT	1520:0	AGC/CGT	2746:0	AGC/CGT
G/G	1209:0	G/G	2678:0	G/G	1520:0	G/G	2746:0	G/G
C/C	1209:0	C/C	2678:0	C/C	1520:0	C/C	2746:0	C/C
C/C	1209:0	C/C	2678:0	C/C	1520:0	C/C	2746:0	C/C
G/G	1209:0	G/G	2678:0	G/G	1520:0	G/G	2746:0	G/G
G/G	1209:0	G/G	2678:0	G/G	1520:0	G/G	2746:0	G/G
G/G	1209:0	G/G	2678:0	G/G	1520:0	G/G	2746:0	G/G
G/G	1209:0	G/G	2678:0	G/G	1520:0	G/G	2746:0	G/G
TTC/GGA	1209:0	TTC/GGA	2678:0	TTC/GGA	1520:0	TTC/GGA	2746:0	TTC/GGA
G/G	1209:0	G/G	2678:0	G/G	1520:0	G/G	2746:0	G/G
ACT/ATG	1209:0	ACT/ATG	2678:0	ACT/ATG	1520:0	ACT/ATG	2746:0	ACT/ATG
G/G	1209:0	G/G	2678:0	G/G	1520:0	G/G	2746:0	G/G
G/G	1209:0	G/G	2678:0	G/G	1520:0	G/G	2746:0	G/C
G/G	1925:0	G/G	2242:0	G/G	3158:0	G/G	2767:0	G/G
C/C	1925:0	C/G	2375:1596	C/C	3158:0	C/C	2767:0	C/C
T/T	1925:0	T/T	2247:0	T/T	3158:0	T/T	2767:0	T/T
-/-	1925:0	-/-	2247:0	-/-	3158:0	-/-	2767:0	-/-
T/T	1925:0	T/T	2247:0	T/T	3158:0	T/T	2767:0	T/T
TGC/ATA	1925:0	TGC/ATA	2247:0	TGC/ATA	3158:0	TGC/ATA	2767:0	TGC/ATA
C/C	1925:0	C/T	1174:935	C/C	3158:0	C/C	2767:0	C/C
G/G	1929:0	G/G	2247:0	G/G	3163:0	G/G	2767:0	G/G
AG/AG	148:0	AG/AG	227:0	AG/AG	65:0	AG/AG	139:0	AG/AG
T/T	148:0	T/T	227:0	T/T	65:0	T/T	139:0	T/T
C/C	647:0	C/C	650:0	C/C	583:0	C/C	934:0	C/C
T/T	1240:0	T/T	1650:0	T/T	1456:0	T/T	1952:0	T/T
G/G	1240:0	G/G	1650:0	G/G	1456:0	G/G	1952:0	G/G
C/C	1240:0	C/C	1650:0	C/C	1456:0	C/C	1952:0	C/C
A/A	152:0	A/A	241:0	A/A	95:0	A/A	173:0	A/A
G/G	152:0	G/G	241:0	G/G	95:0	G/G	173:0	G/G
G/G	727:0	G/G	1109:0	G/G	1051:0	G/G	1600:0	G/G
C/C	2261:0	C/C	2521:0	C/C	3685:0	C/C	3619:0	C/C

492_A4_	92_A3_1	92_A3_1	629_A3_	629_A3_	569_A2_	569_A2_	218_A3_	218_A3_
5001:0	G/G	326:0	G/G	2613:0	G/G	2378:0	G/G	103:0
5001:0	AGC/CGT	326:0	AGC/CGT	2613:0	AGC/CGT	2378:0	AGC/CGT	103:0
5001:0	G/G	326:0	G/G	2613:0	G/G	2378:0	G/G	103:0
5001:0	C/C	326:0	C/C	2613:0	C/C	2378:0	C/C	103:0
5001:0	C/C	326:0	C/C	2613:0	C/C	2378:0	C/C	103:0
5001:0	G/G	326:0	G/G	2613:0	G/G	2378:0	G/G	103:0
5001:0	G/G	326:0	G/G	2613:0	G/G	2378:0	G/G	103:0
5001:0	G/G	326:0	G/G	2613:0	G/G	2378:0	G/G	103:0
5001:0	G/G	326:0	G/G	2613:0	G/G	2378:0	G/G	103:0
5001:0	TTC/GGA	326:0	TTC/GGA	2613:0	TTC/GGA	2378:0	TTC/GGA	103:0
5001:0	G/G	326:0	G/G	2613:0	G/G	2378:0	G/G	103:0
5001:0	ACT/ATG	326:0	ACT/ATG	2613:0	ACT/ATG	2378:0	ACT/ATG	104:0
5001:0	G/G	326:0	G/G	2613:0	G/G	2378:0	G/G	104:0
1254:3089	G/G	326:0	G/G	2613:0	G/G	2378:0	G/G	104:0
3139:0	G/G	3234:0	G/G	2507:0	G/G	2957:0	G/G	1664:0
3139:0	C/C	3234:0	C/C	2507:0	C/C	2957:0	C/C	1664:0
3139:0	T/T	3234:0	T/T	2507:0	T/T	2957:0	T/T	1664:0
3139:0	-/-	3234:0	-/-	2507:0	-/-	2957:0	-/-	1664:0
3139:0	T/T	3234:0	T/T	2507:0	T/T	2957:0	T/T	1664:0
3139:0	TGC/ATA	3234:0	TGC/ATA	2507:0	TGC/ATA	2957:0	TGC/ATA	1664:0
3139:0	C/C	3234:0	C/C	2507:0	C/C	2957:0	C/C	1664:0
3139:0	G/G	3234:0	G/G	2509:0	G/G	2967:0	G/G	1664:0
406:0	AG/AG	142:0	AG/AG	176:0	AG/AG	163:0	AG/AG	204:0
406:0	T/T	142:0	T/T	176:0	T/T	163:0	T/T	204:0
985:0	C/C	448:0	C/C	1154:0	C/C	579:0	C/C	262:0
2523:0	T/T	2460:0	T/T	2277:0	T/T	2297:0	T/T	1368:0
2523:0	G/G	2460:0	G/G	2277:0	G/G	2297:0	G/G	1368:0
2523:0	C/C	2460:0	C/C	2277:0	C/C	2297:0	C/C	1368:0
388:0	A/A	280:0	A/A	202:0	A/A	237:0	A/A	162:0
388:0	G/G	280:0	G/G	202:0	G/G	237:0	G/G	162:0
1528:0	G/G	913:0	G/G	1495:0	G/G	947:0	G/G	485:0
3381:0	C/C	3955:0	C/C	2980:0	C/C	3054:0	C/C	2359:0

1229_A3_	229_A3_	1407_A1_	407_A1_	6570_B3_	6570_B3_	3355_A3_	3355_A3_	9417_A4_
G/G	918:0	G/G	1031:0	G/G	182:0	G/G	2889:0	G/G
AGC/CGT	918:0	AGC/CGT	1031:0	AGC/CGT	182:0	AGC/CGT	2889:0	AGC/CGT
G/G	918:0	G/G	1031:0	G/G	182:0	G/G	2889:0	G/G
C/C	918:0	C/C	1031:0	C/C	182:0	C/C	2889:0	C/C
C/C	918:0	C/C	1031:0	C/C	182:0	C/C	2889:0	C/C
G/G	918:0	G/G	1031:0	G/G	182:0	G/G	2889:0	G/G
G/G	918:0	G/G	1031:0	G/G	182:0	G/G	2889:0	G/G
G/G	918:0	G/G	1031:0	G/G	182:0	G/G	2889:0	G/G
G/G	918:0	G/G	1031:0	G/G	182:0	G/G	2889:0	G/G
TTC/GGA	918:0	TTC/GGA	1031:0	TTC/GGA	182:0	TTC/GGA	2889:0	TTC/GGA
G/G	918:0	G/G	1031:0	G/G	182:0	G/G	2889:0	G/G
ACT/ATG	918:0	ACT/ATG	1031:0	ACT/ATG	182:0	ACT/ATG	2889:0	ACT/ATG
G/G	918:0	G/G	1031:0	G/G	182:0	G/G	2889:0	G/G
G/G	918:0	G/G	1031:0	G/G	182:0	G/G	2889:0	G/G
G/G	2565:0	G/G	2143:0	G/G	4195:0	G/G	1634:0	G/G
C/C	2565:0	C/C	2143:0	C/C	4195:0	C/C	1634:0	C/C
T/T	2565:0	T/T	2143:0	T/T	4195:0	T/T	1634:0	T/A
-/-	2565:0	-/-	2143:0	-/-	4195:0	-/-	1634:0	-/-
T/T	2565:0	T/T	2143:0	T/T	4195:0	T/T	1634:0	T/T
TGC/ATA	2565:0	TGC/ATA	2143:0	TGC/ATA	4195:0	TGC/ATA	1634:0	TGC/ATA
C/C	2565:0	C/C	2143:0	C/C	4195:0	C/C	1634:0	C/C
G/G	2572:0	G/G	2143:0	G/G	4207:0	G/G	1634:0	G/G
AG/AG	178:0	AG/AG	363:0	AG/AG	198:0	AG/AG	440:0	AG/AG
T/T	178:0	T/T	363:0	T/T	198:0	T/T	440:0	T/T
C/C	576:0	C/C	656:0	C/C	543:0	C/C	553:0	
T/T	1701:0	T/T	1895:0	T/T	2439:0	T/T	1209:0	T/T
G/G	1701:0	G/G	1895:0	G/G	2439:0	G/G	1209:0	G/G
C/C	1701:0	C/C	1895:0	C/C	2439:0	C/C	1209:0	C/C
A/A	216:0	A/A	270:0	A/A	245:0	A/A	317:0	A/A
G/G	216:0	G/G	270:0	G/G	245:0	G/G	317:0	G/G
G/G	1069:0	G/G	817:0	G/G	1134:0	G/G	552:0	G/G
C/C	2984:0	C/C	2542:0	C/C	4435:0	C/C	2005:0	C/C

417_A4_	980_A3_	980_A3_	323_A5_	323_A5_	119_A3_	119_A3_	863_A2_	863_A2_
4073:0	G/G	3398:0	G/G	1552:0	G/G	2663:0	G/G	2042:0
4073:0	AGC/CGT	3398:0	AGC/CGT	1552:0	AGC/CGT	2663:0	AGC/CGT	2042:0
4073:0	G/G	3398:0	G/G	1552:0	G/G	2663:0	G/G	2042:0
4073:0	C/C	3398:0	C/C	1552:0	C/C	2663:0	C/C	2042:0
4073:0	C/C	3398:0	C/C	1552:0	C/C	2663:0	C/C	2042:0
4073:0	G/G	3398:0	G/G	1552:0	G/G	2663:0	G/G	2042:0
4073:0	G/G	3398:0	G/G	1552:0	G/G	2663:0	G/G	2042:0
4073:0	G/G	3398:0	G/G	1552:0	G/G	2663:0	G/G	2042:0
4073:0	G/G	3398:0	G/G	1552:0	G/G	2663:0	G/G	2042:0
4073:0	TTC/GGA	3398:0	TTC/GGA	1552:0	TTC/GGA	2663:0	TTC/GGA	2042:0
4073:0	G/G	3398:0	G/G	1552:0	G/G	2663:0	G/G	2042:0
4073:0	ACT/ATG	3398:0	ACT/ATG	1552:0	ACT/ATG	2663:0	ACT/ATG	2042:0
4073:0	G/G	3398:0	G/G	1552:0	G/G	2663:0	G/G	2042:0
4073:0	G/G	3398:0	G/G	1552:0	G/G	2663:0	G/G	2042:0
4343:0	G/G	2142:0	G/G	2757:0	G/G	1894:0	G/G	2638:0
4343:0	C/C	2142:0	C/C	2757:0	C/C	1894:0	C/C	2638:0
1508:2996	T/T	2142:0	T/T	2757:0	T/T	1894:0	T/T	2638:0
4358:0	-/-	2142:0	-/-	2757:0	-/-	1894:0	-/-	2638:0
4358:0	T/T	2142:0	T/T	2757:0	T/T	1894:0	T/T	2638:0
4358:0	TGC/ATA	2142:0	TGC/ATA	2757:0	TGC/ATA	1894:0	TGC/ATA	2638:0
4358:0	C/C	2142:0	C/C	2757:0	C/C	1894:0	C/C	2638:0
4358:0	G/G	2146:0	G/G	2762:0	G/G	1894:0	G/G	2638:0
634:0	AG/AG	103:0	AG/AG	271:0	AG/AG	288:0	AG/AG	237:0
634:0	T/T	103:0	T/T	271:0	T/T	288:0	T/T	237:0
	C/C	714:0	C/C	620:0	C/C	562:0	C/C	609:0
3259:0	T/T	1319:0	T/T	1353:0	T/T	1691:0	T/T	2280:0
3259:0	G/G	1319:0	G/G	1353:0	G/G	1691:0	G/G	2280:0
3259:0	C/C	1319:0	C/C	1353:0	C/C	1691:0	C/C	2280:0
626:0	A/A	108:0	A/A	238:0	A/A	260:0	A/A	272:0
626:0	G/G	108:0	G/G	238:0	G/G	260:0	G/G	272:0
6:0	G/G	1100:0	G/G	1208:0	G/G	844:0	G/G	1017:0
4168:0	C/C	2593:0	C/C	2770:0	C/C	2133:0	C/C	3054:0

2608_A2_	608_A2_	936_A3_	936_A3_	_18B072	_18B072	816_A1_	816_A1_	291_A4_
G/G	9113:0	G/G	1957:0	G/G	2721:0	G/G	913:0	G/G
AGC/CGT	9113:0	AGC/CGT	1957:0	AGC/CGT	2721:0	AGC/CGT	913:0	AGC/CGT
G/G	9113:0	G/G	1957:0	G/G	2721:0	G/G	913:0	G/G
C/C	9113:0	C/C	1957:0	C/C	2721:0	C/C	913:0	C/C
C/C	9113:0	C/C	1957:0	C/C	2721:0	C/C	913:0	C/C
G/G	9113:0	G/G	1957:0	G/G	2721:0	G/G	913:0	G/G
G/G	9113:0	G/G	1957:0	G/G	2721:0	G/G	913:0	G/G
G/G	9113:0	G/G	1957:0	G/G	2721:0	G/G	913:0	G/G
G/G	9113:0	G/G	1957:0	G/G	2721:0	G/G	913:0	G/G
TTC/GGA	9113:0	TTC/GGA	1957:0	TTC/GGA	2721:0	TTC/GGA	913:0	TTC/GGA
G/G	9113:0	G/G	1957:0	G/G	2721:0	G/G	913:0	G/G
ACT/ATG	9113:0	ACT/ATG	1957:0	ACT/ATG	2721:0	ACT/ATG	913:0	ACT/ATG
G/G	9113:0	G/G	1957:0	G/G	2721:0	G/G	913:0	G/G
G/G	9113:0	G/G	1957:0	G/G	2721:0	G/G	913:0	G/G
G/G	4440:0	G/G	2748:0	G/G	3110:0	G/G	3516:0	G/G
C/C	4440:0	C/C	2748:0	C/C	3110:0	C/C	3516:0	C/C
T/T	4440:0	T/T	2748:0	T/T	3110:0	T/T	3516:0	T/T
-/-	4440:0	-/-	2748:0	-/-	3110:0	-/-	3516:0	-/-
T/T	4440:0	T/T	2748:0	T/T	3110:0	T/T	3516:0	T/T
TGC/ATA	4440:0	TGC/ATA	2748:0	TGC/ATA	3110:0	TGC/ATA	3516:0	TGC/ATA
C/C	4440:0	C/C	2748:0	C/C	3110:0	C/C	3516:0	C/C
G/G	4446:0	G/G	2748:0	G/G	3110:0	G/G	3516:0	G/G
AG/AG	474:0	AG/AG	263:0	AG/AG	362:0	AG/AG	245:0	AG/AG
T/T	474:0	T/T	263:0	T/T	362:0	T/T	245:0	T/T
C/C	106:0	C/C	810:0	C/C	927:0	C/C	748:0	C/C
T/T	3070:0	T/T	1644:0	T/T	1788:0	T/T	2964:0	T/T
G/G	3070:0	G/G	1644:0	G/G	1788:0	G/G	2964:0	G/G
C/C	3070:0	C/C	1644:0	C/C	1788:0	C/C	2964:0	C/C
A/A	462:0	A/A	290:0	A/A	407:0	A/A	267:0	A/A
G/G	462:0	G/G	290:0	G/G	407:0	G/G	267:0	G/G
G/G	258:0	G/G	1009:0	G/G	1590:0	G/G	1370:0	G/G
C/C	4415:0	C/C	3395:0	C/C	4352:0	C/C	4365:0	C/C

3291_A4_	0163_A3_	0163_A3_	5340_A1_	5340_A1_	6970_A2_	6970_A2_	5949_A3_	5949_A3_
733:0	G/G	2804:0	G/G	9478:0	G/G	3592:0	G/G	4976:0
733:0	AGC/CGT	2804:0	AGC/CGT	9478:0	AGC/CGT	3592:0	AGC/CGT	4976:0
733:0	G/G	2804:0	G/G	9478:0	G/G	3592:0	G/G	4976:0
733:0	C/C	2804:0	C/C	9478:0	C/C	3592:0	C/C	4976:0
733:0	C/C	2804:0	C/C	9478:0	C/C	3592:0	C/C	4976:0
733:0	G/G	2804:0	G/G	9478:0	G/G	3592:0	G/G	4976:0
733:0	G/G	2804:0	G/G	9478:0	G/G	3592:0	G/G	4976:0
733:0	G/G	2804:0	G/G	9478:0	G/G	3592:0	G/G	4976:0
733:0	G/G	2804:0	G/G	9478:0	G/G	3592:0	G/G	4976:0
733:0	TTC/GGA	2804:0	TTC/GGA	9478:0	TTC/GGA	3592:0	TTC/GGA	4976:0
733:0	G/G	2804:0	G/G	9478:0	G/G	3592:0	G/G	4976:0
733:0	ACT/ATG	2804:0	ACT/ATG	9478:0	ACT/ATG	3592:0	ACT/ATG	4976:0
733:0	G/G	2804:0	G/G	9478:0	G/G	3592:0	G/G	4976:0
733:0	G/G	2804:0	G/G	9478:0	G/G	3592:0	G/G	4976:0
1516:0	G/G	2222:0	G/G	3471:0	G/G	1734:0	G/G	2486:0
1516:0	C/C	2222:0	C/C	3471:0	C/C	1734:0	C/C	2486:0
1516:0	T/T	2222:0	T/T	3471:0	T/T	1734:0	T/T	2486:0
1516:0	-/-	2222:0	-/-	3471:0	-/-	1734:0	-/-	2486:0
1516:0	T/T	2222:0	T/T	3471:0	T/T	1734:0	T/T	2486:0
1516:0	TGC/ATA	2222:0	TGC/ATA	3471:0	TGC/ATA	1734:0	TGC/ATA	2486:0
1516:0	C/C	2222:0	C/C	3471:0	C/C	1734:0	C/C	2486:0
1516:0	G/G	2222:0	G/G	3471:0	G/G	1737:0	G/G	2486:0
125:0	AG/AG	172:0	AG/AG	202:0	AG/AG	230:0	AG/AG	103:0
125:0	T/T	172:0	T/T	202:0	T/T	230:0	T/T	103:0
384:0	C/C	669:0	C/C	1314:0	C/C	641:0	C/C	1217:0
1114:0	T/T	1552:0	T/T	2634:0	T/T	1162:0	T/T	1936:0
1114:0	G/G	1552:0	G/G	2634:0	G/G	1162:0	G/G	1936:0
1114:0	C/C	1552:0	C/C	2634:0	C/C	1162:0	C/C	1936:0
142:0	A/A	239:0	A/A	260:0	A/A	201:0	A/A	129:0
142:0	G/G	239:0	G/G	260:0	G/G	201:0	G/G	129:0
794:0	G/G	1134:0	G/G	2138:0	G/G	891:0	G/G	1917:0
2251:0	C/C	3394:0	C/C	4641:0	C/C	1972:0	C/C	3676:0

3735_A2_	3735_A2_	1276_A2_	276_A2_	3704_A2_	3704_A2_	048_A1_g	048_A1_r	204_A1_g
G/G	10:0	G/G	3681:0	G/G	11967:0	G/G	1554:0	G/G
AGC/CGT	10:0	AGC/CGT	3681:0	AGC/CGT	11967:0	AGC/CGT	1554:0	AGC/CGT
G/G	10:0	G/G	3681:0	G/G	11967:0	G/G	1554:0	G/G
C/C	10:0	C/C	3681:0	C/C	11967:0	C/C	1554:0	C/C
C/C	10:0	C/C	3681:0	C/C	11967:0	C/C	1554:0	C/C
G/G	20:0	G/G	3681:0	G/G	11967:0	G/G	1554:0	G/G
G/G	10:0	G/G	3681:0	G/G	11967:0	G/G	1554:0	G/G
G/G	10:0	G/G	3681:0	G/G	11967:0	G/G	1554:0	G/G
G/G	10:0	G/G	3681:0	G/G	11967:0	G/G	1554:0	G/G
TTC/GGA	10:0	TTC/GGA	3681:0	TTC/GGA	11967:0	TTC/GGA	1554:0	TTC/GGA
G/G	10:0	G/G	3681:0	G/G	11967:0	G/G	1554:0	G/G
ACT/ATG	10:0	ACT/ATG	3681:0	ACT/ATG	11967:0	ACT/ATG	1554:0	ACT/ATG
G/G	10:0	G/G	3681:0	G/G	11967:0	G/G	1556:0	G/G
G/G	10:0	G/G	3681:0	G/G	11967:0	G/G	1556:0	G/G
G/G	3355:0	G/G	2159:0	G/G	3508:0	G/G	2631:0	G/G
C/C	3355:0	C/C	2159:0	C/C	3508:0	C/C	2631:0	C/C
T/T	3355:0	T/T	2159:0	T/T	3508:0	T/T	2631:0	T/T
-/-	3355:0	-/-	2159:0	-/-	3508:0	-/-	2631:0	-/-
T/T	3355:0	T/T	2159:0	T/T	3508:0	T/T	2631:0	T/T
TGC/ATA	3355:0	TGC/ATA	2159:0	TGC/ATA	3508:0	TGC/ATA	2631:0	TGC/ATA
C/C	3355:0	C/C	2159:0	C/C	3508:0	C/C	2631:0	C/C
G/G	3355:0	G/G	2159:0	G/G	3508:0	G/G	2631:0	G/G
AG/AG	43:0	AG/AG	299:0	AG/AG	623:0	AG/AG	226:0	AG/AG
T/T	43:0	T/T	299:0	T/T	623:0	T/T	226:0	T/T
C/C	275:0	C/C	819:0	C/C	1469:0	C/C	1026:0	C/C
T/T	1871:0	T/T	1323:0	T/T	2227:0	T/T	2179:0	T/T
G/G	1871:0	G/G	1323:0	G/G	2227:0	G/G	2179:0	G/G
C/C	1871:0	C/C	1323:0	C/C	2227:0	C/C	2179:0	C/C
A/A	99:0	A/A	291:0	A/A	573:0	A/A	235:0	A/A
G/G	99:0	G/G	291:0	G/G	573:0	G/G	235:0	G/G
G/G	595:0	G/G	1322:0	G/G	1621:0	G/G	1274:0	G/G
C/C	4533:0	C/C	2230:0	C/C	4852:0	C/C	3427:0	C/C

204_A1_r	792_A1_g	792_A1_r	621_A1_g	621_A1_r	526_A1_g	526_A1_r	550_A1_g	550_A1_r
2423:0	G/G	306:0	G/G	375:0	G/G	199:0	G/G	2163:0
2423:0	AGC/CGT	306:0	AGC/CGT	375:0	AGC/CGT	199:0	AGC/CGT	2163:0
2423:0	G/G	306:0	G/G	375:0	G/G	199:0	G/G	2163:0
2423:0	C/C	306:0	C/C	375:0	C/C	199:0	C/C	2163:0
2423:0	C/C	306:0	C/C	375:0	C/C	199:0	C/C	2163:0
2423:0	G/G	306:0	G/G	375:0	G/G	199:0	G/G	2163:0
2423:0	G/G	306:0	G/G	375:0	G/G	199:0	G/T	503:1375
2423:0	G/G	306:0	G/G	375:0	G/G	198:0	G/G	2155:0
2423:0	G/G	306:0	G/G	375:0	G/G	198:0	G/G	2155:0
2423:0	TTC/GGA	306:0	TTC/GGA	375:0	TTC/GGA	198:0	TTC/GGA	2155:0
2423:0	G/G	306:0	G/G	375:0	G/G	198:0	G/G	2155:0
2423:0	ACT/ATG	306:0	ACT/ATG	375:0	ACT/ATG	198:0	ACT/ATG	2155:0
2423:0	G/G	306:0	G/G	378:0	G/G	198:0	G/G	2155:0
2423:0	G/G	306:0	G/G	378:0	G/G	198:0	G/G	2155:0
3119:0	G/G	2285:0	G/G	3045:0	G/G	1495:0	G/G	1867:0
3119:0	C/C	2285:0	C/C	3045:0	C/C	1495:0	C/C	1867:0
3119:0	T/T	2285:0	T/T	3045:0	T/T	1495:0	T/T	1867:0
3119:0	-/-	2285:0	-/-	3045:0	-/-	1495:0	-/-	1867:0
3119:0	T/T	2285:0	T/T	3045:0	T/T	1495:0	T/T	1867:0
3119:0	TGC/ATA	2285:0	TGC/ATA	3045:0	TGC/ATA	1495:0	TGC/ATA	1867:0
3119:0	C/C	2285:0	C/C	3045:0	C/C	1495:0	C/C	1867:0
3119:0	G/G	2288:0	G/G	3045:0	G/G	1495:0	G/G	1867:0
616:0	AG/AG	60:0	AG/AG	422:0	AG/AG	218:0	AG/AG	190:0
616:0	T/T	60:0	T/T	422:0	T/T	218:0	T/T	190:0
1354:0	C/C	721:0	C/C	889:0	C/C	449:0	C/C	619:0
2270:0	T/T	1392:0	T/T	2567:0	T/T	881:0	T/T	1739:0
2270:0	G/G	1392:0	G/G	2567:0	G/G	881:0	G/G	1739:0
2270:0	C/C	1392:0	C/C	2567:0	C/C	881:0	C/C	1739:0
506:0	A/A	101:0	A/A	487:0	A/A	245:0	A/A	194:0
506:0	G/G	101:0	G/G	487:0	G/G	245:0	G/G	194:0
1689:0	G/G	835:0	G/G	637:0	G/G	457:0	G/G	1431:0
3468:0	C/C	2552:0	C/C	4181:0	C/C	1401:0	C/C	3544:0

368_A2_g	368_A2_r	080_A1_g	080_A1_r	109_A2_g	109_A2_r	309_A5_g	309_A5_r	331_A4_g
		G/G	1008:0	G/G	649:0	G/G	292:0	G/G
		AGC/CGT	1008:0	AGC/CGT	649:0	AGC/CGT	292:0	AGC/CGT
		G/G	1008:0	G/G	649:0	G/G	292:0	G/G
		C/C	1008:0	C/C	649:0	C/C	292:0	C/C
		C/C	1008:0	C/C	649:0	C/C	292:0	C/C
		G/G	1008:0	G/G	649:0	G/G	292:0	G/G
		G/G	1008:0	G/G	649:0	G/G	292:0	G/G
		G/G	1008:0	G/G	649:0	G/G	292:0	G/G
		G/G	1008:0	G/G	649:0	G/G	292:0	G/G
		TTC/GGA	1008:0	TTC/GGA	649:0	TTC/GGA	292:0	TTC/GGA
		G/G	1008:0	G/G	649:0	G/G	292:0	G/G
		ACT/ATG	1008:0	ACT/ATG	649:0	ACT/ATG	292:0	ACT/ATG
		G/G	1008:0	G/G	649:0	G/G	292:0	G/G
		G/G	1008:0	G/G	649:0	G/G	292:0	G/G
G/G	2625:0	G/G	2279:0	G/G	3714:0	G/G	2344:0	G/G
C/C	2625:0	C/C	2279:0	C/C	3714:0	C/C	2344:0	C/C
T/T	2625:0	T/T	2279:0	T/T	3714:0	T/T	2344:0	T/T
-/-	2625:0	-/-	2279:0	-/-	3714:0	-/-	2344:0	-/-
T/T	2625:0	T/T	2279:0	T/T	3714:0	T/T	2344:0	T/T
TGC/ATA	2625:0	TGC/ATA	2279:0	TGC/ATA	3714:0	TGC/ATA	2344:0	TGC/ATA
C/C	2625:0	C/C	2279:0	C/C	3714:0	C/C	2344:0	C/C
G/G	2625:0	G/G	2285:0	G/G	3721:0	G/G	2344:0	G/G
AG/AG	75:0	AG/AG	146:0	AG/AG	308:0	AG/AG	67:0	AG/AG
T/T	75:0	T/T	146:0	T/T	308:0	T/T	67:0	T/T
C/C	58:0	C/C	920:0	C/C	605:0	C/C	541:0	C/C
T/T	1949:0	T/T	1478:0	T/T	2932:0	T/T	1984:0	T/T
G/G	1949:0	G/G	1478:0	G/G	2932:0	G/G	1984:0	G/G
C/C	1949:0	C/C	1478:0	C/C	2932:0	C/C	1984:0	C/C
A/A	202:0	A/A	180:0	A/A	462:0	A/A	135:0	A/A
G/G	202:0	G/G	180:0	G/G	462:0	G/G	135:0	G/G
G/G	52:0	G/G	1241:0	G/G	939:0	G/G	574:0	G/G
C/C	2843:0	C/C	2546:0	C/C	4146:0	C/C	3214:0	C/C

331_A4_r	336_A1_g	336_A1_r	616_B1_g	616_B1_r	031_A2_g	031_A2_r	115_A3_g	115_A3_r
118:0	G/G	2415:0	G/G	2344:0	G/G	7897:0	G/G	1389:0
118:0	AGC/CGT	2415:0	AGC/CGT	2344:0	AGC/CGT	7897:0	AGC/CGT	1389:0
118:0	G/G	2415:0	G/G	2344:0	G/G	7897:0	G/G	1389:0
118:0	C/C	2415:0	C/C	2344:0	C/C	7897:0	C/C	1389:0
118:0	C/C	2415:0	C/C	2344:0	C/C	7897:0	C/C	1389:0
118:0	G/G	2415:0	G/G	2344:0	G/G	7897:0	G/G	1389:0
118:0	G/G	2415:0	G/G	2344:0	G/G	7897:0	G/G	1389:0
118:0	G/G	2415:0	G/G	2344:0	G/G	7897:0	G/G	1389:0
118:0	G/G	2415:0	G/G	2344:0	G/G	7897:0	G/G	1389:0
118:0	TTC/GGA	2415:0	TTC/GGA	2344:0	TTC/GGA	7897:0	TTC/GGA	1389:0
118:0	G/G	2415:0	G/G	2344:0	G/G	7897:0	G/G	1389:0
118:0	ACT/ATG	2415:0	ACT/ATG	2344:0	ACT/ATG	7897:0	ACT/ATG	1389:0
118:0	G/G	2415:0	G/G	2344:0	G/G	7897:0	G/G	1389:0
118:0	G/G	2415:0	G/G	2344:0	G/G	7897:0	G/G	1389:0
1320:0	G/G	2431:0	G/G	2711:0	G/G	2562:0	G/G	2083:0
1320:0	C/C	2431:0	C/C	2711:0	C/C	2562:0	C/C	2083:0
1320:0	T/T	2431:0	T/T	2711:0	T/T	2562:0	T/T	2083:0
1320:0	-/-	2431:0	-/-	2711:0	-/-	2562:0	-/-	2083:0
1320:0	T/T	2431:0	T/T	2711:0	T/T	2562:0	T/T	2083:0
1320:0	TGC/ATA	2431:0	TGC/ATA	2711:0	TGC/ATA	2562:0	TGC/ATA	2083:0
1320:0	C/C	2431:0	C/C	2711:0	C/C	2562:0	C/C	2083:0
1320:0	G/G	2439:0	G/G	2711:0	G/G	2562:0	G/G	2083:0
20:0	AG/AG	150:0	AG/AG	204:0	AG/AG	217:0	AG/AG	103:0
20:0	T/T	150:0	T/T	204:0	T/T	217:0	T/T	103:0
391:0	C/C	805:0	C/C	683:0	C/C	721:0	C/C	675:0
917:0	T/T	2040:0	T/T	1487:0	T/T	2445:0	T/T	1362:0
917:0	G/G	2040:0	G/G	1487:0	G/G	2445:0	G/G	1362:0
917:0	C/C	2040:0	C/C	1487:0	C/C	2445:0	C/C	1362:0
61:0	A/A	179:0	A/A	255:0	A/A	263:0	A/A	148:0
61:0	G/G	179:0	G/G	255:0	G/G	263:0	G/G	148:0
779:0	G/G	1128:0	G/G	1120:0	G/G	1123:0	G/G	1076:0
1421:0	C/C	2568:0	C/C	2831:0	C/C	3432:0	C/C	3039:0

70_A3_1_	70_A3_1_	39_A6_1_	39_A6_1_	747_A5_g	747_A5_r	4221_1_ge	4221_1_re	208_A2_g
G/G	251:0	G/G	48:0	G/G	1619:0	G/G	138:0	G/G
AGC/CGT	251:0	AGC/CGT	48:0	AGC/CGT	1619:0	AGC/CGT	138:0	TGCTTAC
G/G	251:0	G/G	48:0	G/G	1619:0	G/G	138:0	G/G
C/C	251:0	C/C	48:0	C/C	1619:0	C/C	138:0	C/C
C/C	251:0	C/C	48:0	C/C	1619:0	C/C	138:0	C/C
G/G	251:0	G/G	48:0	G/G	1619:0	G/G	138:0	G/G
G/G	251:0	G/G	49:0	G/G	1619:0	G/G	138:0	G/G
G/G	251:0	G/G	48:0	G/G	1619:0	G/G	138:0	G/G
G/G	251:0	G/G	48:0	G/G	1619:0	G/G	138:0	G/G
TTC/GGA	251:0	TTC/GGA	48:0	TTC/GGA	1619:0	TTC/GGA	138:0	TTC/GGA
G/G	251:0	G/G	48:0	G/G	1619:0	G/G	138:0	G/G
ACT/ATG	251:0	ACT/ATG	48:0	ACT/ATG	1619:0	ACT/ATG	138:0	ACT/ATG
G/G	251:0	G/G	48:0	G/G	1619:0	G/G	138:0	G/G
G/G	251:0	G/G	48:0	G/G	1619:0	G/G	138:0	G/G
G/G	3052:0	G/G	1893:0	G/G	2808:0	G/G	2506:0	G/G
C/C	3052:0	C/C	1893:0	C/C	2808:0	C/C	2506:0	C/C
T/T	3052:0	T/T	1893:0	T/T	2808:0	T/T	2506:0	T/T
-/-	3052:0	-/-	1893:0	-/-	2808:0	-/-	2506:0	-/-
T/T	3052:0	T/T	1893:0	T/T	2808:0	T/T	2506:0	T/T
TGC/ATA	3052:0	TGC/ATA	1893:0	TGC/ATA	2808:0	TGC/ATA	2506:0	TGC/ATA
C/C	3052:0	C/C	1893:0	C/C	2808:0	C/C	2506:0	C/C
G/G	3052:0	G/G	1893:0	G/G	2808:0	G/G	2506:0	G/G
AG/AG	417:0	AG/AG	138:0	AG/AG	196:0	AG/AG	49:0	AG/AG
T/T	417:0	T/T	138:0	T/T	196:0	T/T	49:0	T/T
C/C	309:0	C/C	226:0	C/C	411:0	C/C	472:0	C/C
T/T	2244:0	T/T	1395:0	T/T	2316:0	T/T	1031:0	T/T
G/G	2244:0	G/G	1395:0	G/G	2316:0	G/G	1031:0	G/-
C/C	2244:0	C/C	1395:0	C/C	2316:0	C/C	1031:0	C/C
A/A	290:0	A/A	149:0	A/A	264:0	A/A	95:0	A/A
G/G	290:0	G/G	149:0	G/G	264:0	G/G	95:0	G/G
G/G	767:0	G/G	394:0	G/G	797:0	G/G	727:0	G/G
C/C	3468:0	C/C	2533:0	C/C	3312:0	C/C	3105:0	C/C

208_A2_r	0253_A3_	0253_A3_r	2151_A1_	151_A1_r	080_A2_g	080_A2_r	_18B0723	_18B0723
1063:0	G/G	6088:0	G/G	7506:0	G/G	1399:0	G/G	869:0
553:369	AGC/CGT	6088:0	AGC/CGT	7506:0	AGC/CGT	1399:0	AGC/CGT	869:0
553:0	G/G	6088:0	G/G	7506:0	G/G	1399:0	G/G	869:0
553:0	C/C	6088:0	C/C	7506:0	C/C	1399:0	C/C	869:0
1061:0	C/C	6088:0	C/C	7506:0	C/C	1399:0	C/C	869:0
1061:0	G/G	6088:0	G/G	7506:0	G/G	1399:0	G/G	869:0
1061:0	G/G	6088:0	G/G	7506:0	G/G	1399:0	G/G	869:0
1061:0	G/G	6088:0	G/G	7506:0	G/G	1399:0	G/G	869:0
1061:0	G/G	6088:0	G/G	7506:0	G/G	1399:0	G/G	869:0
1061:0	TTC/GGA	6088:0	TTC/GGA	7506:0	TTC/GGA	1399:0	TTC/GGA	869:0
1061:0	G/G	6088:0	G/G	7506:0	G/G	1399:0	G/G	869:0
1061:0	ACT/ATG	6088:0	ACT/ATG	7506:0	ACT/ATG	1399:0	ACT/ATG	869:0
1061:0	G/G	6088:0	G/G	7506:0	G/G	1399:0	G/G	869:0
1061:0	G/G	6088:0	G/G	7506:0	G/G	17:0	G/G	869:0
2733:0	G/G	4422:0	G/G	7238:0	G/G	3453:0	G/G	4544:0
2733:0	C/C	4422:0	C/C	7238:0	C/C	3453:0	C/C	4544:0
2733:0	T/T	4422:0	T/T	7238:0	T/T	3453:0	T/T	4544:0
2733:0	-/-	4422:0	-/-	7238:0	-/-	3453:0	-/-	4544:0
2733:0	T/T	4422:0	T/T	7238:0	T/T	3453:0	T/T	4544:0
2733:0	TGC/ATA	4422:0	TGC/ATA	7238:0	TGC/ATA	3453:0	TGC/ATA	4544:0
2733:0	C/C	4422:0	C/C	7238:0	C/C	3453:0	C/C	4544:0
2733:0	G/G	4431:0	G/G	7238:0	G/G	3453:0	G/G	4557:0
315:0	AG/AG	501:0	AG/AG	814:0	AG/AG	245:0	AG/AG	242:0
315:0	T/T	501:0	T/T	814:0	T/T	245:0	T/T	242:0
780:0	C/C	11:0	C/C	9:0	C/C	1040:0	C/C	1136:0
2911:0	T/T	2687:0	T/T	5995:0	T/T	1705:0	T/T	2122:0
1120:1592	G/G	2687:0	G/G	5995:0	G/G	1705:0	G/G	2122:0
1789:0	C/C	2687:0	C/C	5995:0	C/C	1705:0	C/C	2122:0
409:0	A/A	579:0	A/A	909:0	A/A	263:0	A/A	280:0
409:0	G/G	579:0	G/G	909:0	G/G	263:0	G/G	280:0
944:0	G/G	39:0	G/G	33:0	G/G	1360:0	G/G	1197:0
2854:0	C/C	3998:0	C/C	8366:0	C/C	3935:0	C/C	3837:0

167_A2_g	167_A2_r	537_A1_g	537_A1_r	137_A1_g	137_A1_r	715_A1_g	715_A1_r	5992_2_ge
G/G	4600:0	G/G	58:0	G/G	301:0	G/G	8:0	G/G
AGC/CGT	4600:0	AGC/CGT	58:0	AGC/CGT	301:0	AGC/CGT	8:0	AGC/CGT
G/G	4600:0	G/G	58:0	G/G	301:0	G/G	8:0	G/G
C/C	4600:0	C/C	58:0	C/C	301:0	C/C	8:0	C/C
C/C	4600:0	C/C	58:0	C/C	301:0	C/C	8:0	C/C
G/G	4600:0	G/G	58:0	G/G	301:0	G/G	15:0	G/G
G/G	4600:0	G/G	58:0	G/G	301:0	G/G	8:0	G/G
G/G	4600:0	G/G	58:0	G/G	301:0	G/G	8:0	G/G
G/G	4600:0	G/G	58:0	G/G	301:0	G/G	8:0	G/G
TTC/GGA	4600:0	TTC/GGA	58:0	TTC/GGA	301:0	TTC/GGA	8:0	TTC/GGA
G/T	1778:2139	G/G	58:0	G/G	301:0	G/G	8:0	G/G
ACT/ATG	4603:0	ACT/ATG	58:0	ACT/ATG	301:0	ACT/ATG	8:0	ACT/ATG
G/G	4603:0	G/G	58:0	G/G	301:0	G/G	8:0	G/A
G/G	4603:0	G/G	58:0	G/G	301:0	G/G	8:0	G/G
G/G	4541:0	G/G	2052:0	G/G	2970:0	G/G	413:0	G/G
C/C	4541:0	C/C	2052:0	C/C	2970:0	C/C	413:0	C/C
T/T	4541:0	T/T	2052:0	T/T	2970:0	T/T	413:0	T/T
-/-	4541:0	-/-	2052:0	-/-	2970:0	-/-	807:0	-/-
T/T	4541:0	T/T	2052:0	T/T	2970:0	T/T	807:0	T/T
TGC/ATA	4541:0	TGC/ATA	2052:0	TGC/ATA	2970:0	TGC/ATA	807:0	TGC/ATA
C/C	4541:0	C/C	2052:0	C/C	2970:0	C/C	414:0	C/C
G/G	4541:0	G/G	2052:0	G/G	2979:0	G/G	414:0	G/G
AG/AG	576:0	AG/AG	23:0	AG/AG	82:0	AG/AG	116:0	AG/AG
T/T	576:0	T/T	23:0	T/T	82:0	T/T	116:0	T/T
C/C	1486:0	C/C	537:0	C/C	923:0	C/C	115:8	C/T
T/T	3532:0	T/T	1216:0	T/T	2247:0	T/T	450:0	T/T
G/G	3532:0	G/G	1216:0	G/G	2247:0	G/G	450:0	G/G
C/C	3532:0	C/C	1216:0	C/C	2247:0	C/C	450:0	C/C
A/-	758:270	A/A	41:0	A/A	139:0	A/A	96:0	A/A
G/G	533:0	G/G	41:0	G/G	139:0	G/G	96:0	G/G
G/G	1864:0	G/G	839:0	G/G	1280:0	G/G	138:0	G/G
C/C	5302:0	C/C	2950:0	C/C	3261:0	C/C	759:0	C/C

5992_2_re	335_A2_g	335_A2_r	428_A3_g	428_A3_r	066_A5_g	066_A5_r	0110_1_ge	0110_1_re
374:0	G/G	2394:0	G/G	78:0			G/G	958:0
374:0	AGC/CGT	2394:0	AGC/CGT	78:0			AGC/CGT	958:0
374:0	G/G	2394:0	G/G	78:0			G/G	958:0
374:0	C/C	2394:0	C/C	78:0			C/C	958:0
374:0	C/C	2394:0	C/C	78:0			C/C	958:0
374:0	G/G	2394:0	G/G	77:0			G/G	958:0
374:0	G/G	2394:0	G/G	77:0			G/G	958:0
374:0	G/G	2394:0	G/G	77:0			G/G	958:0
374:0	G/G	2394:0	G/G	77:0			G/G	958:0
374:0	TTC/GGA	2394:0	TTC/GGA	77:0			TTC/GGA	958:0
374:0	G/G	2394:0	G/G	77:0			G/G	958:0
374:0	ACT/ATG	2394:0	ACT/ATG	78:0			ACT/ATG	958:0
265:28	G/G	2394:0	G/G	78:0			G/G	958:0
374:0	G/G	2394:0	G/G	78:0			G/G	958:0
1705:0	G/G	1922:0	G/G	935:0	G/G	1512:0	G/G	2187:0
1705:0	C/C	1922:0	C/C	935:0	C/C	1512:0	C/C	2187:0
1705:0	T/T	1922:0	T/T	935:0	T/T	1512:0	T/T	2187:0
1705:0	-/-	1922:0	-/-	935:0	-/-	1512:0	-/-	2187:0
1705:0	T/T	1922:0	T/T	935:0	T/T	1512:0	T/T	2187:0
1705:0	TGC/ATA	1922:0	TGC/ATA	935:0	TGC/ATA	1512:0	TGC/ATA	2187:0
1705:0	C/C	1922:0	C/C	935:0	C/C	1512:0	C/C	2187:0
1705:0	G/G	1922:0	G/G	935:0	G/G	1512:0	G/G	2192:0
354:0	AG/AG	228:0	AG/AG	220:0	AG/AG	17:0	AG/AG	198:0
354:0	T/T	228:0	T/T	220:0	T/T	17:0	T/T	198:0
443:35	C/C	985:0	C/C	170:0			C/C	617:0
1224:0	T/T	1824:0	T/T	798:0	T/T	1422:0	T/T	1561:0
1224:0	G/G	1824:0	G/G	798:0	G/G	1422:0	G/G	1561:0
1224:0	C/C	1824:0	C/C	798:0	C/C	1422:0	C/C	1561:0
283:0	A/A	224:0	A/A	210:0	A/A	54:0	A/A	210:0
283:0	G/G	224:0	G/G	210:0	G/G	28:0	G/G	210:0
814:0	G/G	1089:0	G/G	350:0			G/G	756:0
2855:0	C/C	2414:0	C/C	2513:0	C/C	2875:0	C/C	2378:0

291_A2_g	291_A2_r	68_A3_1_	68_A3_1_	51_A1_1_	51_A1_1_	154_A3_	154_A3_	591_A3_
G/G	6688:0	G/G	96:0	G/G	635:0	G/G	25:0	G/G
AGC/CGT	6688:0	AGC/CGT	96:0	AGC/CGT	635:0	AGC/CGT	25:0	AGC/CGT
G/G	6688:0	G/G	96:0	G/G	635:0	G/G	25:0	G/-
C/C	6688:0	C/C	96:0	C/C	635:0	C/C	25:0	C/C
C/C	6688:0	C/C	96:0	C/C	635:0	C/C	25:0	C/C
G/G	6688:0	G/G	96:0	G/G	635:0	G/G	48:0	G/G
G/G	6688:0	G/G	96:0	G/G	635:0	G/G	25:0	G/G
G/G	6688:0	G/G	96:0	G/G	635:0	G/G	25:0	G/G
G/-	3624:1660	G/G	96:0	G/G	635:0	G/G	25:0	G/G
TTC/GGA	6697:0	TTC/GGA	96:0	TTC/GGA	635:0	TTC/GGA	25:0	TTC/GGA
G/G	6697:0	G/G	96:0	G/G	635:0	G/G	25:0	G/G
ACT/ATG	6697:0	ACT/ATG	96:0	ACT/ATG	635:0	ACT/ATG	25:0	ACT/ATG
G/G	6697:0	G/G	96:0	G/G	635:0	G/G	25:0	G/G
G/G	6697:0	G/G	96:0	G/G	635:0	G/G	25:0	G/G
G/G	3959:0	G/G	2285:0	G/G	1793:0	G/G	826:0	G/G
C/C	3959:0	C/C	2285:0	C/C	1793:0	C/C	826:0	C/C
T/T	3959:0	T/T	2285:0	T/T	1793:0	T/T	826:0	T/T
-/T	2673:4424	-/-	2285:0	-/-	1793:0	-/-	826:0	-/-
T/T	3968:0	T/T	2285:0	T/T	1793:0	T/T	826:0	T/T
TGC/ATA	3968:0	TGC/ATA	2285:0	TGC/ATA	1793:0	TGC/ATA	826:0	TGC/ATA
C/C	3968:0	C/C	2285:0	C/C	1793:0	C/C	826:0	C/C
G/G	3968:0	G/G	2288:0	G/G	1793:0	G/C	204:599	G/G
AG/AG	377:0	AG/AG	17:0	AG/AG	45:0	AG/AG	129:0	AG/AG
T/T	377:0	T/T	17:0	T/T	45:0	T/T	129:0	T/T
C/C	395:0	C/C	1229:0	C/C	139:0	C/C	101:0	C/C
T/T	2300:0	T/T	2139:0	T/T	1046:0	T/T	572:0	T/T
G/G	2300:0	G/G	2139:0	G/G	1046:0	G/G	572:0	G/G
C/C	2300:0	C/C	2139:0	C/C	1046:0	C/C	572:0	C/T
A/A	471:0	A/A	51:0	A/A	91:0	A/A	89:0	A/A
G/G	471:0	G/G	51:0	G/G	91:0	G/G	89:0	G/G
G/G	955:0	G/G	1514:0	G/G	376:0	G/G	394:0	G/G
C/C	4335:0	C/C	3840:0	C/C	2141:0	C/C	1658:0	C/C

3591_A3_	8838_A_g	8838_A_r	4800_A3_	4800_A3_	6405_A2_	6405_A2_	7008_A1_	7008_A1_
2589:0	G/G	176:0	G/G	6321:0	G/G	6380:0	G/G	3180:0
2589:0	AGC/CGT	176:0	AGC/CGT	6321:0	AGC/CGT	6380:0	AGC/CGT	3180:0
1680:742	G/G	176:0	G/G	6321:0	G/G	6380:0	G/G	3180:0
2579:0	C/C	176:0	C/C	6321:0	C/C	6380:0	C/C	3180:0
2579:0	C/C	176:0	C/C	6321:0	C/C	6380:0	C/C	3180:0
2579:0	G/G	176:0	G/G	6321:0	G/G	6380:0	G/G	3180:0
2579:0	G/G	176:0	G/G	6321:0	G/G	6380:0	G/G	3180:0
2579:0	G/G	176:0	G/G	6321:0	G/G	6380:0	G/G	3180:0
2579:0	G/G	176:0	G/G	6321:0	G/G	6380:0	G/G	3180:0
2579:0	-GAGTTC	93:61	TTC/GGA	6321:0	TTC/GGA	6380:0	TTC/GGA	3180:0
2579:0	G/G	93:0	G/G	6321:0	G/G	6380:0	G/G	3180:0
2579:0	ACT/ATG	176:0	ACT/ATG	6321:0	ACT/ATG	6380:0	ACT/ATG	3180:0
2579:0	G/G	176:0	G/G	6321:0	G/G	6380:0	G/G	3180:0
2579:0	G/G	176:0	G/G	6321:0	G/G	6380:0	G/G	3180:0
1374:0	G/G	2241:0	G/G	4812:0	G/G	4225:0	G/G	2826:0
1374:0	C/C	2241:0	C/C	4812:0	C/C	4225:0	C/C	2826:0
1374:0	T/T	2241:0	T/T	4812:0	T/T	4225:0	T/T	2826:0
1374:0	-/-	2241:0	-/-	4812:0	-/-	4225:0	-/-	2826:0
1374:0	T/T	2241:0	T/T	4812:0	T/T	4225:0	T/T	2826:0
1374:0	TGC/ATA	2241:0	TGC/ATA	4812:0	TGC/ATA	4225:0	TGC/ATA	2826:0
1374:0	C/C	2241:0	C/C	4812:0	C/C	4225:0	C/C	2826:0
1374:0	G/G	2241:0	G/G	4812:0	G/G	4225:0	G/G	2830:0
180:0	AG/AG	170:0	AG/AG	504:0	AG/AG	498:0	AG/AG	316:0
180:0	T/T	170:0	T/T	504:0	T/T	498:0	T/T	316:0
680:0	C/C	563:0	C/C	500:0	C/C	1027:0	C/C	323:0
1062:0	T/T	1642:0	T/T	2752:0	T/T	2829:0	T/T	1709:0
1062:0	G/G	1642:0	G/G	2752:0	G/G	2829:0	G/G	1709:0
646:1349	C/C	1642:0	C/C	2752:0	C/C	2829:0	C/C	1709:0
190:0	A/A	188:0	A/A	443:0	A/A	565:0	A/A	335:0
190:0	G/G	188:0	G/G	443:0	G/G	565:0	G/G	335:0
898:0	G/G	673:0	G/G	1335:0	G/G	1795:0	G/G	685:0
1295:0	C/C	3244:0	C/C	4989:0	C/C	4376:0	C/C	2791:0

3365_A2_	3365_A2_	4413_A3_	4413_A3_	420_A2_	420_A2_	564_A1_	564_A1_	_18B0723
G/G	5304:0	G/G	4629:0	G/G	124:0	G/G	216:0	G/G
AGC/CGT	5304:0	AGC/CGT	4629:0	AGC/CGT	124:0	AGC/CGT	216:0	AGC/CGT
G/G	5304:0	G/G	4629:0	G/G	124:0	G/G	216:0	G/G
C/C	5304:0	C/C	4629:0	C/C	124:0	C/C	216:0	C/C
C/C	5304:0	C/C	4629:0	C/C	124:0	C/C	216:0	C/C
G/G	5304:0	G/G	4629:0	G/G	124:0	G/G	216:0	G/G
G/G	5304:0	G/G	4629:0	G/G	124:0	G/G	216:0	G/G
G/G	5304:0	G/G	4629:0	G/G	124:0	G/G	216:0	G/G
G/G	5304:0	G/G	4629:0	G/G	124:0	G/G	216:0	G/G
TTC/GGA	5304:0	TTC/GGA	4629:0	TTC/GGA	124:0	TTC/GGA	216:0	TTC/GGA
G/G	5304:0	G/G	4629:0	G/G	124:0	G/G	216:0	G/G
ACT/ATG	5304:0	ACT/ATG	4629:0	ACT/ATG	124:0	ACT/ATG	216:0	ACT/ATG
G/G	5304:0	G/G	4629:0	G/G	124:0	G/G	216:0	G/G
G/G	5304:0	G/G	4629:0	G/G	124:0	G/G	216:0	G/G
G/G	3904:0	G/G	2451:0	G/G	2531:0	G/G	2132:0	G/G
C/C	3904:0	C/C	2451:0	C/C	2531:0	C/C	2132:0	C/C
T/T	3904:0	T/T	2451:0	T/T	2531:0	T/T	2132:0	T/T
-/-	3904:0	-/-	2451:0	-/-	2531:0	-/-	2132:0	-/-
T/T	3904:0	T/T	2451:0	T/T	2531:0	T/T	2132:0	T/T
TGC/ATA	3904:0	TGC/ATA	2451:0	TGC/ATA	2531:0	TGC/ATA	2132:0	TGC/ATA
C/C	3904:0	C/C	2451:0	C/C	2531:0	C/C	2132:0	C/C
G/G	3904:0	G/G	2454:0	G/G	2531:0	G/G	2132:0	G/G
AG/AG	506:0	AG/AG	136:0	AG/AG	152:0	AG/AG	452:0	AG/AG
T/T	506:0	T/T	136:0	T/T	152:0	T/T	452:0	T/T
C/C	54:0	C/C	866:0	C/C	533:0	C/C	394:0	C/C
T/T	2775:0	T/T	1811:0	T/T	1344:0	T/T	1423:0	T/T
G/G	2775:0	G/G	1811:0	G/G	1344:0	G/G	1423:0	G/G
C/C	2775:0	C/C	1811:0	C/C	1344:0	C/C	1423:0	C/C
A/A	605:0	A/A	143:0	A/A	152:0	A/A	303:0	A/A
G/G	605:0	G/G	143:0	G/G	152:0	G/G	303:0	G/G
G/G	136:0	G/G	1432:0	G/G	988:0	G/G	682:0	G/G
C/C	3669:0	C/C	2946:0	C/C	3974:0	C/C	2452:0	C/C

_18B0723	_18B0723	_18B0723	_18B0723	_18B0723	0820_A2_	0820_A2_	4960_A2_	4960_A2_
4931:0	G/G	761:0	G/G	3367:0	G/G	1899:0	G/G	2352:0
4931:0	AGC/CGT	761:0	AGC/CGT	3367:0	AGC/CGT	1899:0	AGC/CGT	2352:0
4931:0	G/G	761:0	G/G	3367:0	G/G	1899:0	G/G	2352:0
4931:0	C/C	761:0	C/C	3367:0	C/C	1899:0	C/C	2352:0
4931:0	C/C	761:0	C/C	3367:0	C/C	1899:0	C/C	2352:0
4931:0	G/G	761:0	G/G	3367:0	G/G	1899:0	G/G	2352:0
4931:0	G/G	761:0	G/G	3367:0	G/G	1899:0	G/G	2352:0
4931:0	G/G	761:0	G/G	3367:0	G/G	1899:0	G/G	2352:0
4931:0	G/G	761:0	G/G	3367:0	G/G	1899:0	G/G	2352:0
4931:0	TTC/GGA	761:0	TTC/GGA	3367:0	TTC/GGA	1899:0	TTC/GGA	2352:0
4931:0	G/G	761:0	G/G	3367:0	G/G	1899:0	G/G	2352:0
4931:0	ACT/ATG	761:0	ACT/ATG	3367:0	ACT/ATG	1899:0	ACT/ATG	2352:0
4931:0	G/G	761:0	G/G	3367:0	G/G	1899:0	G/G	2352:0
4931:0	G/G	761:0	G/G	3367:0	G/G	1899:0	G/G	2352:0
2837:0	G/G	1687:0	G/G	2402:0	G/G	2338:0	G/G	1750:0
2837:0	C/C	1687:0	C/C	2402:0	C/C	2338:0	C/C	1750:0
2837:0	T/T	1687:0	T/T	2402:0	T/T	2338:0	T/T	1750:0
2837:0	-/-	1687:0	-/-	2402:0	-/-	2338:0	-/-	1750:0
2837:0	T/T	1687:0	T/C	1062:3475	T/T	2338:0	T/T	1750:0
2837:0	TGC/ATA	1687:0	TGC/ATA	2407:0	TGC/ATA	2338:0	TGC/ATA	1750:0
2837:0	C/C	1687:0	C/C	2407:0	C/C	2338:0	C/C	1750:0
2837:0	G/G	1687:0	G/G	2407:0	G/G	2338:0	G/G	1750:0
278:0	AG/AG	367:0	AG/AG	362:0	AG/AG	227:0	AG/AG	213:0
278:0	T/T	367:0	T/T	362:0	T/T	227:0	T/T	213:0
804:0	C/C	578:0	C/C	806:0	C/C	858:0	C/C	469:0
1620:0	T/T	1489:0	T/T	1552:0	T/T	1673:0	T/T	1363:0
1620:0	G/G	1489:0	G/G	1552:0	G/G	1673:0	G/G	1363:0
1620:0	C/C	1489:0	C/C	1552:0	C/C	1673:0	C/C	1363:0
289:0	A/A	269:0	A/A	306:0	A/A	285:0	A/A	230:0
289:0	G/G	269:0	G/G	306:0	G/G	285:0	G/G	230:0
1364:0	G/G	891:0	G/G	1117:0	G/G	1361:0	G/G	909:0
3240:0	C/C	2378:0	C/C	2641:0	C/C	2679:0	C/C	1897:0

5988_A3_	988_A3_	0329_A3_	329_A3_	3390_A2_	3390_A2_	_18B0723	_18B0723	0464_A3_
G/G	82:0	G/G	4325:0	G/G	7133:0	G/G	4780:0	G/G
AGC/CGT	82:0	AGC/CGT	4325:0	AGC/CGT	7133:0	AGC/CGT	4780:0	AGC/CGT
G/G	82:0	G/G	4325:0	G/G	7133:0	G/G	4780:0	G/G
C/C	82:0	C/C	4325:0	C/C	7133:0	C/C	4780:0	C/C
C/C	82:0	C/C	4325:0	C/T	4630:1860	C/C	4780:0	C/C
G/G	82:0	G/G	4325:0	G/G	7121:0	G/G	4780:0	G/G
G/G	82:0	G/G	4325:0	G/G	7121:0	G/G	4780:0	G/G
G/G	82:0	G/G	4325:0	G/G	7121:0	G/G	4780:0	G/G
G/G	82:0	G/G	4325:0	G/G	7121:0	G/G	4780:0	G/G
TTC/GGA	82:0	TTC/GGA	4325:0	TTC/GGA	7121:0	TTC/GGA	4780:0	TTC/GGA
G/G	82:0	G/G	4325:0	G/G	7121:0	G/G	4780:0	G/G
ACT/ATG	82:0	ACT/ATG	4325:0	ACT/ATG	7121:0	ACT/ATG	4780:0	ACT/ATG
G/G	82:0	G/G	4325:0	G/G	7121:0	G/G	4780:0	G/G
G/G	82:0	G/G	4325:0	G/G	7121:0	G/G	4780:0	G/G
G/G	2482:0	G/G	3581:0	G/G	5290:0	G/G	2416:0	G/G
C/C	2482:0	C/C	3581:0	C/C	5290:0	C/C	2416:0	C/C
T/T	2482:0	T/T	3581:0	T/T	5290:0	T/T	2416:0	T/T
-/-	2482:0	-/-	3581:0	-/-	5290:0	-/-	2416:0	-/-
T/T	2482:0	T/T	3581:0	T/T	5290:0	T/T	2416:0	T/T
TGC/ATA	2482:0	TGC/ATA	3581:0	TGC/ATA	5290:0	TGC/ATA	2416:0	TGC/ATA
C/C	2482:0	C/C	3581:0	C/C	5290:0	C/C	2416:0	C/C
G/G	2482:0	G/G	3592:0	G/G	5290:0	G/G	2420:0	G/G
AG/AG	71:0	AG/AG	312:0	AG/AG	804:0	AG/AG	165:0	AG/AG
T/T	71:0	T/T	312:0	T/T	804:0	T/T	165:0	T/T
C/C	564:0	C/C	629:0	C/C	251:0	C/C	928:0	C/C
T/T	1339:0	T/T	2776:0	T/-	3915:650	T/T	1994:0	T/T
G/G	1339:0	G/G	2776:0	G/G	3876:0	G/G	1994:0	G/G
C/C	1339:0	C/C	2776:0	C/C	3876:0	C/C	1994:0	C/C
A/A	83:0	A/A	321:0	A/A	649:0	A/A	181:0	A/A
G/G	83:0	G/G	321:0	G/G	649:0	G/G	181:0	G/G
G/G	1030:0	G/G	1137:0	G/G	581:0	G/G	1509:0	G/G
C/C	3620:0	C/C	3647:0	C/C	5230:0	C/C	3093:0	C/C

0464_A3_r	2857_A2_g	2857_A2_r	3723_A2_g	3723_A2_r	1426_B1_g	426_B1_r	0230_A3_g	0230_A3_r
6873:0	G/G	5429:0	G/G	5346:0	G/G	101:0	G/G	4728:0
6873:0	AGC/CGT	5429:0	AGC/CGT	5346:0	AGC/CGT	101:0	AGC/CGT	4728:0
6873:0	G/G	5429:0	G/G	5346:0	G/G	101:0	G/G	4728:0
6873:0	C/C	5429:0	C/C	5346:0	C/C	101:0	C/C	4728:0
6873:0	C/C	5429:0	C/C	5346:0	C/C	101:0	C/C	4728:0
6873:0	G/G	5429:0	G/G	5346:0	G/G	101:0	G/G	4728:0
6873:0	G/G	5429:0	G/G	5346:0	G/G	101:0	G/G	4728:0
6873:0	G/G	5429:0	G/G	5346:0	G/G	101:0	G/G	4728:0
6873:0	G/G	5429:0	G/G	5346:0	G/G	101:0	G/G	4728:0
6873:0	TTC/GGA	5429:0	TTC/GGA	5346:0	TTC/GGA	101:0	TTC/GGA	4728:0
6873:0	G/G	5429:0	G/G	5346:0	G/G	101:0	G/G	4728:0
6873:0	ACT/ATG	5429:0	ACT/ATG	5346:0	ACT/ATG	101:0	ACT/ATG	4728:0
6873:0	G/A	2231:2383	G/G	5346:0	G/G	101:0	G/G	4728:0
6873:0	G/G	5449:0	G/G	5346:0	G/G	101:0	G/G	4728:0
3226:0	G/G	2540:0	G/G	2310:0	G/G	2259:0	G/G	2363:0
3226:0	C/C	2540:0	C/C	2310:0	C/C	2259:0	C/C	2363:0
3226:0	T/T	2540:0	T/T	2310:0	T/T	2259:0	T/T	2363:0
3226:0	-/-	2540:0	-/-	2310:0	-/-	2259:0	-/-	2363:0
3226:0	T/T	2540:0	T/T	2310:0	T/T	2259:0	T/T	2363:0
3226:0	TGC/ATA	2540:0	TGC/ATA	2310:0	TGC/ATA	2259:0	AACGTG	3323:1003
3226:0	C/C	2540:0	C/C	2310:0	C/C	2259:0	C/C	2369:0
3226:0	G/G	2540:0	G/G	2310:0	G/G	2259:0	G/G	2369:0
457:0	AG/AG	243:0	AG/AG	256:0	AG/AG	147:0	AG/AG	426:0
457:0	T/T	243:0	T/T	256:0	T/T	147:0	T/T	426:0
839:0	C/C	867:0	C/C	959:0	C/C	509:0	C/C	760:0
1477:0	T/T	1818:0	T/T	1862:0	T/T	1606:0	T/T	1903:0
1477:0	G/G	1818:0	G/G	1862:0	G/G	1606:0	G/G	1903:0
1477:0	C/C	1818:0	C/C	1862:0	C/C	1606:0	C/C	1903:0
451:0	A/A	255:0	A/A	271:0	A/A	131:0	A/A	369:0
451:0	G/G	255:0	G/G	271:0	G/G	131:0	G/G	369:0
1274:0	G/G	2110:0	G/G	1492:0	G/G	908:0	G/G	1053:0
3189:0	C/C	3552:0	C/C	2887:0	C/C	3413:0	C/C	2672:0

3231_A1_	231_A1_	47_A1_ne	47_A1_net	1910_A1_	910_A1_	7017_A1_	017_A1_	3491_B1_
G/G	9824:0	G/G	6886:0	G/G	5362:0	G/G	659:0	G/G
AGC/CGT	9824:0	AGC/CGT	6886:0	AGC/CGT	5362:0	AGC/CGT	659:0	AGC/CGT
G/G	9824:0	G/G	6886:0	G/G	5362:0	G/G	659:0	G/G
C/C	9824:0	C/C	6886:0	C/C	5362:0	C/C	659:0	C/C
C/C	9824:0	C/C	6886:0	C/C	5362:0	C/C	659:0	C/C
G/G	9824:0	G/G	6886:0	G/G	5362:0	G/G	659:0	G/G
G/G	9824:0	G/G	6886:0	G/G	5362:0	G/G	659:0	G/G
G/G	9824:0	G/G	6886:0	G/G	5362:0	G/G	659:0	G/G
G/G	9824:0	G/G	6886:0	G/G	5362:0	G/G	659:0	G/G
TTC/GGA	9824:0	TTC/GGA	6886:0	TTC/GGA	5362:0	TTC/GGA	659:0	TTC/GGA
G/G	9824:0	G/G	6886:0	G/G	5362:0	G/G	659:0	G/G
ACT/ATG	9824:0	ACT/ATG	6886:0	ACT/ATG	5362:0	ACT/ATG	659:0	ACT/ATG
G/G	9824:0	G/G	6886:0	G/G	5362:0	G/G	659:0	G/G
G/G	9824:0	G/G	6886:0	G/G	5362:0	G/G	659:0	G/G
G/G	3947:0	G/G	2774:0	G/G	2649:0	G/G	1739:0	G/G
C/C	3947:0	C/C	2774:0	C/C	2649:0	C/C	1739:0	C/C
T/T	3947:0	T/T	2774:0	T/T	2649:0	T/T	1739:0	T/T
-/-	3947:0	-/-	2774:0	-/-	2649:0	-/-	1739:0	-/-
T/T	3947:0	T/T	2774:0	T/T	2649:0	T/T	1739:0	T/T
TGC/ATA	3947:0	TGC/ATA	2774:0	TGC/ATA	2649:0	TGC/ATA	1739:0	TGC/ATA
C/C	3947:0	C/C	2774:0	C/C	2649:0	C/C	1739:0	C/C
G/G	3947:0	G/G	2778:0	G/G	2655:0	G/G	1739:0	G/G
AG/AG	580:0	AG/AG	211:0	AG/AG	270:0	AG/AG	162:0	AG/AG
T/T	580:0	T/T	211:0	T/T	270:0	T/T	162:0	T/T
C/C	1102:0	C/C	849:0	C/C	904:0	C/C	441:0	C/C
T/T	1798:0	T/T	1815:0	T/T	1667:0	T/T	1116:0	T/T
G/G	1798:0	G/G	1815:0	G/G	1667:0	G/G	1116:0	G/G
C/C	1798:0	C/C	1815:0	C/C	1667:0	C/C	1116:0	C/C
A/A	523:0	A/A	251:0	A/A	246:0	A/A	173:0	A/A
G/G	523:0	G/G	251:0	G/G	246:0	G/G	173:0	G/G
G/G	1728:0	G/G	1611:0	G/G	1370:0	G/G	1025:0	G/G
C/C	4321:0	C/C	3320:0	C/C	2946:0	C/C	2161:0	C/C

3491_B1_	9519_A2_	9519_A2_	4566_A1_	4566_A1_	4571_A2_	4571_A2_	154-bach2_	54-bach2_
							C/C	1377:0
							G/G	121:0
							C/C	7:0
4277:0	G/G	2251:0	G/G	2596:0	G/G	7116:0		
4277:0	AGC/CGT	2251:0	AGC/CGT	2596:0	AGC/CGT	7116:0		
4277:0	G/G	2251:0	G/G	2596:0	G/G	7116:0		
4277:0	C/C	2251:0	C/C	2596:0	C/C	7116:0		
4277:0	C/C	2251:0	C/C	2596:0	C/C	7116:0		
4277:0	G/G	2251:0	G/G	2596:0	G/G	7116:0		
4277:0	G/G	2251:0	G/G	2596:0	G/G	7116:0		
4277:0	G/G	2251:0	G/G	2596:0	G/G	7116:0		
4277:0	G/G	2251:0	G/G	2596:0	G/G	7116:0		
4277:0	TTC/GGA	2251:0	TTC/GGA	2596:0	TTC/GGA	7116:0		
4277:0	G/G	2251:0	G/G	2596:0	G/G	7116:0		
4277:0	ACT/ATG	2251:0	ACT/ATG	2596:0	ACT/ATG	7116:0		
4277:0	G/G	2251:0	G/G	2596:0	G/G	7116:0		
4277:0	G/G	2251:0	G/G	2596:0	G/G	7116:0		
2082:0	G/G	2293:0	G/G	2095:0	G/G	3394:0		
2082:0	C/C	2293:0	C/C	2095:0	C/C	3394:0		
2082:0	T/T	2293:0	T/T	2095:0	T/T	3394:0		
2082:0	-/-	2293:0	-/-	2095:0	-/-	3394:0		
2082:0	T/T	2293:0	T/T	2095:0	T/T	3394:0		
2082:0	TGC/ATA	2293:0	TGC/ATA	2095:0	TGC/ATA	3394:0		
2082:0	C/C	2293:0	C/C	2095:0	C/C	3394:0		
2085:0	G/G	2293:0	G/G	2095:0	G/G	3402:0		
268:0	AG/AG	300:0	AG/AG	373:0	AG/AG	374:0		
268:0	T/T	300:0	T/T	373:0	T/T	374:0		
853:0	C/C	833:0	C/C	402:0	C/C	963:0		
1556:0	T/T	1582:0	T/T	1453:0	T/T	1928:0		
1556:0	G/G	1582:0	G/G	1453:0	G/G	1928:0		
1556:0	C/C	1582:0	C/C	1453:0	C/C	1928:0		
230:0	A/A	277:0	A/A	323:0	A/A	380:0		
230:0	G/G	277:0	G/G	323:0	G/G	380:0		
1393:0	G/G	1477:0	G/G	712:0	G/G	1623:0		
2038:0	C/A	2844:2355	C/C	1931:0	C/C	4031:0		
							G/G	84:0
							C/C	190:0
							C/C	190:0
							G/G	87:0
							CCC/GCA	97:0
							C/C	37:0
							C/C	45:0
							G/G	108:0

							C/C	527:0
							A/A	108:0
							G/G	84:0
							A/A	18:0

09154_ge	09154_rea	28591_ge	28591_rea	28839_ge	28839_rea	34800_ge	34800_rea	36405_ge
C/C	1267:0	C/C	1644:0	C/C	1756:0	C/C	1053:0	C/C
G/G	135:0	G/G	2432:0	G/G	2088:0	G/G	279:0	G/G
C/C	5:0	C/C	146:0	C/C	137:0	C/C	17:0	C/C
G/G	221:0	G/G	126:0	G/G	273:0	G/G	401:0	G/G
C/C	474:0	C/C	1345:0	C/C	1064:0	C/C	163:0	C/C
C/C	474:0	C/C	1345:0	C/C	1064:0	C/C	163:0	C/C
G/G	133:0	G/G	1615:0	G/G	846:0	G/G	42:0	G/G
CCC/GCA	46:0	CCC/GCA	304:0	CCC/GCA	119:0	CCC/GCA	183:0	CCC/GCA
C/C	124:0	C/C	175:0	C/C	27:0	C/C	603:0	C/C
C/C	38:0	C/C	622:0	C/C	387:0	C/C	22:0	C/C
G/G	266:0	G/G	349:0	G/G	282:0	G/G	478:0	G/G

C/C	506:0	C/C	360:0	C/C	427:0	C/C	224:0	C/C
A/A	266:0	A/A	349:0	A/A	5908:0	A/A	2017:0	A/A
G/G	1340:0	G/G	708:0	G/G	566:0	G/G	146:0	G/G
A/A	46:0	A/A	220:0	A/A	297:0	A/A	36:0	A/A

36405_rea	37008_ge	37008_rea	43365_ge	43365_rea	46420_ge	46420_rea	66564_ge	66564_rea
1913:0	C/C	1813:0	C/C	1452:0	C/C	1182:0	C/C	1571:0
3195:0	G/G	2300:0	G/G	1710:0	G/G	1286:0	G/G	572:0
170:0	C/T	36:37	C/C	70:0	C/C	17:0	C/C	29:0
256:0	G/G	188:0	G/G	216:0	G/G	69:0	G/G	273:0
1294:0	C/C	1018:0	C/C	1426:0	C/C	1542:0	C/C	276:0
1294:0	C/C	1018:0	C/C	1426:0	C/C	1542:0	C/C	276:0
1033:0	G/G	784:0	G/G	1054:0	G/G	498:0	G/G	113:0
145:0	CCC/GCA	905:0	CCC/GCA	117:0	CCC/GCA	108:0	CCC/GCA	34:0
95:0	C/C	101:0	C/C	214:0	C/C	22:0	C/C	62:0
373:0	C/C	350:0	C/C	393:0	C/C	159:0	C/C	96:0
303:0	G/G	236:0	G/G	284:0	G/G	74:0	G/G	137:0

386:0	C/C	360:0	C/C	541:0	C/C	286:0	C/C	481:0
303:0	A/A	236:0	A/A	4434:0	A/A	73:0	A/A	137:0
590:0	G/G	1109:0	G/G	1666:0	G/G	968:0	G/G	566:0
417:0	A/A	227:0	A/T	193:65	A/A	54:0	A/A	75:0

82805_ge	82805_rea	83561_ge	83561_rea	95855_ge	95855_rea	10820_ge	10820_rea	24960_ge
C/C	1671:0	C/C	1474:0	C/C	2210:0	C/C	1822:0	C/C
G/G	2558:0	G/G	221:0	G/T	2443:911	G/G	2191:0	G/G
C/C	83:0	C/C	6:0	C/C	98:0	C/C	81:0	C/C
G/G	142:0	G/G	348:0	G/G	206:0	G/G	112:0	G/G
C/C	1162:0	C/C	217:0	C/C	1312:0	C/C	1091:0	C/C
C/C	1162:0	C/C	217:0	C/C	1312:0	C/C	1091:0	C/C
G/G	1781:0	G/G	50:0	G/G	1387:0	G/G	763:0	G/G
CCC/GCA	451:0	CCC/GCA	161:0	CCC/GCA	252:0	CCC/GCA	111:0	CCC/GCA
C/C	183:0	C/C	67:0	C/C	230:0	C/C	12:0	C/C
C/C	641:0	C/C	36:0	C/C	511:0	C/C	334:0	C/C
G/G	321:0	G/G	88:0	G/G	269:0	G/G	209:0	G/G

C/C	551:0	C/C	272:0	C/C	500:0	C/C	473:0	C/C
A/A	184:0	A/G	1494:163	A/A	268:0	A/A	471:0	A/A
G/G	851:0	G/G	344:0	G/G	359:0	G/G	787:0	G/G
A/A	180:0	A/T	20:16	A/A	233:0	A/A	173:0	A/A

24960_rea	26988_ge	26988_rea	30329_ge	30329_rea	43390_ge	43390_rea	53948_ge	53948_rea
1345:0	C/C	1699:0	C/C	1676:0	C/C	1920:0	C/C	1845:0
939:0	G/G	916:0	G/G	1544:0	G/G	2018:0	G/G	2648:0
40:0	C/C	21:0	C/C	90:0	C/C	96:0	C/C	78:0
342:0	G/G	83:0	G/G	274:0	G/G	239:0	G/G	54:0
751:0	C/C	1042:0	C/C	1333:0	C/C	1024:0	C/C	1198:0
751:0	C/C	1042:0	C/C	1333:0	C/C	1024:0	C/C	1198:0
404:0	G/G	672:0	G/G	1151:0	G/G	1183:0	G/G	1479:0
39:0	CCC/GCA	201:0	CCC/GCA	111:0	CCC/GCA	187:0	CCC/GCA	616:0
354:0	C/C	139:0	C/C	34:0	C/C	226:0		
124:0	C/C	216:0	C/C	413:0	C/C	465:0	C/C	412:0
403:0	G/G	118:0	G/G	244:0	G/G	596:0	G/G	502:0

448:0	C/C	340:0	C/C	585:0	C/C	462:0	C/C	325:0
5277:0	A/A	335:0	A/A	244:0	A/A	457:0	A/A	316:0
518:0	G/G	1627:0	G/G	817:0	G/G	520:0	G/G	856:0
139:0	A/A	45:0	A/A	257:0	A/A	220:0	A/A	150:0

60464_ge	60464_rea	72857_ge	72857_rea	73723_ge	73723_rea	81426_ge	81426_rea	09230_ge
C/C	1741:0	C/C	1697:0	C/A	6614:5690	C/C	1770:0	C/C
G/G	2359:0	G/G	1528:0	G/G	2250:0	G/G	548:0	G/G
C/C	159:0	C/C	88:0	C/C	153:0	C/C	12:0	C/C
G/G	267:0	G/G	297:0	G/G	206:0	G/G	258:0	G/G
C/C	1338:0	C/C	1056:0	C/C	1426:0	C/C	1226:0	C/C
C/C	1338:0	C/C	1056:0	C/C	1426:0	C/C	1226:0	C/C
G/G	1332:0	G/G	1065:0	G/G	1474:0	G/G	666:0	G/G
CCC/GCA	250:0	CCC/GCA	112:0	CCC/GCA	226:0	CCC/GCA	50:0	CCC/GCA
C/C	29:0	C/C	24:0	C/C	20:0	C/C	15:0	C/C
C/C	267:0	C/C	398:0	C/C	570:0	C/C	266:0	C/C
G/G	292:0	G/G	251:0	G/G	770:0	G/G	266:0	G/G

C/C	524:0	C/C	627:0	C/C	600:0	C/C	633:0	C/C
A/A	292:0	A/A	251:0	A/A	596:0	A/A	265:0	A/A
G/G	179:0	G/G	753:0	G/G	764:0	G/G	3342:0	G/G
A/A	329:0	A/A	195:0	A/A	217:0	A/A	39:0	A/A

09230_rea	13231_ge	13231_rea	17247_ge	17247_rea	20253_ge	20253_rea	21910_ge	21910_rea
1420:0	C/C	1558:0	C/C	1666:0	C/C	955:0	C/C	2034:0
1739:0	G/G	2865:0	G/G	2247:0	G/G	2075:0	G/G	2796:0
105:0	C/C	208:0	C/C	162:0	C/C	120:0	C/C	185:0
275:0	G/G	491:0	G/G	407:0	G/G	385:0	G/G	107:0
1198:0	C/C	1701:0	C/C	1317:0	C/C	846:0	C/C	1323:0
1198:0	C/C	1701:0	C/C	1317:0	C/C	846:0	C/C	1323:0
1260:0	G/G	2075:0	G/G	1541:0	G/G	1179:0	G/G	1533:0
145:0	CCC/GCA	324:0	CCC/GCA	140:0	CCC/GCA	77:0	CCC/GCA	320:0
32:0	C/C	57:0	C/C	45:0	C/C	371:0	C/C	169:0
405:0	C/C	622:0	C/C	284:0	C/C	544:0	C/C	908:0
650:0	G/G	416:0	G/G	337:0	G/G	319:0	G/G	248:0

525:0	C/C	614:0	C/C	545:0	C/C	517:0	C/C	398:0
4804:0	A/A	249:0	A/A	337:0	A/A	319:0	A/A	4877:0
487:0	G/G	330:0	G/G	386:0	G/G	200:0	G/G	426:0
180:0	A/A	342:0	A/A	248:0	A/A	181:0	A/A	210:0

22751_ge	22751_rea	27017_ge	27017_rea	28491_ge	28491_rea	49519_ge	49519_rea	64566_ge
C/C	2575:0	C/C	2334:0	C/C	2021:0	C/C	2094:0	C/C
G/G	1984:0	G/G	1061:0	G/G	2552:0	G/G	2833:0	G/G
C/C	62:0	C/C	19:0	C/C	147:0	C/C	226:0	C/C
G/G	143:0	G/G	159:0	G/G	239:0	G/G	351:0	G/G
C/C	1574:0	C/C	1316:0	C/C	786:0	C/C	774:0	C/C
C/C	1574:0	C/C	1316:0	C/C	786:0	C/C	774:0	C/C
G/G	1535:0	G/G	866:0	G/G	1167:0	G/G	1466:0	G/G
CCC/GCA	363:0	CCC/GCA	177:0	CCC/GCA	227:0	CCC/GCA	179:0	CCC/GCA
C/C	228:0	C/C	84:0	C/C	257:0	C/C	359:0	C/C
C/C	925:0	C/C	345:0	C/C	426:0	C/C	569:0	C/C
G/G	168:0	G/G	403:0	G/G	182:0	G/G	292:0	G/G

C/C	527:0	C/C	666:0	C/C	389:0	C/C	461:0	C/C
A/A	167:0	A/A	657:0	A/A	182:0	A/A	6693:0	A/A
G/G	745:0	G/G	939:0	G/G	264:0	G/G	102:0	G/G
A/A	164:0	A/A	57:0	A/A	289:0	A/A	332:0	A/A

64566_rea	64571_ge	64571_rea	26080_ge	26080_rea	26748_ge	26748_rea	30807_ge	30807_rea
1902:0	C/C	1724:0	C/C	1121:0	C/C	1140:0	C/C	1851:0
1732:0	G/G	3956:0	G/G	664:0	G/G	540:0	G/G	1651:0
113:0	C/C	285:0	C/C	39:0	C/C	32:0	C/C	31:0
465:0	G/G	293:0	G/G	79:0	G/G	238:0	G/G	138:0
853:0	C/C	1074:0	C/C	450:0	C/C	517:0	C/C	481:0
853:0	C/C	1074:0	C/C	450:0	C/C	517:0	C/C	481:0
1157:0	G/G	1319:0	G/G	323:0	G/G	402:0	G/G	380:0
63:0	CCC/GCA	184:0	CCC/GCA	412:0	CCC/GCA	64:0	CCC/GCA	101:0
41:0	C/C	88:0	C/C	38:0	C/C	55:0	C/C	149:0
343:0	C/C	511:0	C/C	427:0	C/C	264:0	C/C	154:0
344:0	G/G	161:0	G/A	265:271	G/G	157:0	G/G	526:0

369:0	C/C	333:0	C/C	255:0	C/C	312:0	C/C	464:0
6028:0	A/A	5197:0	A/A	251:0	A/A	311:0	A/A	449:0
204:0	G/G	89:0	G/G	390:0	G/G	139:0	G/G	1044:0
169:0	A/A	485:0	A/A	97:0	A/A	159:0	A/A	93:0

68167_gen	68167_rea	75537_gen	75537_rea	77228_gen	77228_rea	99137_gen	99137_rea	22715_gen
C/C	1867:0	C/C	2071:0	C/C	1726:0	C/C	604:0	C/C
G/G	695:0	G/G	917:0	G/G	338:0	G/G	703:0	G/G
C/C	27:0	C/C	22:0	C/C	8:0	C/C	8:0	
G/G	379:0	G/G	231:0	G/G	394:0	G/G	67:0	G/G
C/C	656:0	C/C	871:0	C/C	325:0	C/C	273:0	C/T
C/C	656:0	C/C	871:0	C/C	325:0	C/C	273:0	C/C
G/G	381:0	G/G	501:0	G/G	168:0	G/G	314:0	G/G
CCC/GCA	597:0	CCC/GCA	713:0	CCC/GCA	275:0	CCC/GCA	75:0	CCC/GCA
C/C	275:0	C/C	37:0	C/C	340:0			C/C
C/C	164:0	C/C	188:0	C/C	83:0	C/C	265:0	
G/G	154:0	G/G	338:0	G/G	152:0	G/G	66:0	G/G

C/C	303:0	C/C	408:0	C/C	376:0	C/C	169:0	C/T
A/A	152:0	A/A	404:0	A/A	152:0	A/A	166:0	A/A
G/G	498:0	G/G	1389:0	G/G	1123:0	G/G	893:0	G/G
A/A	115:0	A/A	134:0	A/A	41:0	A/A	30:0	A/A

22715_rea	35992_gen	35992_rea	44335_gen	44335_rea	57428_gen	57428_rea	60066_gen	60066_rea
563:0	C/C	606:0	C/C	2143:0	C/C	550:0	C/C	405:0
50:0	G/G	159:0	G/G	1282:0	G/G	81:0	G/G	53:0
	C/C	7:0	C/C	55:0				
61:0	G/G	347:0	G/G	377:0	G/G	105:0	G/G	35:0
18:5	C/C	103:0	C/C	1145:0	C/C	64:0	C/C	45:0
24:0	C/C	103:0	C/C	1145:0	C/C	64:0	C/C	45:0
6:0	G/G	34:0	G/G	800:0	G/G	29:0	G/G	31:0
21:0	CCC/GCA	76:0	CCC/GCA	103:0	CCC/GCA	60:0	CCC/GCA	63:0
58:0	C/C	30:0	C/C	379:0	C/C	100:0		
	C/C	9:0	C/C	36:0	C/C	14:0	C/C	9:0
551:0	G/G	177:0	G/G	196:0	G/G	70:0	G/G	85:0

130:16	C/C	230:0	C/C	679:0	C/C	184:0	C/C	83:0
998:0	A/A	1587:0	A/A	196:0	A/A	1291:0	A/A	441:0
230:0	G/G	865:0			G/G	453:0	G/G	175:0
26:0	A/A	24:0	A/A	183:0	A/A	32:0	A/A	24:0

69110_ger	69110_rea	72291_ger	72291_rea	80268_ger	80268_rea	90451_ger	90451_rea	808_A2_g
C/C	2059:0	C/C	1905:0	C/C	1643:0	C/C	1727:0	
G/G	887:0	G/G	1337:0	G/G	1710:0	G/G	1601:0	
C/C	42:0	C/C	66:0	C/C	29:0	C/C	46:0	
								G/G
								AGC/CGT
								G/G
								C/C
								C/C
								G/G
								G/G
								G/G
								G/G
								TTC/GGA
								G/G
								ACT/ATG
								G/G
								G/G
								G/G
								C/G
								T/T
								-/-
								T/T
								TGC/ATA
								C/C
								G/G
								AG/AG
								T/T
								C/C
								T/T
								G/G
								C/C
								A/A
								G/G
								G/G
								C/C
G/G	284:0	G/G	228:0	G/G	89:0	G/G	77:0	
C/C	1124:0	C/C	1258:0	C/C	787:0	C/C	1008:0	
C/C	1124:0	C/C	1258:0	C/C	787:0	C/C	1008:0	
G/G	503:0	G/G	927:0	G/G	645:0	G/G	946:0	
CCC/GCA	42:0	CCC/GCA	76:0	CCC/GCA	112:0	CCC/GCA	212:0	
C/C	45:0	C/C	267:0	C/C	94:0	C/C	155:0	
C/C	252:0	C/C	403:0	C/C	250:0	C/C	387:0	
G/G	467:0	G/G	167:0	G/G	177:0	G/G	220:0	

C/C	622:0	C/C	517:0	C/C	412:0	C/C	363:0	
A/A	613:0	A/A	167:0	A/A	178:0	A/A	218:0	
		G/G	1262:0	G/G	2467:0	G/G	1621:0	
A/A	128:0	A/A	251:0	A/A	93:0	A/A	214:0	

808_A2_r	646_A3_	646_A3_	746_A1_	746_A1_	625_A5_	625_A5_	503_A1_	503_A1_
1629:0	G/G	2410:0	G/G	4694:0	G/G	1598:0	G/G	3785:0
1629:0	AGC/CGT	2410:0	AGC/CGT	4694:0	AGC/CGT	1598:0	AGC/CGT	3785:0
1629:0	G/G	2410:0	G/G	4694:0	G/G	1598:0	G/G	3785:0
1629:0	C/C	2410:0	C/C	4694:0	C/C	1598:0	C/C	3785:0
1629:0	C/C	2410:0	C/C	4694:0	C/C	1598:0	C/C	3785:0
1629:0	G/G	2410:0	G/G	4694:0	G/G	1598:0	G/G	3785:0
1629:0	G/G	2410:0	G/G	4694:0	G/G	1598:0	G/G	3785:0
1629:0	G/G	2410:0	G/G	4694:0	G/G	1598:0	G/G	3785:0
1629:0	G/G	2410:0	G/G	4694:0	G/G	1598:0	G/G	3785:0
1629:0	TTC/GGA	2410:0	TTC/GGA	4694:0	TTC/GGA	1598:0	TTC/GGA	3785:0
1629:0	G/G	2410:0	G/G	4694:0	G/G	1598:0	G/G	3785:0
1629:0	ACT/ATG	2410:0	ACT/ATG	4694:0	ACT/ATG	1598:0	ACT/ATG	3785:0
1629:0	G/G	2410:0	G/G	4694:0	G/G	1598:0	G/G	3785:0
1629:0	G/G	2410:0	G/G	4694:0	G/G	1598:0	G/G	3785:0
2569:0	G/G	2550:0	G/G	2624:0	G/G	2180:0	G/G	2009:0
2883:739	C/C	2550:0	C/C	2624:0	C/C	2180:0	C/C	2009:0
2579:0	T/T	2550:0	T/T	2624:0	T/T	2180:0	T/T	2009:0
2579:0	-/-	2550:0	-/-	2624:0	-/-	2180:0	-/-	2009:0
2579:0	T/T	2550:0	T/T	2624:0	T/T	2180:0	T/T	2009:0
2579:0	TGC/ATA	2550:0	TGC/ATA	2624:0	TGC/ATA	2180:0	TGC/ATA	2009:0
2579:0	C/C	2550:0	C/C	2624:0	C/C	2180:0	C/C	2009:0
2579:0	G/G	2558:0	G/G	2624:0	G/G	2180:0	G/G	2009:0
295:0	AG/AG	231:0	AG/AG	225:0	AG/AG	155:0	AG/AG	251:0
295:0	T/T	231:0	T/T	225:0	T/T	155:0	T/T	251:0
592:0	C/C	808:0	C/C	1059:0	C/C	506:0	C/C	940:0
1967:0	T/T	1545:0	T/T	2228:0	T/T	1767:0	T/T	1535:0
1967:0	G/G	1545:0	G/G	2228:0	G/G	1767:0	G/G	1535:0
1967:0	C/C	1545:0	C/C	2228:0	C/C	1767:0	C/C	1535:0
285:0	A/A	266:0	A/A	295:0	A/A	205:0	A/A	237:0
285:0	G/G	266:0	G/G	295:0	G/G	205:0	G/G	237:0
998:0	G/G	922:0	G/G	1607:0	G/G	808:0	G/G	1079:0
3354:0	C/C	2664:0	C/C	3450:0	C/C	2964:0	C/C	2577:0

5897_A1_	5897_A1_	3763_B2_	3763_B2_	5989_A3_	5989_A3_	7285_F2_	7285_F2_	4_18B0723
G/G	1006:0	G/G	3009:0	G/G	2778:0	G/G	100:0	G/G
AGC/CGT	1006:0	AGC/CGT	3009:0	AGC/CGT	2778:0	AGC/CGT	99:0	AGC/CGT
G/G	1006:0	G/G	3009:0	G/G	2778:0	G/G	99:0	G/G
C/-	293:638	C/C	3009:0	C/C	2778:0	C/C	99:0	C/C
C/C	1003:0	C/C	3009:0	C/C	2778:0	C/C	99:0	C/C
G/G	1003:0	G/G	3009:0	G/G	2778:0	G/G	99:0	G/G
G/G	1003:0	G/G	3009:0	G/G	2778:0	G/G	99:0	G/G
G/G	1003:0	G/G	3009:0	G/G	2778:0	G/G	99:0	G/G
G/G	1003:0	G/G	3009:0	G/G	2778:0	G/G	99:0	G/G
TTC/GGA	1003:0	TTC/GGA	3009:0	TTC/GGA	2778:0	TTC/GGA	99:0	TTC/GGA
G/G	1003:0	G/G	3009:0	G/G	2778:0	G/G	99:0	G/G
ACT/ATG	1003:0	ACT/ATG	3009:0	ACT/ATG	2778:0	ACT/ATG	99:0	ACT/ATG
G/G	1003:0	G/G	3009:0	G/G	2778:0	G/G	99:0	G/G
G/G	1003:0	G/G	3009:0	G/G	2778:0	G/G	99:0	G/G
G/G	1168:0	G/G	3586:0	G/G	1875:0	G/G	1208:0	G/G
C/C	1168:0	C/C	3586:0	C/G	1012:689	C/G	192:855	C/C
T/T	1168:0	T/T	3586:0	T/T	1879:0	T/T	1214:0	T/T
-/-	1168:0	-/-	3586:0	-/-	1879:0	-/-	1214:0	-/-
T/T	1168:0	T/T	3586:0	T/T	1879:0	T/T	1214:0	T/T
TGC/ATA	1168:0	TGC/ATA	3586:0	TGC/ATA	1879:0	TGC/ATA	1214:0	TGC/ATA
C/C	1168:0	C/C	3586:0	C/C	1879:0	C/C	1214:0	C/C
G/G	1168:0	G/G	3586:0	G/G	1879:0	G/G	1214:0	G/G
AG/AG	121:0	AG/AG	499:0	AG/AG	287:0	AG/AG	118:0	AG/AG
T/T	121:0	T/T	499:0	T/T	287:0	T/T	118:0	T/T
C/C	402:0	C/C	976:0	C/C	584:0	C/C	454:0	C/C
T/T	956:0	T/T	2963:0	T/T	2115:0	T/T	1417:0	T/T
G/G	956:0	G/G	2963:0	G/G	2115:0	G/G	1417:0	G/G
C/C	956:0	C/C	2963:0	C/C	2115:0	C/C	1417:0	C/C
A/A	142:0	A/A	469:0	A/A	416:0	A/A	263:0	A/A
G/G	142:0	G/G	469:0	G/G	416:0	G/G	263:0	G/G
G/G	1149:0	G/G	1498:0	G/G	1457:0	G/G	1110:0	G/G
C/C	2091:0	C/C	4628:0	C/C	3367:0	C/C	2883:0	C/C

_18B0723	3329_A5_	329_A5_	788_A6_	788_A6_	611_A3_	611_A3_	613_A1_	613_A1_
7508:0	G/G	4003:0	G/G	1673:0	G/G	175:0	G/G	10055:0
7508:0	AGC/CGT	4003:0	AGC/CGT	1673:0	AGC/CGT	175:0	AGC/CGT	10055:0
7508:0	G/G	4003:0	G/G	1673:0	G/G	175:0	G/G	10055:0
7508:0	C/C	4003:0	C/C	1673:0	C/C	175:0	C/C	10055:0
7508:0	C/C	4003:0	C/C	1673:0	C/C	175:0	C/C	10055:0
7508:0	G/G	4003:0	G/G	1673:0	G/G	175:0	G/G	10055:0
7508:0	G/G	4003:0	G/G	1673:0	G/G	175:0	G/G	10055:0
7508:0	G/G	4003:0	G/G	1673:0	G/G	175:0	G/G	10055:0
7508:0	G/G	4003:0	G/G	1673:0	G/G	175:0	G/G	10055:0
7508:0	TTC/GGA	4003:0	TTC/GGA	1673:0	TTC/GGA	175:0	TTC/GGA	10055:0
7508:0	G/G	4003:0	G/G	1673:0	G/G	175:0	G/G	10055:0
7508:0	ACT/ATG	4003:0	AAGACC	388:1043	ACT/ATG	175:0	ACT/ATG	10055:0
7508:0	G/G	4003:0	G/G	1675:0	G/G	175:0	G/G	10055:0
7508:0	G/G	471:0	G/G	1675:0	G/G	97:0	G/G	10055:0
1577:0	G/G	1707:0	G/G	1337:0	G/G	470:0	G/G	3535:0
1577:0	C/C	1707:0	C/C	1337:0	C/C	470:0	C/C	3535:0
1577:0	T/T	1707:0	T/T	1337:0	T/T	470:0	T/T	3535:0
1577:0	-/-	1707:0	-/-	1337:0	-/-	470:0	-/-	3535:0
1577:0	T/T	1707:0	T/T	1337:0	T/T	470:0	T/T	3535:0
1577:0	TGC/ATA	1707:0	TGC/ATA	1337:0	TGC/ATA	470:0	TGC/ATA	3535:0
1577:0	C/C	1707:0	C/C	1337:0	C/C	470:0	C/C	3535:0
1577:0	G/G	1707:0	G/G	1337:0	G/G	472:0	G/G	3535:0
133:0	AG/AG	183:0	AG/AG	221:0	AG/AG	362:0	AG/AG	584:0
133:0	T/T	183:0	T/T	221:0	T/T	362:0	T/T	584:0
511:0	C/C	603:0	C/C	418:0	C/C	276:0	C/C	1251:0
1606:0	T/T	1151:0	T/T	1166:0	T/T	639:0	T/T	2167:0
1606:0	G/G	1151:0	G/G	1166:0	G/G	639:0	G/G	2167:0
1606:0	C/C	1151:0	C/C	1166:0	C/C	639:0	C/C	2167:0
197:0	A/A	195:0	A/A	201:0	A/A	155:0	A/A	562:0
197:0	G/G	195:0	G/G	201:0	G/G	155:0	G/G	562:0
771:0	G/G	817:0	G/G	627:0	G/G	307:0	G/G	2007:0
2358:0	C/C	2137:0	C/C	1603:0	C/C	1084:0	C/C	3709:0

6132_A3_	132_A3_	_18B0723	_18B0723	404_A1_	404_A1_	5717_A3_	717_A3_	7965_A6_
G/G	9318:0	G/-	1239:2016	G/G	213:0	G/G	2609:0	G/G
AGC/CGT	9318:0	AGC/CGT	3540:0	AGC/CGT	213:0	AGC/CGT	2609:0	AGC/CGT
G/G	9318:0	G/G	3540:0	G/G	213:0	G/G	2609:0	G/G
C/C	9318:0	C/C	3540:0	C/C	213:0	C/C	2609:0	C/C
C/C	9318:0	C/C	3540:0	C/C	213:0	C/C	2609:0	C/C
G/G	9318:0	G/G	3540:0	G/G	213:0	G/G	2609:0	G/G
G/G	9318:0	G/G	3540:0	G/G	213:0	G/G	2609:0	G/G
G/G	9318:0	G/G	3540:0	G/G	213:0	G/G	2609:0	G/T
G/G	9318:0	G/G	3540:0	G/G	213:0	G/G	2609:0	G/G
TTC/GGA	9318:0	TTC/GGA	3540:0	TTC/GGA	213:0	TTC/GGA	2609:0	TTC/GGA
G/G	9318:0	G/G	3540:0	G/G	213:0	G/T	904:1389	G/G
ACT/ATG	9318:0	ACT/ATG	3540:0	ACT/ATG	213:0	ACT/ATG	2615:0	ACT/ATG
G/G	9318:0	G/G	3540:0	G/G	213:0	G/G	2615:0	G/G
G/G	9318:0	G/G	3540:0	G/G	213:0	G/G	2615:0	G/G
G/G	3002:0	G/G	2771:0	G/G	1167:0	G/G	1252:0	G/G
C/C	3002:0	C/C	2771:0	C/G	295:960	C/C	1252:0	C/C
T/T	3002:0	T/T	2771:0	T/T	1168:0	T/T	1252:0	T/T
-/-	3002:0	-/-	2771:0	-/-	1168:0	-/-	1252:0	-/-
T/T	3002:0	T/T	2771:0	T/T	1168:0	T/T	1252:0	T/T
TGC/ATA	3002:0	TGC/ATA	2771:0	TGC/ATA	1168:0	TGC/ATA	1252:0	TGC/ATA
C/C	3002:0	C/C	2771:0	C/C	1168:0	C/C	1252:0	C/C
G/G	3008:0	G/G	2777:0	G/G	1168:0	G/G	1252:0	G/G
AG/AG	237:0	AG/AG	187:0	AG/AG	129:0	AG/AG	127:0	AG/AG
T/T	237:0	T/T	187:0	T/T	129:0	T/T	127:0	T/T
C/C	1444:0	C/C	1083:0	C/C	307:0	C/C	418:0	C/C
T/T	2276:0	T/T	1909:0	T/T	1840:0	T/T	801:0	T/T
G/G	2276:0	G/G	1909:0	G/G	1840:0	G/G	801:0	G/G
C/C	2276:0	C/C	1909:0	C/C	1840:0	C/C	801:0	C/C
A/A	278:0	A/A	232:0	A/A	241:0	A/A	151:0	A/A
G/G	278:0	G/G	232:0	G/A	207:20	G/G	151:0	G/G
G/G	2015:0	G/G	1728:0	G/G	1073:0	G/G	759:0	G/G
C/C	3413:0	C/C	3241:0	C/C	3148:0	C/C	1296:0	C/C

7965_A6_	9941_A3_	9941_A3_	5285_A4_	5285_A4_	5953_A2_	5953_A2_	5239_C1_	5239_C1_
5312:0	G/G	961:0	G/G	4549:0	G/G	6368:0	G/G	7122:0
5312:0	AGC/CGT	961:0	AGC/CGT	4549:0	AGC/CGT	6368:0	AGC/CGT	7122:0
5312:0	G/G	961:0	G/G	4549:0	G/G	6368:0	G/G	7122:0
5312:0	C/C	961:0	C/C	4549:0	C/C	6368:0	C/C	7122:0
5312:0	C/C	961:0	C/C	4549:0	C/C	6368:0	C/C	7122:0
5312:0	G/G	961:0	G/G	4549:0	G/G	6368:0	G/G	7122:0
5312:0	G/G	961:0	G/G	4549:0	G/G	6368:0	G/G	7122:0
4249:386	G/G	961:0	G/G	4549:0	G/G	6368:0	G/G	7122:0
5314:0	G/G	961:0	G/G	4549:0	G/G	6368:0	G/G	7122:0
5314:0	TTC/GGA	961:0	TTC/GGA	4549:0	TTC/GGA	6368:0	TTC/GGA	7122:0
5314:0	G/G	961:0	G/G	4549:0	G/G	6368:0	G/T	1039:5144
5314:0	ACT/ATG	961:0	ACT/ATG	4549:0	ACT/ATG	6368:0	ACT/ATG	7136:0
5314:0	G/G	961:0	G/G	4549:0	G/G	6368:0	G/G	7136:0
5314:0	G/G	961:0	G/G	4549:0	G/G	6368:0	G/G	7136:0
2198:0	G/T	780:850	G/G	3137:0	G/G	2282:0	G/G	4386:0
2198:0	C/C	1839:0	C/C	3137:0	C/G	2141:940	C/C	4386:0
2198:0	T/T	1839:0	T/T	3137:0	T/T	2286:0	T/T	4386:0
2198:0	-/-	1839:0	-/-	3137:0	-/-	2286:0	-/-	4386:0
2198:0	T/T	1839:0	T/T	3137:0	T/T	2286:0	T/T	4386:0
2198:0	TGC/ATA	1839:0	TGC/ATA	3137:0	TGC/ATA	2286:0	TGC/ATA	4386:0
2198:0	C/C	1839:0	C/C	3137:0	C/C	2286:0	C/C	4386:0
2203:0	G/G	1839:0	G/G	3137:0	G/G	2286:0	G/G	4402:0
305:0	AG/AG	215:0	AG/AG	292:0	AG/AG	282:0	AG/AG	595:0
305:0	T/T	215:0	T/T	292:0	T/T	282:0	T/T	595:0
937:0	C/C	386:0	C/C	1099:0	C/C	905:0	C/C	942:0
1435:0	T/T	1544:0	T/T	2106:0	T/T	1728:0	T/T	3280:0
1435:0	G/G	1544:0	G/G	2106:0	G/G	1728:0	G/G	3280:0
1435:0	C/C	1544:0	C/C	2106:0	C/C	1728:0	C/C	3280:0
264:0	A/A	208:0	A/A	332:0	A/A	303:0	A/A	582:0
264:0	G/G	208:0	G/G	332:0	G/G	303:0	G/G	582:0
1536:0	G/G	932:0	G/G	1751:0	G/G	1628:0	G/G	1554:0
2253:0	C/C	2923:0	C/C	3167:0	C/C	2377:0	C/C	4325:0

917_B1_g	917_B1_r	495_A1_g	495_A1_r	327_A1_g	327_A1_r	011_A4_g	011_A4_r	182_A5_g
G/G	57:0	G/G	61:0	G/G	89:0	G/G	57:0	G/G
AGC/CGT	57:0	AGC/CGT	61:0	AGC/CGT	89:0	AGC/CGT	57:0	AGC/CGT
G/G	57:0	G/G	61:0	G/G	89:0	G/G	57:0	G/G
C/C	57:0	C/C	61:0	C/C	89:0	C/C	57:0	C/C
C/C	57:0	C/C	61:0	C/C	89:0	C/C	57:0	C/C
G/G	57:0	G/G	61:0	G/G	89:0	G/G	57:0	G/G
G/G	57:0	G/G	61:0	G/G	89:0	G/G	57:0	G/G
G/G	57:0	G/G	61:0	G/G	89:0	G/G	57:0	G/G
G/G	58:0	G/G	61:0	G/G	89:0	G/G	57:0	G/G
TTC/GGA	58:0	TTC/GGA	61:0	TTC/GGA	89:0	TTC/GGA	57:0	TTC/GGA
G/G	58:0	G/G	61:0	G/G	89:0	G/G	57:0	G/G
ACT/ATG	58:0	ACT/ATG	61:0	ACT/ATG	89:0	ACT/ATG	58:0	ACT/ATG
G/G	58:0	G/G	61:0	G/G	89:0	G/G	58:0	G/G
G/G	58:0	G/G	61:0	G/G	89:0	G/G	58:0	G/G
G/G	669:0	G/G	349:0	G/G	2308:0			G/G
C/C	669:0	C/C	349:0	C/C	2308:0			C/C
T/T	669:0	T/T	349:0	T/T	2308:0			T/T
-/-	669:0	-/-	349:0	-/-	2308:0	-/-	211:0	-/-
T/T	669:0	T/T	349:0	T/T	2308:0	T/T	215:0	T/T
TGC/ATA	669:0	TGC/ATA	349:0	TGC/ATA	2308:0	TGC/ATA	215:0	TGC/ATA
C/C	669:0	C/C	349:0	C/C	2308:0	C/C	166:0	C/C
G/G	669:0	G/G	349:0	G/G	2308:0	G/G	166:0	G/G
AG/AG	37:0			AG/AG	87:0	AG/AG	228:0	AG/AG
T/T	37:0			T/T	87:0	T/T	228:0	T/T
C/C	56:0	C/C	138:0	C/C	588:0	C/C	22:0	C/C
T/T	258:0	T/T	651:0	T/T	1702:0	T/T	885:0	T/T
G/G	258:0	G/G	651:0	G/G	1702:0	G/G	885:0	G/G
C/T	181:73	C/C	651:0	C/C	1702:0	C/C	885:0	C/C
A/A	99:0	A/A	52:0	A/A	131:0	A/A	43:0	A/A
G/G	99:0	G/G	35:0	G/G	131:0	G/G	43:0	G/G
G/G	69:0	G/G	101:0	G/G	785:0	G/A	23:4	G/G
C/C	651:0	C/C	584:0	C/C	3069:0	C/C	1597:0	C/C

182_A5_r	864_A1_g	864_A1_r	7432_A_g	7432_A_re	396_A2_g	396_A2_r	415_A2_g	415_A2_r
233:0	G/G	880:0	G/G	523:0	G/G	1534:0	G/G	274:0
233:0	AGC/CGT	880:0	AGC/CGT	523:0	AGC/CGT	1534:0	AGC/CGT	274:0
233:0	G/G	880:0	G/G	523:0	G/G	1534:0	G/G	274:0
233:0	C/C	880:0	C/C	523:0	C/C	1534:0	C/C	274:0
233:0	C/C	880:0	C/C	523:0	C/C	1534:0	C/C	274:0
233:0	G/G	880:0	G/G	523:0	G/-	1921:728	G/-	351:137
233:0	G/G	880:0	G/G	523:0	G/G	1530:0	G/G	273:0
233:0	G/G	880:0	G/G	523:0	G/G	1530:0	G/G	273:0
233:0	G/G	880:0	G/G	523:0	G/G	1530:0	G/G	273:0
233:0	TTC/GGA	880:0	TTC/GGA	523:0	TTC/GGA	1530:0	TTC/GGA	273:0
233:0	G/G	880:0	G/G	523:0	G/G	1530:0	G/G	273:0
233:0	ACT/ATG	880:0	ACT/ATG	523:0	ACT/ATG	1530:0	ACT/ATG	273:0
233:0	G/G	880:0	G/G	523:0	G/G	1530:0	G/G	273:0
233:0	G/G	880:0	G/G	523:0	G/G	1530:0	G/G	273:0
2543:0	G/G	5051:0	G/G	2590:0	G/G	2524:0	G/G	1584:0
2543:0	C/C	5051:0	C/C	2590:0	C/C	2524:0	C/C	1584:0
2543:0	T/T	5051:0	T/T	2590:0	T/T	2524:0	T/T	1584:0
2543:0	-/-	5051:0	-/-	2590:0	-/-	2524:0	-/-	1584:0
2543:0	T/T	5051:0	T/T	2590:0	T/T	2524:0	T/T	1584:0
2543:0	TGC/ATA	5051:0	TGC/ATA	2590:0	TGC/ATA	2524:0	TGC/ATA	1584:0
2543:0	C/C	5051:0	C/C	2590:0	C/C	2524:0	C/C	1584:0
2543:0	G/G	5051:0	G/G	2590:0	G/G	2524:0	G/G	1584:0
112:0	AG/AG	1202:0	AG/AG	345:0	AG/-	160:215	AG/-	93:172
112:0	T/T	1202:0	T/C	97:223	T/T	402:0	T/T	287:0
455:0			C/C	710:0	C/C	998:0	C/C	764:0
2385:0	T/T	3256:0	T/T	1771:0	T/T	2208:0	T/T	1134:0
2385:0	G/G	3256:0	G/G	1771:0	G/G	2208:0	G/G	1134:0
2385:0	C/C	3256:0	C/C	1771:0	C/C	2208:0	C/C	1134:0
176:0	A/A	862:0	A/A	437:0	A/A	393:0	A/A	249:0
176:0	G/G	862:0	G/G	437:0	G/G	393:0	G/G	249:0
681:0			G/G	1094:0	G/G	1064:0	G/G	764:0
2993:0	C/C	4294:0	C/C	2687:0	C/C	3351:0	C/C	1924:0

912_B2_g	912_B2_r	517_C2_g	517_C2_r	05746_ge	05746_rea	08008_ge	08008_rea	08625_ge
				C/C	2883:0	C/C	2082:0	C/C
				G/G	1622:0	G/G	1001:0	G/G
				C/C	45:0	C/C	23:0	C/C
G/G	6354:0	G/G	2066:0					
AGC/CGT	6354:0	AGC/CGT	2066:0					
G/G	6354:0	G/G	2066:0					
C/C	6354:0	C/C	2066:0					
C/C	6354:0	C/C	2066:0					
G/G	6354:0	G/G	2066:0					
G/G	6354:0	G/G	2066:0					
G/G	6354:0	G/G	2066:0					
G/G	6354:0	G/G	2066:0					
TTC/GGA	6354:0	TTC/GGA	2066:0					
G/G	6354:0	G/G	2066:0					
ACT/ATG	6354:0	ACT/ATG	2066:0					
G/G	6354:0	G/G	2066:0					
G/G	6354:0	G/G	2066:0					
G/G	3491:0	G/G	3154:0					
C/C	3491:0	C/G	3547:749					
T/T	3491:0	T/T	3156:0					
-/-	3491:0	-/-	3156:0					
T/T	3491:0	T/T	3156:0					
TGC/ATA	3491:0	TGC/ATA	3156:0					
C/C	3491:0	C/C	3156:0					
G/G	3491:0	G/G	3156:0					
AG/AG	817:0	AG/AG	281:0					
T/T	817:0	T/T	281:0					
C/C	684:0	C/C	921:0					
T/T	2554:0	T/T	2794:0					
G/G	2554:0	G/G	2794:0					
C/C	2554:0	C/C	2794:0					
A/A	595:0	A/A	293:0					
G/G	595:0	G/G	293:0					
G/G	865:0	G/G	1571:0					
C/C	4321:0	C/C	4507:0					
				G/G	118:0	G/G	89:0	G/G
				C/C	813:0	C/C	465:0	C/C
				C/C	813:0	C/C	465:0	C/C
				G/G	818:0	G/G	582:0	G/G
				CCC/GCA	204:0	CCC/GCA	92:0	CCC/GCA
				C/C	28:0	C/C	26:0	C/C
				C/C	319:0	C/C	152:0	C/C
				G/G	287:0	G/G	182:0	G/G

				C/C	924:0	C/C	667:0	C/C
				A/A	240:0	A/A	182:0	A/A
				G/G	805:0	G/G	154:0	G/G
				A/A	72:0	A/A	42:0	A/A

08625_rea	37503_ge	37503_rea	46897_ge	46897_rea	48763_ge	48763_rea	71646_ge	71646_rea
3256:0	C/C	3210:0	C/C	2711:0	C/C	2654:0	C/C	2256:0
1195:0	G/G	1066:0	G/G	1069:0	G/G	471:0	G/G	2392:0
35:0	C/C	41:0	C/C	30:0	C/C	15:0	C/C	93:0
352:0	G/G	328:0	G/G	270:0	G/G	455:0	G/G	100:0
695:0	C/C	841:0	C/C	816:0	C/C	266:0	C/C	739:0
695:0	C/C	841:0	C/C	816:0	C/C	266:0	C/C	739:0
446:0	G/G	772:0	G/G	556:0	G/G	134:0	G/G	660:0
39:0	CCC/GCA	87:0	CCC/GCA	59:0	CCC/GCA	254:0	CCC/GCA	132:0
41:0	C/C	53:0	C/C	295:0	C/C	368:0	C/C	21:0
145:0	C/C	240:0	C/C	138:0	C/C	62:0	C/C	213:0
266:0	G/G	692:0	G/G	601:0	G/G	541:0	G/G	169:0

894:0	C/C	931:0	C/C	800:0	C/C	782:0	C/C	993:0
265:0	A/A	927:0	A/A	7655:0	A/A	6968:0	A/A	169:0
100:0	G/G	210:0	G/G	48:0	G/G	31:0	G/G	1374:0
118:0	A/T	128:19	A/A	81:0	A/A	39:0	A/A	98:0

)76989_ge	76989_rea	77285_ge	77285_rea	79054_ge	79054_rea	08329_ge	08329_rea	10788_ge
C/C	2789:0	C/C	4025:0	C/C	2450:0	C/C	2952:0	C/C
G/G	829:0	G/G	730:0	G/G	1896:0	G/G	1994:0	G/G
C/C	49:0	C/C	9:0	C/C	122:0	C/C	157:0	C/C
G/G	386:0	G/G	144:0	G/G	352:0	G/G	257:0	G/G
C/C	550:0	C/C	622:0	C/C	788:0	C/C	1177:0	C/C
C/C	550:0	C/C	622:0	C/C	788:0	C/C	1177:0	C/C
G/G	312:0	G/G	395:0	G/G	621:0	G/G	1536:0	G/G
CCC/GCA	56:0	CCC/GCA	50:0	CCC/GCA	63:0	CCC/GCA	275:0	CCC/GCA
C/C	58:0	C/C	21:0	C/C	38:0	C/C	42:0	C/C
C/C	108:0	C/C	112:0	C/C	239:0	C/C	643:0	C/C
G/G	892:0	G/G	775:0	G/G	244:0	G/G	250:0	G/G

C/C	668:0	C/C	1093:0	C/C	849:0	C/C	806:0	C/C
A/A	8087:0	A/A	1086:0	A/A	845:0	A/A	249:0	A/A
G/G	29:0	G/G	252:0			G/G	13:0	G/G
A/A	103:0	A/A	37:0	A/A	114:0	A/A	170:0	A/A

10788_rea	17611_ge	17611_rea	17613_ge	17613_rea	26132_ge	26132_rea	31735_ge	31735_rea
3613:0	C/C	1308:0	C/C	2153:0	C/C	2136:0	C/C	2860:0
1280:0	G/G	233:0	G/G	1410:0	G/G	565:0	G/G	699:0
47:0	C/C	6:0	C/C	74:0	C/C	18:0	C/C	15:0
186:0	G/G	611:0	G/G	378:0	G/G	264:0	G/G	324:0
580:0	C/C	72:0	C/C	1065:0	C/C	238:0	C/C	580:0
580:0	C/C	72:0	C/C	1065:0	C/C	238:0	C/C	580:0
513:0	G/G	23:0	G/G	1137:0	G/G	234:0	G/G	583:0
113:0	CCC/GCA	145:0	CCC/GCA	71:0	CCC/GCA	34:0	CCC/GCA	62:0
221:0	C/C	332:0	C/C	435:0	C/C	267:0	C/C	644:0
161:0	C/C	15:0	C/C	309:0	C/C	70:0	C/C	163:0
451:0	G/G	600:0	G/G	619:0	G/G	629:0	G/G	560:0

1058:0	C/C	588:0	C/C	879:0	C/C	1109:0	C/C	1046:0
77:0	A/A	5098:0	A/A	6831:0	A/A	1103:0	A/A	292:0
59:0			G/G	10:0	G/G	26:0	G/G	203:0
53:0	A/A	30:0	A/A	112:0	A/A	43:0	A/A	64:0

40404_ge	40404_rea	55717_ge	55717_rea	77965_ge	77965_rea	79941_ge	79941_rea	05285_ge
C/C	2762:0	C/C	3373:0	C/C	2538:0	C/C	2295:0	C/C
G/G	561:0	G/G	1660:0	G/G	1323:0	G/G	335:0	G/G
		C/C	84:0	C/C	56:0	C/C	12:0	C/C
G/G	218:0	G/G	213:0	G/G	212:0	G/G	447:0	G/G
C/C	622:0	C/C	1119:0	C/C	849:0	C/C	565:0	C/C
C/C	622:0	C/C	1119:0	C/C	849:0	C/C	565:0	C/C
G/G	375:0	G/G	1037:0	G/G	929:0	G/G	164:0	G/G
CCC/GCA	72:0	CCC/GCA	174:0	CCC/GCA	127:0	CCC/GCA	34:0	CCC/GCA
C/C	18:0	C/C	379:0	C/C	705:0	C/C	201:0	C/C
C/C	153:0	C/C	646:0	C/C	333:0	C/C	74:0	C/C
G/G	623:0	G/G	132:0	G/G	292:0	G/G	238:0	G/G

C/C	914:0	C/C	1176:0	C/C	952:0	C/C	785:0	C/C
A/A	68:0	A/A	132:0	A/A	437:0	A/A	8173:0	A/A
G/G	55:0	G/G	90:0	G/G	486:0	G/G	156:0	G/G
A/A	28:0	A/A	93:0	A/A	95:0	A/A	44:0	A/A

05285_rea	25953_ge	25953_rea	86239_ge	86239_rea	97327_gen	97327_rea	23733_gen	23733_rea
2269:0	C/C	1772:0	C/C	998:0	C/C	1232:0	C/C	2079:0
1665:0	G/G	1078:0	G/G	1290:0	G/G	496:0	G/G	109:0
82:0	C/C	77:0	C/C	70:0	C/C	5:0		
107:0	G/G	527:0	G/G	101:0	G/G	59:0	G/G	549:0
932:0	C/C	848:0	C/C	1055:0	C/C	121:0	C/C	116:0
932:0	C/C	848:0	C/C	1055:0	C/C	121:0	C/C	116:0
1099:0	G/G	724:0	G/G	1130:0	G/G	196:0	G/G	34:0
273:0	CCC/GCA	44:0	CCC/GCA	88:0	CCC/GCA	39:0	CCC/GCA	112:0
22:0	C/C	44:0	C/C	73:0	C/C	26:0	C/C	425:0
319:0	C/C	251:0	C/C	374:0	C/C	72:0	C/C	10:0
244:0	G/G	191:0	G/G	421:0	G/G	68:0	G/G	262:0

835:0	C/C	535:0	C/C	559:0	C/C	397:0	C/C	262:0
244:0	A/A	6299:0	A/A	7517:0	A/A	71:0	A/A	3948:0
1057:0			G/G	10:0	G/G	94:0		
84:0	A/A	120:0	A/A	134:0	A/T	6:18	A/A	38:0

29182_ger	29182_rea	97864_ger	97864_rea	76396_ger	76396_rea	76415_ger	76415_rea	43912_ger
C/C	2600:0	C/C	403:0	C/C	1640:0	C/C	1553:0	C/C
G/G	1378:0	G/G	130:0	G/G	321:0	G/G	231:0	G/G
C/C	35:0	C/C	21:0	C/C	10:0			C/C
G/G	72:0	G/G	123:0	G/G	303:0	G/G	578:0	G/G
C/C	433:0	C/C	27:0	C/C	169:0	C/C	114:0	C/C
C/C	433:0	C/C	27:0	C/C	169:0	C/C	114:0	C/C
G/G	494:0	G/G	21:0	G/G	93:0	G/G	31:0	G/G
CCC/GCA	119:0	CCC/GCA	38:0	CCC/GCA	108:0	CCC/GCA	74:0	CCC/GCA
C/C	20:0			C/C	53:0	C/C	115:0	C/C
C/C	240:0	C/C	12:0	C/C	51:0	C/C	25:0	C/C
G/G	354:0	G/G	83:0	G/G	185:0	G/G	247:0	G/G

C/C	976:0	C/C	183:0	C/C	838:0	C/C	717:0	C/C
A/A	173:0	A/A	795:0	A/G	4067:547	A/A	3263:0	A/A
G/G	484:0	G/G	11:0	G/G	15:0	G/A	9:5	G/G
A/A	37:0	A/A	46:0	A/A	34:0	A/A	41:0	A/A

43912_rea	65517_ge	65517_reads
2048:0	C/C	3032:0
576:0	G/G	842:0
9:0	C/C	13:0
76:0	G/G	177:0
373:0	C/C	485:0
373:0	C/C	485:0
416:0	G/G	438:0
53:0	CCC/GCA	51:0
29:0	C/C	23:0
304:0	C/C	128:0
126:0	G/G	220:0

702:0	C/C	1012:0	
126:0	A/A	220:0	
403:0	G/G	445:0	
34:0	A/A	54:0	
