# Supplementary material for: Genetic Alteration Profiles and Clinicopathological Associations in Atypical Parathyroid Adenoma
Source: Int J Genomics. 2021 Mar 9;2021:6666257. doi: 10.1155/2021/6666257 (PMC7969847; doi:10.1155/2021/6666257)
Supplement: Supplementary 1 — Supplemental Figure 1 Sanger sequencing results of all alterations in the parathyroid tumor samples. [file 6666257.f1.zip › Supplemental Table 3 (1).docx]

Supplemental Table 3 Clinical features of patients with PC who comprised the study cohort with the genomic variants called, reference sequences and SNP-ID

| ID | Tumor Size | Serum Ca(mmol/L)  (2.13-2.70mmol/L) | PTH(pg/ml)  (12.0-65.0pg/ml) | Gene | cDNA change | Protein change | Reference  sequence | | SNP-ID |  |
| --- | --- | --- | --- | --- | --- | --- | --- | --- | --- | --- |
| 001  003 | 2.1  1.8 | 2.05  2.88 | 387  865 | CDC73  EZH2 | c.664C>T  c.1936T>A | p.Arg222*  p.Tyr646Asn | NM_024529  NM_004456 | rs267601395 | | |
| 004 | 2 | 2.62 | 1896 | CDC73 | c.1305G>A | p.Met435Ile | NM_024529 |  | | |
| 007 | 1.1 | 2.76 | 178 | CDC73 | c.293T>C | p.Leu98Pro | NM_024529 |  | | |
| 008 | 2 | 3.37 | 69.3 | CDC73  HIC1 | c.45delG  c.1571A>G | p.Lys16Argfs*5  p.Lys524Arg | NM_024529  NM_006497 |  | | |
| 009  011  013  015 | 0.8  1.8  3  2.6 | 2.5  3.52  2.9  3.5 | 159  180  325  406 | CDC73  HIC1  CDC73  CDC73  EZH2 | c.45delG  c.2002G>A  c.162C>G  c.162C>G  c.1936T>A | p.Lys16Argfs*5  p.Ala668Thr  p.Tyr54*  p.Tyr54*  p.Tyr646Asn | NM_024529  NM_006497  NM_024529  NM_024529  NM_004456 | rs991141113  rs121434265  rs121434265  rs267601395 | | |
| 016 | 4 | 3.3 | 418 | CDC73 | c.18delC | p.Ser6Argfs*15 | NM_024529 |  | | |
| 018  021 | 3.6  2.0 | 3.4  3.1 | 1232  405 | CDC73  CDC73 | c.162C>G  c.108_122delATGTGAAGACCAACT | p.Tyr54*  p.Val36del | NM_024529  NM_024529 | rs121434265 | | |
| 025  026 | 1.5  2.2 | 2.7  2.8 | 589  309 | CDC73  CDC73  CDC73 | c.4delG  c.817G>A  c.162C>G | p.Ala2Argfs*19  p.Ala273Thr  p.Tyr54* | NM_024529  NM_024529 | rs1060500020  rs121434265 | | |
| 027 | 2.2 | 2.9 | 312 | CDC73 | c.85G>T | p.Glu29* | NM_024529 |  |  |  |
| 028 | 1.8 | 3.8 | 1821 | CDC73 | c.70G>T | p.Glu24* | NM_024529 |  | | |
| 029 | 2.2 | 2.6 | 1402 | CDC73 | c.157G>T | p.Glu53* | NM_024529 |  | | |
| 031 | 2.8 | 1.4 | 1299 | CDC73 | c.162C>G | p.Tyr54* | NM_024529 | rs121434265 | | |
| 032 | 3 | 3.2 | 875 | CDC73 | c.85G>T | p.Glu29* | NM_024529 |  | | |
| 034 | 3.2 | 2.6 | 2011 | CDC73 | c.162C>G | p.Tyr54* | NM_024529 | rs121434265 | | |

ID 028：HPT-JT syndrome case
