# Supplementary material for: Genetic Alteration Profiles and Clinicopathological Associations in Atypical Parathyroid Adenoma
Source: Int J Genomics. 2021 Mar 9;2021:6666257. doi: 10.1155/2021/6666257 (PMC7969847; doi:10.1155/2021/6666257)
Supplement: Supplementary 1 — Supplemental Figure 1 Sanger sequencing results of all alterations in the parathyroid tumor samples. [file 6666257.f1.zip › Supplemental Table 4 (1).docx]

Supplemental Table 4 Clinical features of patients with PA who comprised the study cohort with the genomic variants called, reference sequences and SNP-ID

| ID | Tumor Size | Serum Ca(mmol/L)  (2.13-2.70mmol/L) | PTH(pg/ml)  (12.0-65.0pg/ml) | Gene | cDNA change | Protein change | Reference  sequence | SNP-ID |
| --- | --- | --- | --- | --- | --- | --- | --- | --- |
| 087 | 2.2 | 3.51 | 954 | HIC1 | c.1571A>G | p.Lys524Arg | NM_006497 |  |
| 092 | 1 | 3.18 | 340 | CDC73 | c.64G>T | p.Gly22* | NM_024529 |  |
| 097 | 1.5 | 1.18 | 226 | MEN1 | c.1360G>T | p.Gly454* | NM_000244 |  |
| 102 | 3.8 | 2.83 | 1039 | RASSF1 | c.599G>A | p.Arg200His | NM_007182 | rs761354693 |
| 103  108 | 1  1.6 | 3.34  2.2 | 192  225 | CDKN1B  CDC73  CDC73 | c.285delC  c.162C>G  c.226C>T | p.Gly97Valfs*22  p.Tyr54*  p.Arg76* | NM_004064  NM_024529  NM_024529 | rs121434265  rs886041158 |
| 109 | 1.5 | 1.31 | 188 | CDC73 | c.131+1G>C | - | NM_024529 |  |
| 112 | 1.8 | 1.06 | 186 | MEN1 | c.1060C>T | p.Gln354* | NM_000244 |  |
|  |  |  |  | CDKN2A | c.343G>T | p.Val115Leu |  |  |
| 118 | 1 | 2.43 | 991 | CDC73 | c.163T>A | p.Tyr55Asn | NM_024529. |  |
| 121 | 3.5 | 2.68 | 509 | MEN1 | c.503_510delGGGCCTGC | p.Gly168Alafs*14 | NM_000244 |  |
| 134 | 2 | 3.57 | 143 | MEN1 | c.1493C>T  c.133G>T | p.Pro498Leu  p.Glu45* | NM_000244  NM_000244 | rs766604600 |
