# Supplementary material for: Genetic Alteration Profiles and Clinicopathological Associations in Atypical Parathyroid Adenoma
Source: Int J Genomics. 2021 Mar 9;2021:6666257. doi: 10.1155/2021/6666257 (PMC7969847; doi:10.1155/2021/6666257)

Supplemental Fig.1 Sanger sequencing results of all alterations in pararthyroid tumors

| CDC73 | 193091333-193091333 | G | - |
| --- | --- | --- | --- |


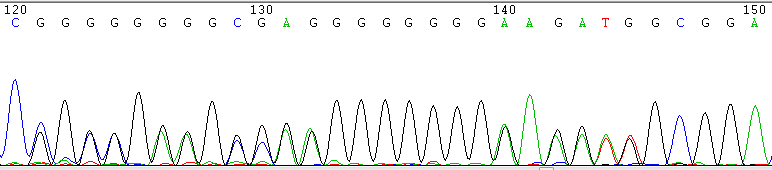


| CDC73 | 193091339-193091348 | CGTGCTTAGC | - |
| --- | --- | --- | --- |


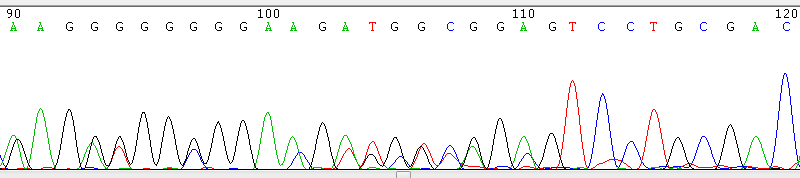


| CDC73 | 193091340-193091340 | G | - |
| --- | --- | --- | --- |


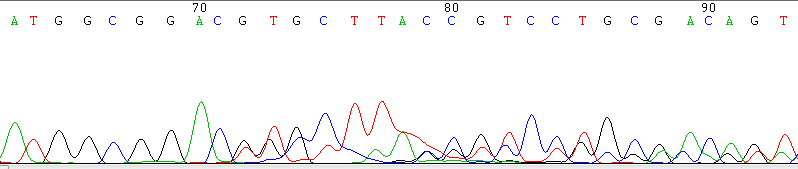


| CDC73 | 193091348-193091348 | C | - |
| --- | --- | --- | --- |


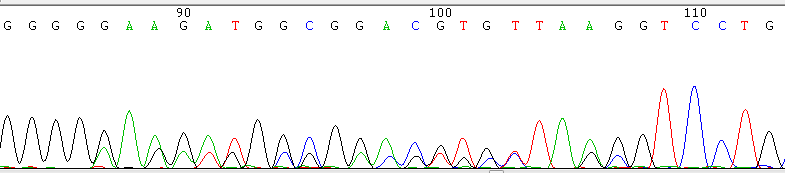


| CDC73 | 193091355 | C | T |
| --- | --- | --- | --- |


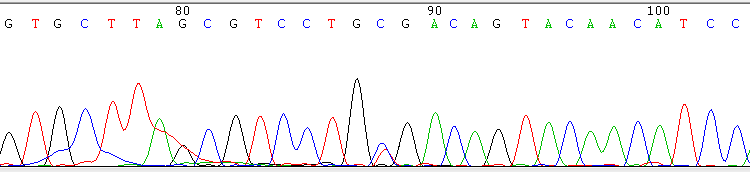


| CDC73 | 193091375-193091375 | G | - |
| --- | --- | --- | --- |


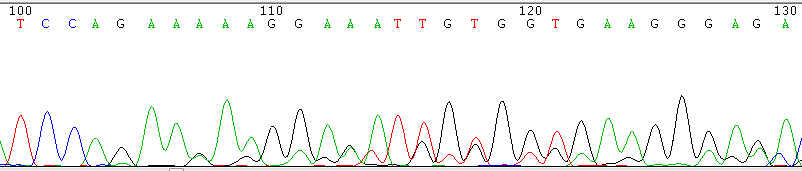


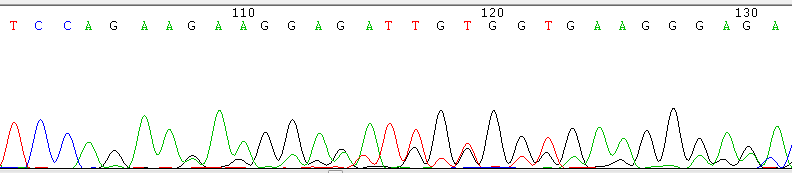


| CDC73 | 193091394 | G | T |
| --- | --- | --- | --- |


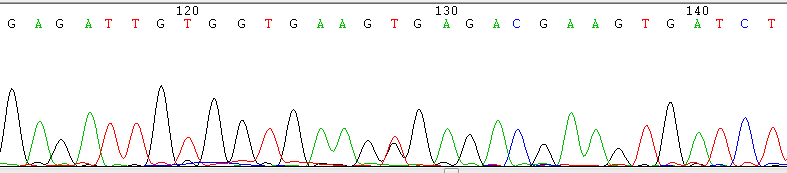


| CDC73 | 193091400 | G | T |
| --- | --- | --- | --- |


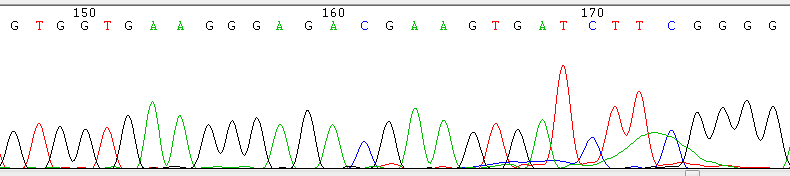


| CDC73 | 193091412-193091412 | G | - |
| --- | --- | --- | --- |


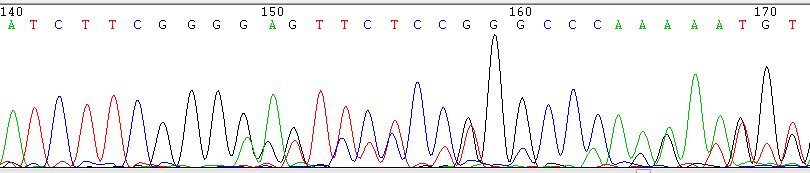


| CDC73 | 193091414-193091420 | GGAGTTC | - |
| --- | --- | --- | --- |


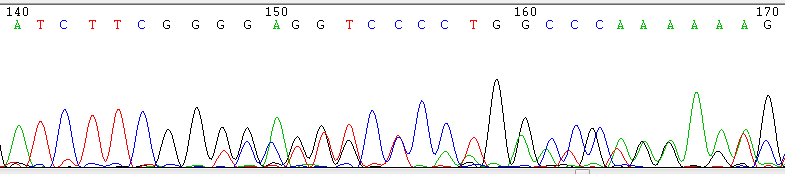


| CDC73 | 193091415 | G | T |
| --- | --- | --- | --- |


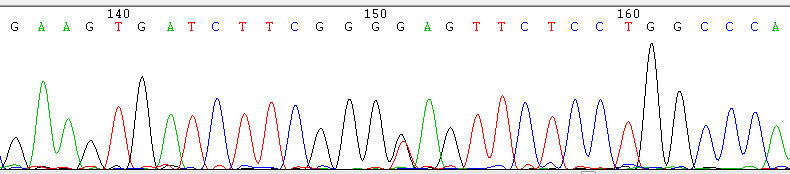


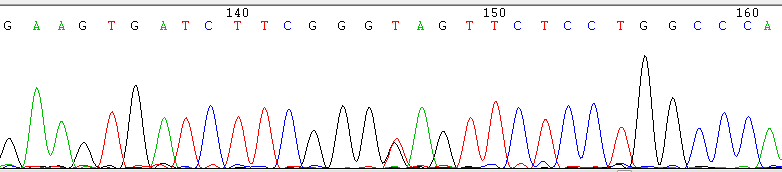


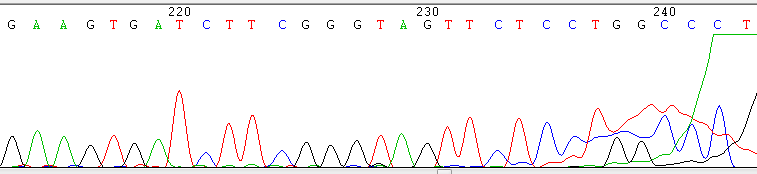


| CDC73 | 193091434-193091448 | ATGTGAAGACCAACT | - |
| --- | --- | --- | --- |


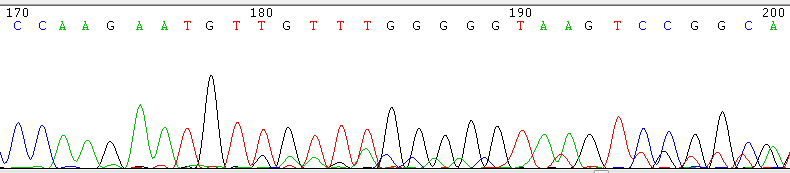


| CDC73 | 193091458 | G | A |
| --- | --- | --- | --- |


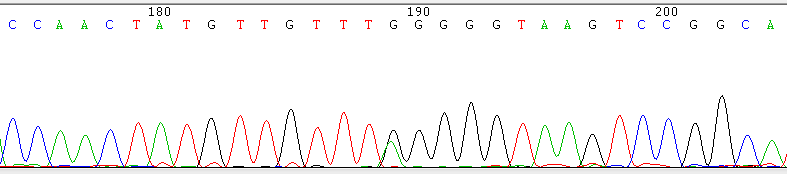


| CDC73 | 193091462 | G | A/C |
| --- | --- | --- | --- |


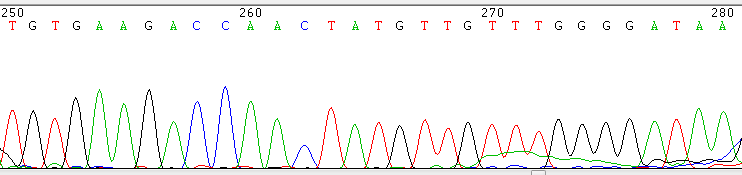


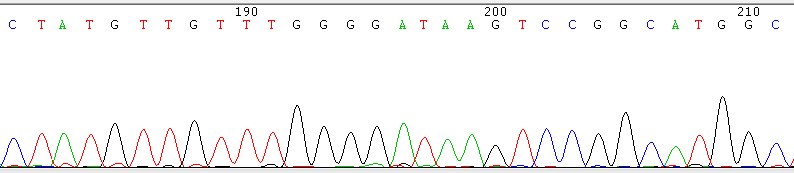


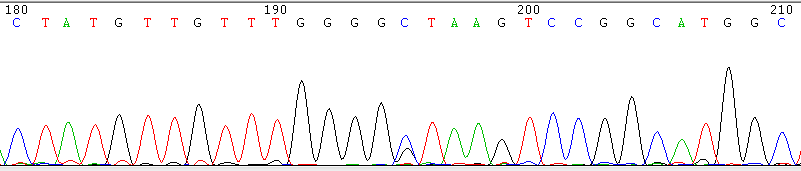


| CDC73 | 193094267 | G | T |
| --- | --- | --- | --- |


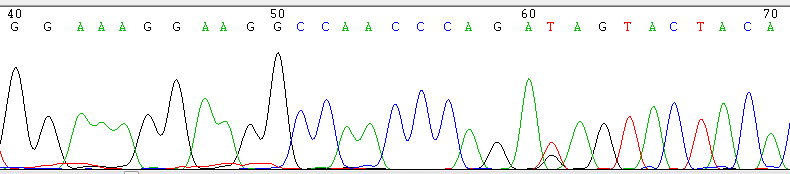


| CDC73 | 193094272 | C | G |
| --- | --- | --- | --- |


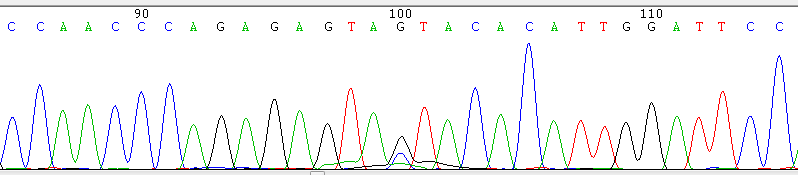


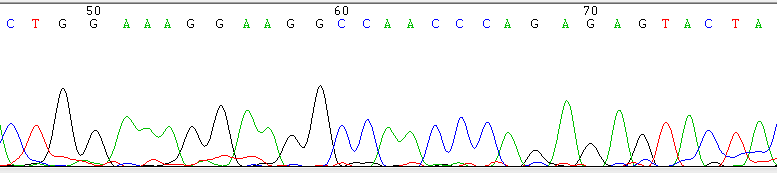


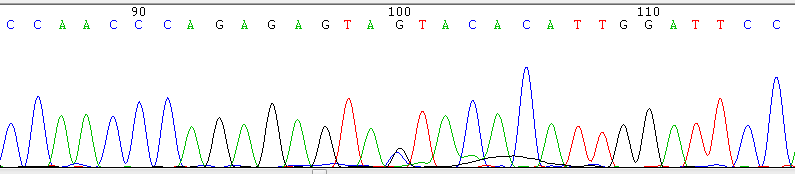


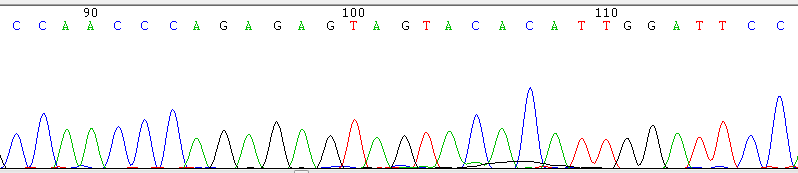


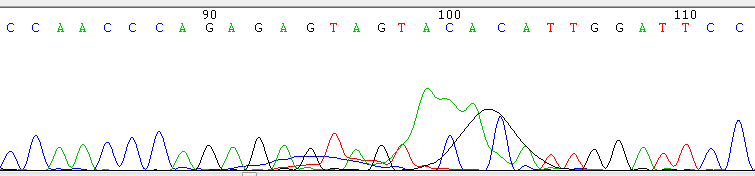


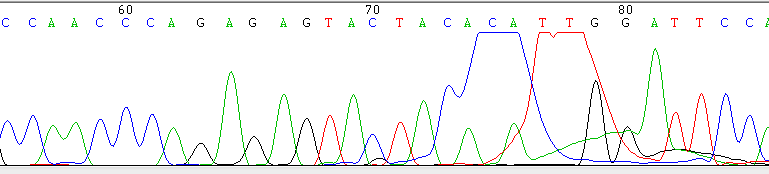


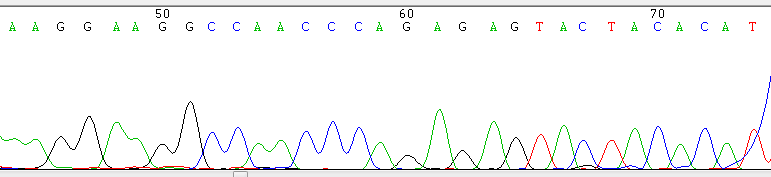


| CDC73 | 193094273 | T | A |
| --- | --- | --- | --- |


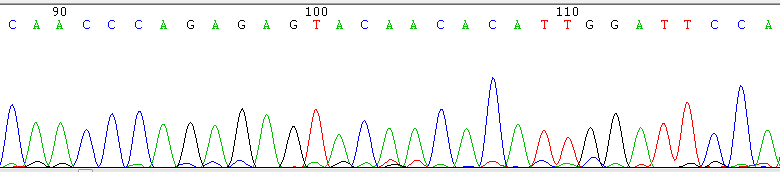


| CDC73 | 193094283-193094283 | - | T |
| --- | --- | --- | --- |


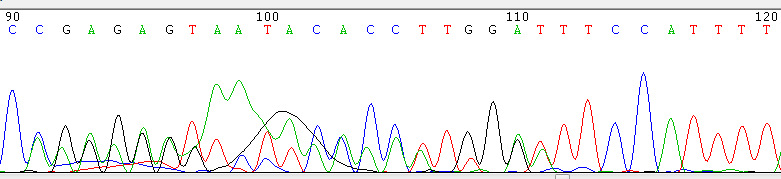


| CDC73 | 193094301 | T | C |
| --- | --- | --- | --- |


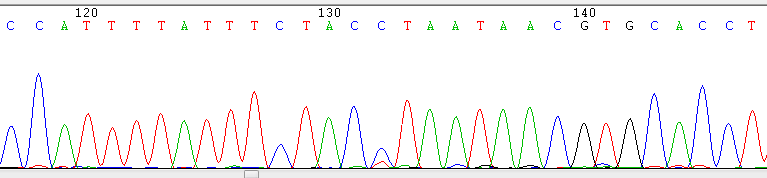


| CDC73 | 193094304-193094312 | ATAACGTGC | - |
| --- | --- | --- | --- |


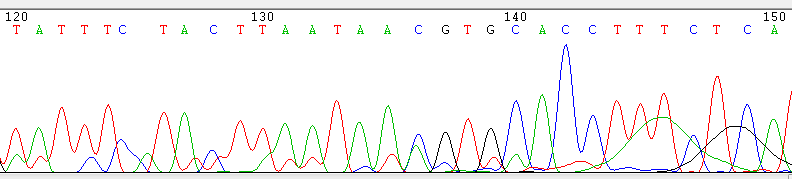


| CDC73 | 193094336 | C | T |
| --- | --- | --- | --- |


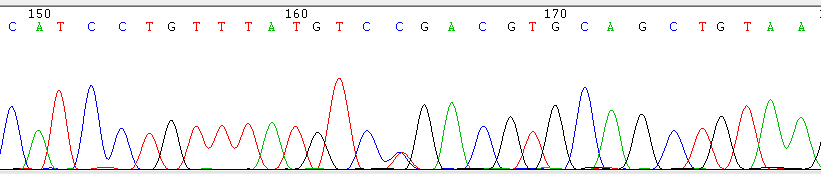


| CDC73 | 193094342 | G | C |
| --- | --- | --- | --- |


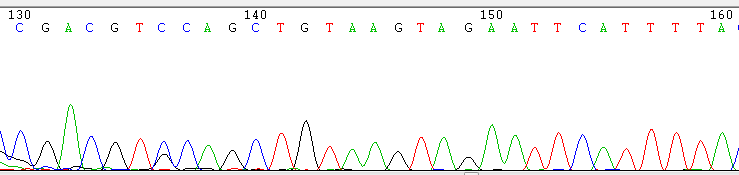


| CDC73 | 193099325-193099326 | AG | - |
| --- | --- | --- | --- |


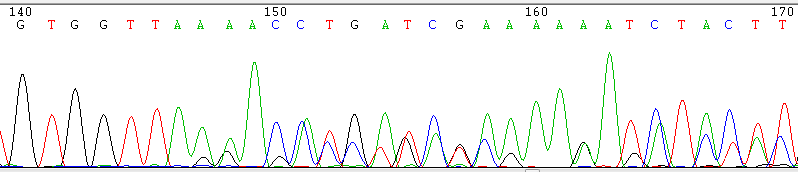


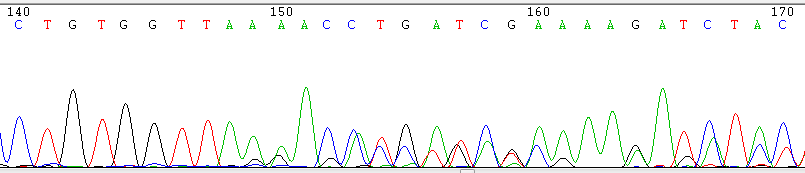


| CDC73 | 193099359 | T | C |
| --- | --- | --- | --- |


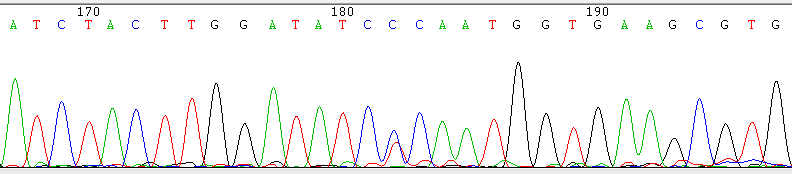


| CDC73 | 193104575 | C | T |
| --- | --- | --- | --- |


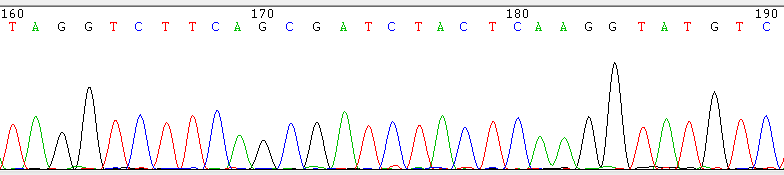


| CDC73 | 193111016-193111016 | T | - |
| --- | --- | --- | --- |


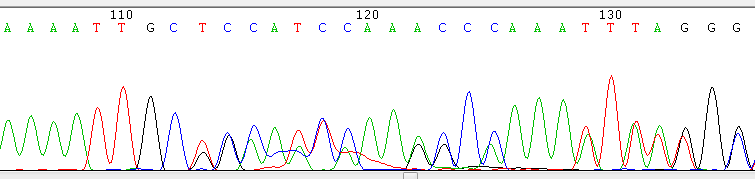


| CDC73 | 193111037-193111037 | G | - |
| --- | --- | --- | --- |


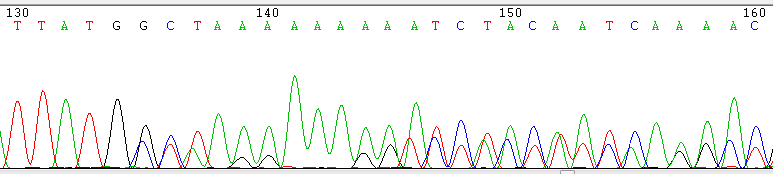


| CDC73 | 193111131 | C | T |
| --- | --- | --- | --- |


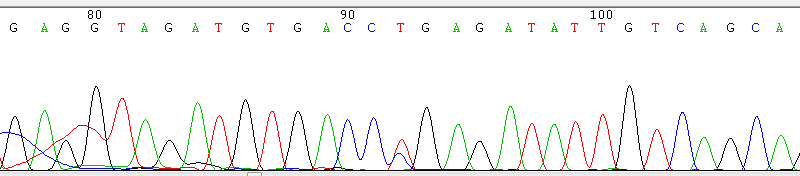


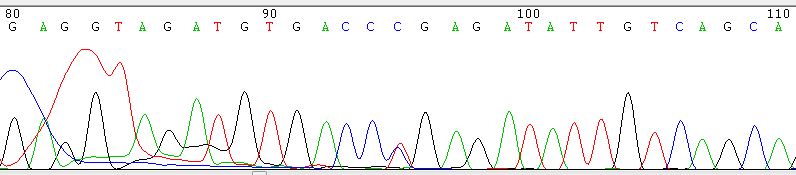


| CDC73 | 193117020-193117020 | A | - |
| --- | --- | --- | --- |


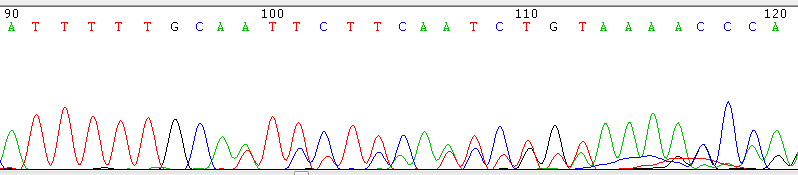


| CDC73 | 193117084 | G | A |
| --- | --- | --- | --- |


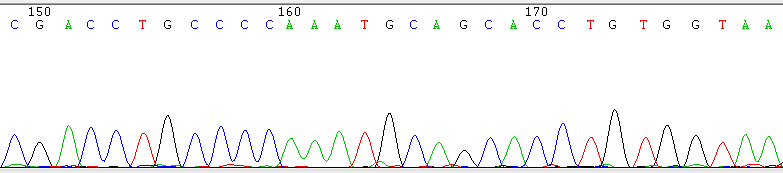


| CDC73 | 193202273 | G | A |
| --- | --- | --- | --- |


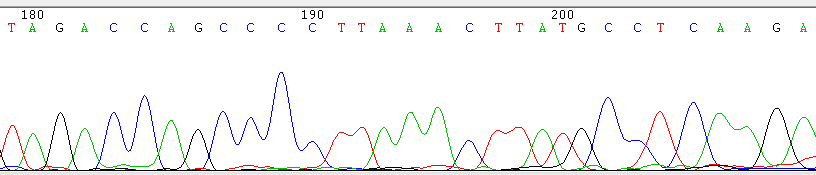


| CDC73 | 193205463 | C | A |
| --- | --- | --- | --- |


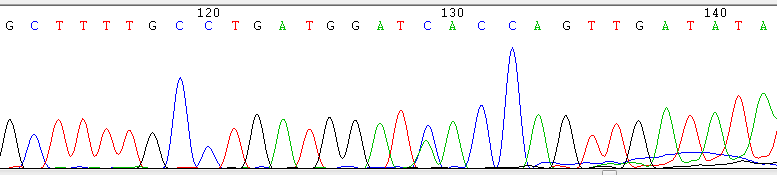


| RASSF1 | 50369151 | G | A |
| --- | --- | --- | --- |


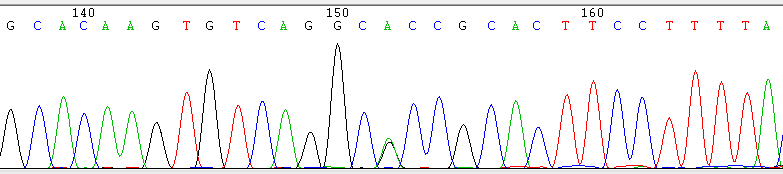


| RASSF1 | 50369178 | G | A |
| --- | --- | --- | --- |


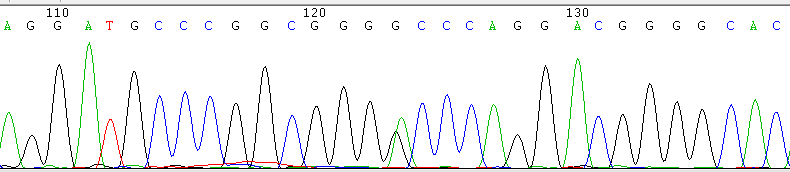


| EZH2 | 148508728 | T | A |
| --- | --- | --- | --- |


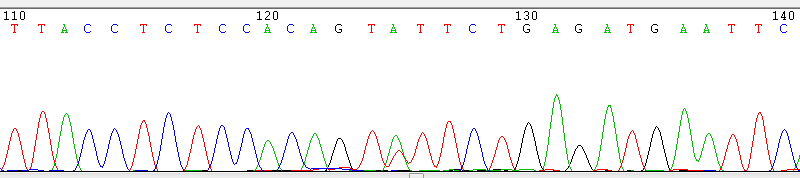


**
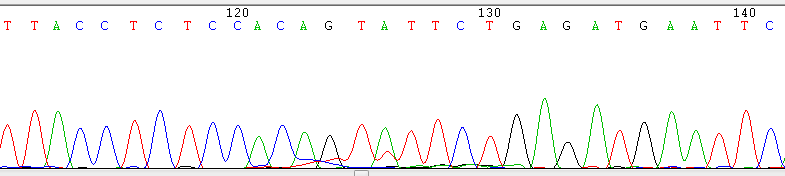
**

| EZH2 | 148513830 | C | A |
| --- | --- | --- | --- |


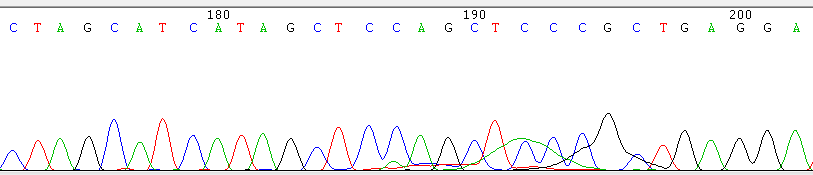


| EZH2 | 148524337 | G | A |
| --- | --- | --- | --- |


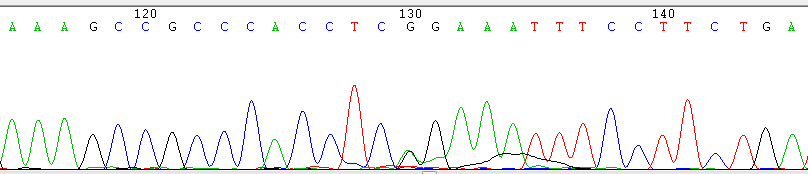


| CDKN2A | 21968732 | C | T |
| --- | --- | --- | --- |


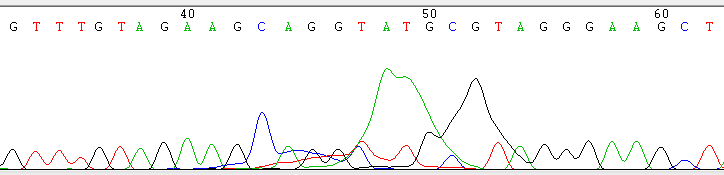


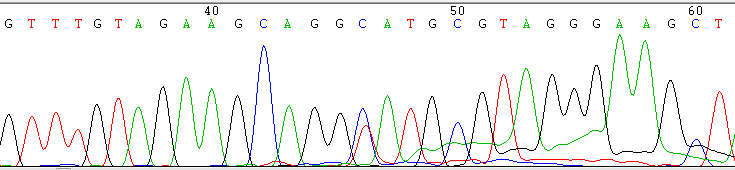


| CDKN2A | 21971015 | G | T |
| --- | --- | --- | --- |


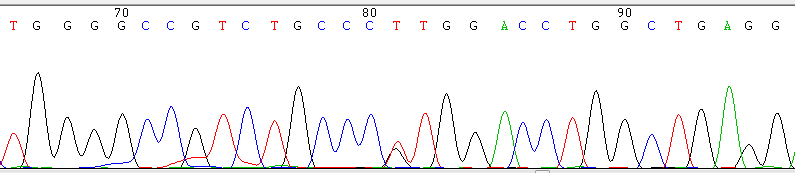


| CDKN2B | 22008664 | C | G |
| --- | --- | --- | --- |


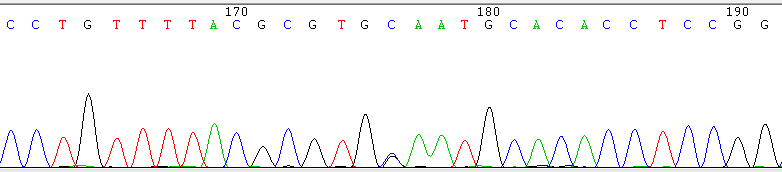


| MEN1 | 64572161 | C | T |
| --- | --- | --- | --- |


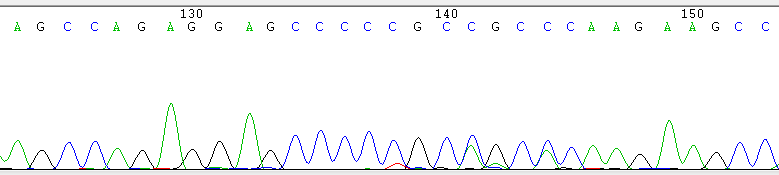


| MEN1 | 64572500 | G | A |
| --- | --- | --- | --- |


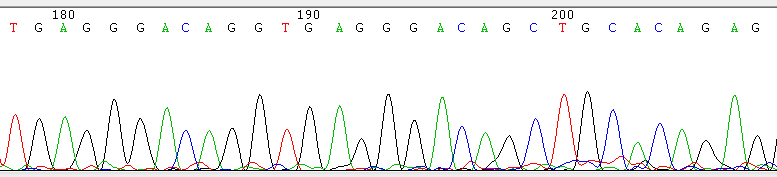


| MEN1 | 64572511 | G | T |
| --- | --- | --- | --- |


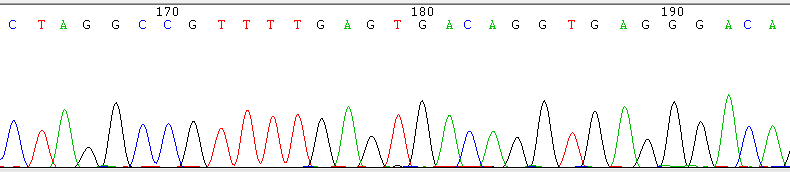


| MEN1 | 64573708 | C | T |
| --- | --- | --- | --- |


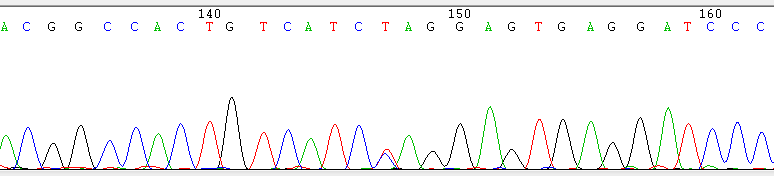


| MEN1 | 64577375-64577375 | C | - |
| --- | --- | --- | --- |


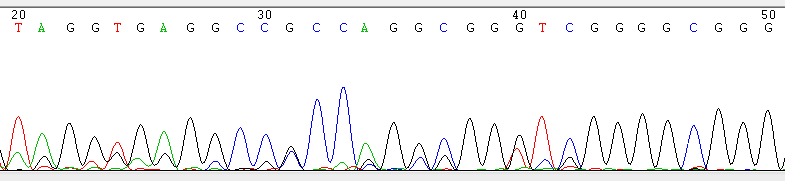


| MEN1 | 64577449 | G | T |
| --- | --- | --- | --- |


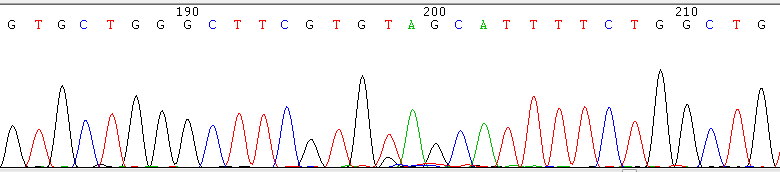


| MEN1 | 64575522-64575529 | GCAGGCCC | - |
| --- | --- | --- | --- |


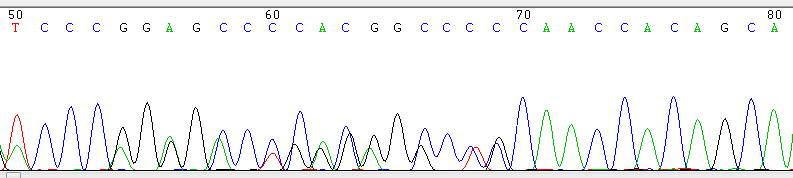


| CDKN1B | 12871053-12871053 | C | - |
| --- | --- | --- | --- |


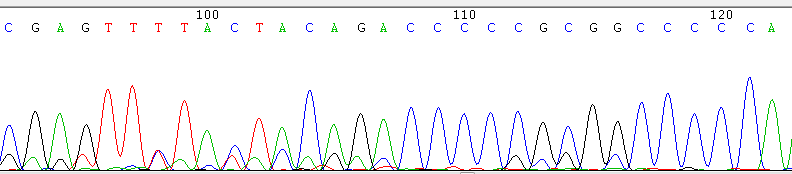


| HIC1 | 1960745 | G | A |
| --- | --- | --- | --- |


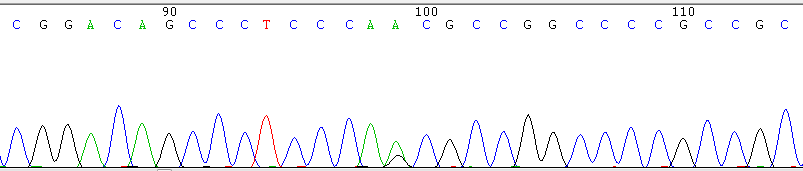


| HIC1 | 1961186 | C | T |
| --- | --- | --- | --- |


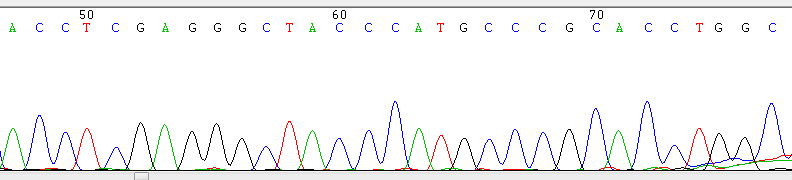


| HIC1 | 1961201 | C | T |
| --- | --- | --- | --- |


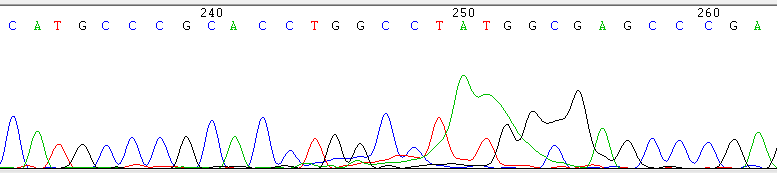


| HIC1 | 1961986 | G | A |
| --- | --- | --- | --- |


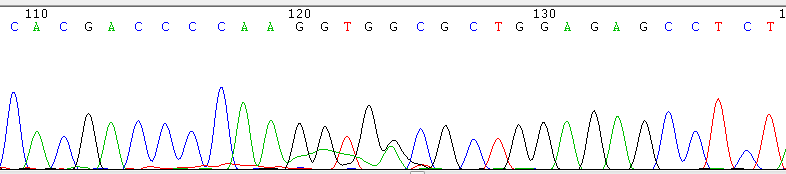


| HIC1 | 1960405-1960407 | GGC | - |
| --- | --- | --- | --- |


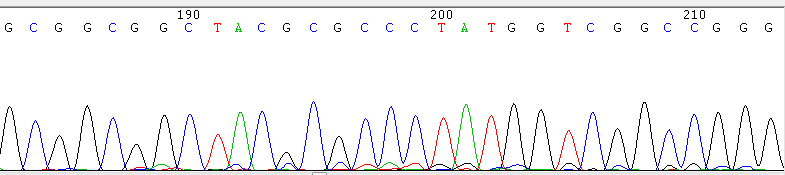


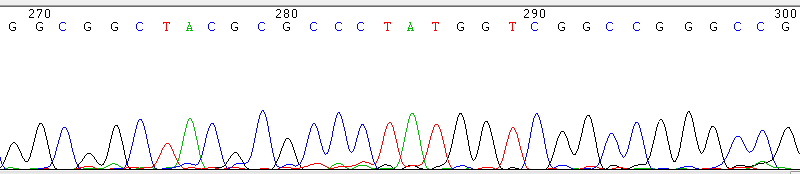

Supplement: Supplementary 2 — Supplemental Table 1: read depth and quality assessment of the targeted sequencing data in each lesion. Supplemental Table 2: details all alterations in the parathyroid tumor samples. Supplemental Table 3: clinical features of patients with PC from the study cohort with called genomic variants, reference sequences, and SNP-ID. Supplemental Table 4: clinical features of patients with PA from the study cohort with called genomic variants, reference sequences, and SNP-ID. [file 6666257.f2.docx]
